# Access to Microstructurally Complex Block Copolymers via Switchable Ring-Opening Polymerization of Cyclic Ester Mixtures

**DOI:** 10.1021/acs.macromol.5c00822

**Published:** 2025-07-02

**Authors:** David J. E. Seed, Amelia B. Milner, George R. Walker, Rachel H. Platel

**Affiliations:** Department of Chemistry, 4396 Lancaster University, Lancaster LA1 4YB, U.K.

## Abstract

The synthesis of sequence-controlled block copolymers from a mixture of cyclic ester monomers represents a significant challenge. In this study, we demonstrate the selective and self-switchable ring-opening polymerization (ROP) of three different cyclic ester monomers (lactide (LA), *rac*-β-butyrolactone (*rac*-β-BL) and ε-caprolactone (ε-CL)) to block copolymers of the form (AB)_*x*_(BC)_*y*_ using a highly selective yttrium catalyst. Each block contains units from two monomers and completely excludes one monomer. The switch from formation of one block to the next is strictly controlled by the presence or absence of LA in the reaction mixture rather than relative concentrations of each monomer. Individual block length and composition are determined by the initial monomer concentrations.

## Graphical abstract

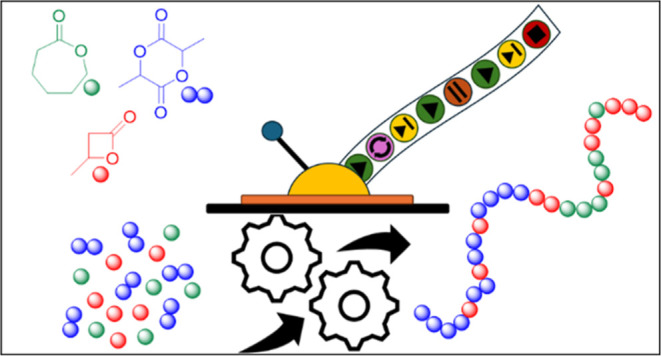

## Introduction

Precise control over polymer microstructure is a longstanding goal in the field of polymer synthesis, as it is essential to tuning properties.^1^ Biodegradable polymers and copolymers, specifically those derived from cyclic esters, continue to attract interest as degradable alternatives to current commodity polymers^2,3^ and in medicine,^4,5^ and have widespread applications.^6,7^ Block copolymers are particularly important:^8^ where microphase separation of the constituent blocks occurs, a material with new properties results, e.g. in a thermoplastic elastomer.^9^

In a metal catalyzed living or controlled polymerization system, a block copolymer can be prepared by sequential addition of monomers to the reaction mixture. Switchable polymerization systems have emerged as an attractive method to synthesize block copolymer structures, allowing for a one-pot reaction where all monomers are present from the start. These systems can be externally switchable,^10^ whereby a stimulus (light, heat, electrochemical) causes a change in catalyst selectivity; or internally, self-switchable systems, where the catalyst selects between different polymerization cycles due to the presence or absence of monomers in the reaction mixture and/or the identity of the growing polymer chain-end.^11,12^ This concept was first demonstrated by Coates and co-workers in the terpolymerization of anhydride, CO_2_ and cyclohexene oxide (CHO),^13^ and Williams and co-workers with the polymerization of CO_2_, CHO and ε-CL,^14^ and the approach has subsequently been used to prepare a range of polycarbonate-based polymers.^15–20^ Examples of switchable polymerizations using only cyclic ester mixtures are very rare^21,22^ and, there are no examples involving more than two cyclic esters.

Amine bis­(phenolate) supported yttrium complexes are well-established as stereoselective catalysts in *rac*-LA,^23–28^ *rac*-β-BL,^29–33^ ε-CL^34^ and other cyclic ester^35–42^ ROP but are less well explored in copolymerization reactions of these monomers.^43–48^ Our interest in controlling aliphatic copolyester microstructure and understanding the influence of the catalyst led us to report the copolymerization of *rac*-LA and *rac*-β-BL using an yttrium amine bis­(phenolate catalyst).^49^ Here we report **1** (Figure 1), as a highly selective catalyst that switches between the copolymerization of three cyclic ester monomers. The catalyst discriminates completely between three commercially available cyclic esters to deliver aliphatic block copolyesters with complex, yet controllable structures.

**Figure 1:**
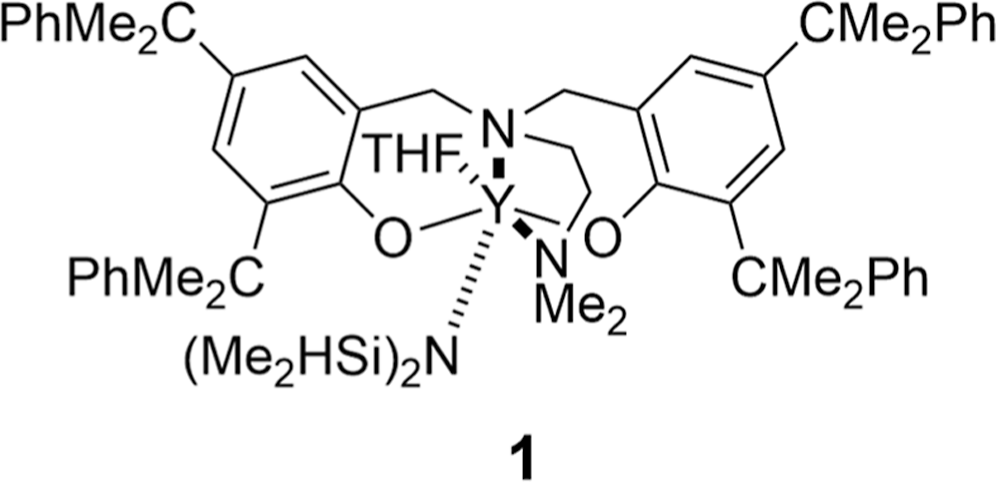
Structure of **1**.

## Results and Discussion

A polymerization reaction containing equimolar amounts of *rac*-LA, β-*rac*-BL and ε-CL, catalyzed by **1** at an overall catalyst/monomer ratio of 1:200 in toluene at ambient temperature was conducted (Table 1, entry 1). Over 2 h, the *rac*-LA and *rac*-β-BL reached full conversion and the conversion of ε-CL was 93%, as established by ^1^H NMR spectroscopy. The polymer obtained was fully characterized by ^1^H, ^13^C NMR and 2D NMR spectroscopy, gel-permeation chromatography (GPC) and differential scanning calorimetry (DSC). All data obtained demonstrate that a single polymer is produced, consisting of two distinct polymer blocks. The first block contains lactidyl (LL) and 3-hydroxybutyrate (B) units only, with no caproyl (C) units; the second block contains only B and C units, and no LL units. The B unit is common to both polymer blocks; and there is complete exclusion of C units in the first-formed block and complete exclusion of LL units in the second-formed block. The overall polymer composition, calculated from the ^1^H NMR spectrum corresponds very closely to the monomer feed ratio (33% LA/33% CL/33% BL, see ESI).

**Table 1:** One-Pot ROP of *rac*-LA, *rac*-β-BL and ε-CL Catalyzed by 1 to Prepare (LLB)_*x*_(BC)_*y*_ Block Copolymers^b^

entry	[*Rac*-LA]_0_/[*rac*-β-BL]_0_/[ε-CL]_0_/[1]	conv. *rac*-LA (%)^c^	conv. *rac*-β-BL (%)^c^	conv. ε-CL (%)^c^	isolated polymer yield (%)	polymer composition (LL/B/C) (mol %)^d^	composition of LLB block (LL/B) (mol %)^e^	composition of BC block (B/C) (mol %)^e^	*M*_n_ (kg mol^–1^)^f^	*M*_n_ (kg mol^–1^)^g^	*Đ* ^f^
1[Table-fn t1fn10]	66/66/66/1	>99	>99	93	65	33:34:33	75:25	40:60	22.0	19.0	1.24
2	25/25/25/1	>99	97	95	61	30:35:35	66:34	40:60	12.8	15.7	1.16
3	50/50/50/1	>99	83	75	67	34:37:29	63:37	40:60	15.0	15.4	1.12
4^h^	100/100/100/1	>99	97	89	72	33:33:34	73:27	41:59	32.7	46.9	1.07
5^h^	150/150/150/1	>99	95	87	64	33:34:33	74:26	39:61	48.5	49.3	1.08
6^i^	200/200/200/1	>99	93	84	68	35:33:32	74:26	37:63	63.7	53.8	1.15
7^i^	300/300/300/1	>99	83	73	59	38:29:33	75:25	41:59	89.1	66.2	1.28
8	33/133/33/1	>99	>99	97	86	17:66:17	59:41	77:23	19.7	37.7	1.06
9	33/33/133/1	>99	>99	98	86	17:17:66	73:27	17:83	22.2	20.6	1.23
10	133/33/33/1	>99	94	12	57	83:15:2	83:17	-	22.1	11.8	1.09

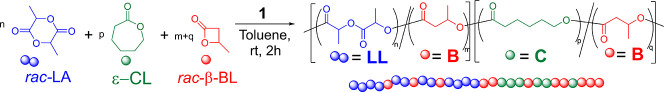

a Reaction conditions: Unless otherwise noted, total monomer = 6 mmol in 8 mL toluene at ambient temperature, reaction time 2 h.

b Determined from ^1^H NMR spectrum of crude reaction mixture.

c Determined from ^1^H NMR spectrum of polymer after workup, by integration of signals at 5.32–5.04 ppm, 4.06 ppm and 2.82–2.43 ppm.

d Determined from ^1^H NMR spectrum of polymer after workup.

e Theoretical number-average molecular weight (*M*_n_), calculated based on the monomer/I ratio and the percentage conversion of each monomer.

f Determined by GPC-MALLS at 40 °C in CHCl_3_ using d*n*/d*c* values of 0.024 (PLA), 0.033 (P3HB) and 0.11 (PCL) and using d*n*/d*c* (copolymer) = (0.033 × weight fraction B/100) + (0.024 × weight fraction LL/100) + (0.110 × weight fraction C/100).

g Reaction time 3 h.

h Reaction time 4 h.

i Polymer conversion determined after solvent removal.

The progress of a polymerization reaction was followed by ^1^H NMR spectroscopy ([ε-CL]_0_ = [*rac*-β-BL]_0_ = [*rac*-LA]_0_ = 0.25 M and [**1**]:[ε-CL + *rac*-β-BL + *rac*-LA] = 1:600 in toluene). Aliquots were removed from the reaction mixture at regular intervals and the percentage conversion of each monomer determined (Figure 2A). *Rac*-LA reacts rapidly in the first stage of the reaction, reaching 99% conversion over 25 min. *Rac*-β-BL reaches 32% conversion in this time, but no conversion of ε-CL is observed. After full conversion of *rac*-LA, the rapid consumption of *rac*-β-BL and ε-CL occurs between 25 and 35 min, reaching 88% and 65% conversion, respectively (*rac*-LA conversion is maintained at ≥ 99%). By 60 min, *rac*-β-BL and ε-CL have reached 96% and 85% conversion, respectively. The monitoring of this reaction clearly demonstrates a two-stage reaction, whereby the presence of *rac*-LA fully suppresses the reaction of ε-CL (and also hinders the rate of *rac*-β-BL reaction), and only when [*rac*-LA] = 0 does ε-CL react. To ensure that the reaction monitoring data is reflected in overall copolymer composition (i.e., that low molecular weight homopolymers are not being formed), samples were removed from a reaction and purified and compared by NMR spectroscopy to crude samples. The yields of these purified samples were high and their composition very closely matched those of crude samples (Table S2).

**Figure 2:**
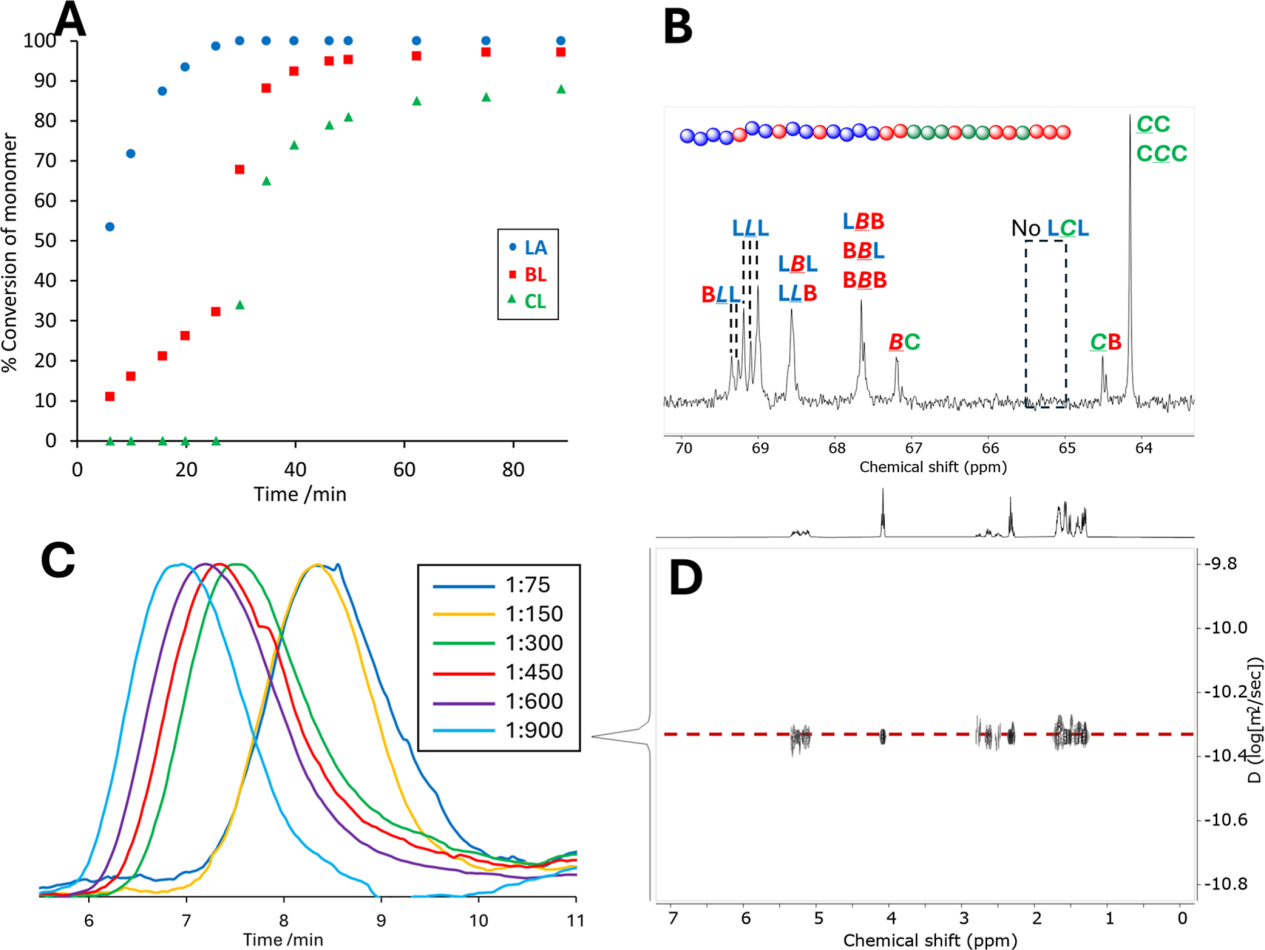
(A) Plot of percentage monomer conversion vs time, determined by ^1^H NMR spectroscopy. (B) Methine/methylene region of the ^13^C NMR spectrum of a polymer prepared with 1 (Table 1, entry 1), showing the diads and triads present (L = lactate, B = 3-hydroxybutyrate, C = caproyl) and the absence of L *C* L triads (C) GPC traces of polymers prepared using 1 (Table 1, entries 2–7) (D) DOSY NMR spectrum of a polymer (Table 1, entry 2).

Analysis of ^1^H and ^13^C NMR spectra of the copolymers allows the microstructure to be fully understood.^49–52^ In the ^1^H NMR spectrum of the polymer (Figure S9), the chemical shift of the OCH_2_ protons in a C unit is sensitive to the identity of its neighboring monomer: a CL unit gives rise to a signal at 4.14 ppm, while a CC or CB unit gives a signal at 4.06 ppm.^50^ The absence of any peak at 4.14 ppm confirms ε-CL is excluded from the LL-containing block of the polymer.

The ^13^C­{^1^H} NMR spectrum of the polymer provides further evidence for the catalyst selectivity; the region between 60 and 70 ppm is particularly informative (Figure 2B). Signals arising from nuclei in both the LLB and BC block of the polymer can be identified by comparison to literature data. The signal at 64.3 ppm is assigned to a C unit O*C*H_2_ carbon in a *C* C diad; two signals at 64.6 ppm correspond to CB diads (CCB and BCB); signals at 67.3 ppm are assigned to BC diads (BBC and CBC), while signals at 67.7 ppm are assigned to BB diads.^52^ Signals from methine carbon nuclei in the LA-BL block of the polymer are similarly assigned: at 69.5, 69.4, and 69.3 (BLL sequences), at 69.2 and 69.1 ppm (LLL sequences).^49^ The signal at 68.7 ppm is assigned to LBL and LLB linkages. The signals at 67.78 and 67.74 ppm contain BBB, BBL and LBB sequences. Significantly, no signals are observed in the region 65.1–65.3 ppm, where CL, LC and LCL sequences are found.^53^

In summary, the ^1^H and ^13^C NMR data supports the proposed polymer structure due to both the presence of peaks representing LB/BL and BC/CB heterodiads, and the absence of peaks representing LC/CL diads.

Other characterization data obtained strongly suggest that a single block copolymer is formed rather than multiple homo- or copolymers. GPC-SEC analysis of all polymers gives monomodal traces, with narrow to moderate dispersities (Figures 1C and S31–S40). Polymerizations were conducted at a range of [Y]­[monomer] ratios, from an overall ratio of 1:75 up to 1:900 (Table 1, entries 2–7). Over these catalyst/monomer ratios dispersities are <1.3, demonstrating the high level of control the catalyst exerts over the polymerization reaction.[Fn fn1] However, there is some deviation of the experimentally determined polymer *M*_n_ values from calculated values based on the catalyst/monomer ratio; at high catalyst loadings the *M*_n_ values are higher than calculated and, as catalyst loading increases the *M*_n_ becomes lower than the calculated values. This may indicate different behavior of the copolymers in solution than the constituent homopolymers. Furthermore, a ^1^H DOSY NMR spectrum shows a species with a single diffusion coefficient (Figure 1D), which supports an overall block structure rather than multiple homo- or copolymers. To further determine whether there was any contamination of the copolymer by homopolymer, a copolymer was spiked with varying amounts of lower molecular weight samples of either poly­(lactide) (PLA), poly­(caprolactone) (PCL) or poly­(3-hydroxybutyrate) (P3HB) and ^1^H DOSY spectra acquired (Figures S62–S75). Signals corresponding to both the copolymer and the homopolymer are visible in the spectra. The diffusion coefficient of the copolymer shows a small change upon addition of small amounts of each homopolymer (e.g., log D increases from −10.5 to −10.6 m^2^/s, Tables S3–S5), meaning that small amounts of homopolymer contamination cannot be ruled out.^54^

The negligible effect of transesterification was established by allowing a polymerization reaction to proceed for 24 h. Comparison of ^1^H and ^13^C NMR spectra of samples removed from the reaction at 2 and 24 h were almost identical (Figure S56), suggesting that very little or no transesterification occurs even after an extended reaction time, when all monomers have reached maximum conversion, and the fidelity of the two blocks is maintained.

Different monomer feed ratios were investigated, to determine the effect on catalyst behavior and polymer composition (Table 1, entries 8–10). Reactions were conducted with one monomer present in excess in each case (i.e., monomer ratios of 17:17:66, monomer concentrations 0.5 and 0.125 M, overall monomer concentration of 0.75 M) and monomer conversion over time was monitored by ^1^H NMR spectroscopy (Figures S47–S49). When [*rac*-β-BL] ≥ [*rac*-LA] in the monomer feed (entries 8 and 9), the unique catalyst behavior in this three-component reaction is preserved: the polymer obtained consists of two blocks; B units are present in both blocks but LL and C units are not found in the same block. With *rac*-β-BL in excess (entry 8), *rac*-LA and *rac*-β-BL react exclusively during the first 30 min of the reaction, until all *rac*-LA is consumed. Only then does rapid reaction of ε-CL commence with the remaining *rac*-β-BL present. It is notable that even with ε-CL present in considerable excess in the monomer feed (entry 9) its conversion remains at 0% until [*rac*-LA] = 0. These data confirm that the reaction of monomers in this system is not governed simply by their relative reactivity and concentration in solution. Rather, the presence or absence of *rac*-LA strictly controls both if ε-CL undergoes reaction and the rate of reaction of *rac*-β-BL.

Different catalysts were compared to elucidate the influence of **1** on monomer reactivity and polymer microstructure (Figure 3 and Table 2). Use of homoleptic species Y­(N­(SiHMe_2_)_2_)_3_(THF)_2_ in the polymerization reaction leads to reaction of *rac*-LA only, with only trace reaction (<5%) of either ε-CL or *rac*-β-BL over 2 h (Figure S41). Complexes **2–4** were prepared to probe the separate influence of the steric bulk around the metal (*R = t*Bu vs CMe_2_Ph in the *ortho*-position of the phenolate ring) and the neutral donor group (X = OMe vs NMe_2_, Figure 3). Both groups affect the copolymerization reaction. However, in all cases the overall reactivity pattern is similar: *rac*-LA > *rac*-β-BL > ε-CL. Using **2**, with R = *t*Bu, the reaction of *rac*-LA is extremely rapid, reaching >98% conversion within 10 min (100% conversion within 15 min, Table 2, entry 1 and Figure S42), compared to 35% conversion using **1** (65% at 15 min). No reaction of *rac*-β-BL occurs during this time. Only after *rac*-LA has fully polymerized do *rac*-β-BL and ε-CL react, and their conversion plateaus at ∼60% for CL and ∼50% for BL. A block copolymer is formed, consisting of a PLA block and a poly­(3-hydroxybutyrate-*co*-caprolactone) block. Thus, the R = CMe_2_Ph groups in the complex are required to achieve complete conversion of *rac*-β-BL and ε-CL.

**Figure 3:**
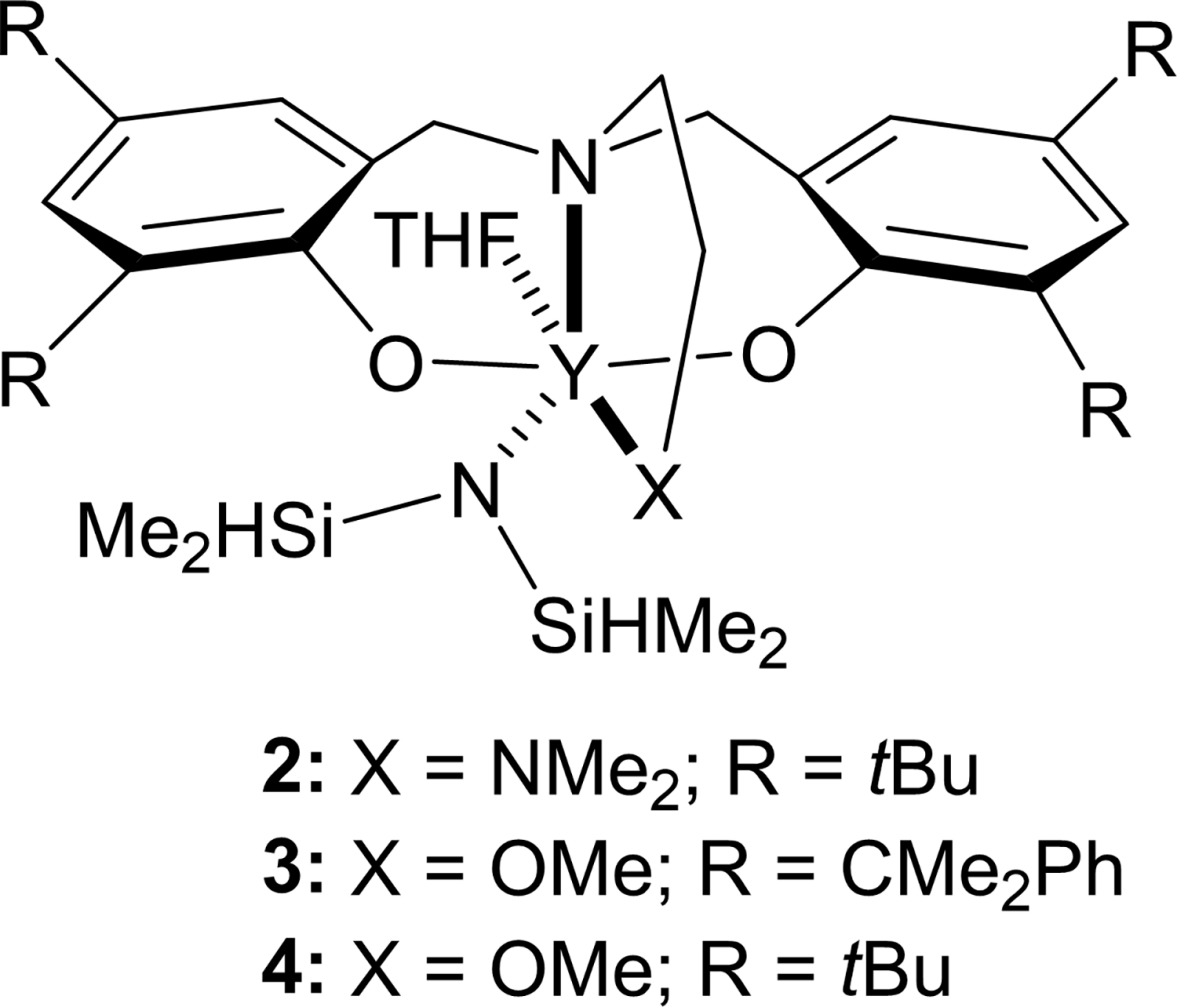
Structures of **2–4**.

**Table 2:** One-Pot ROP of *rac*-LA, *rac*-β-BL and ε-CL Catalyzed by **2–4**.^a^

entry	catalyst	conv. *rac*-LA (%)^b^	conv. *rac*-β-BL (%)^b^	conv. ε-CL (%)^b^	isolated yield (%)	*M*_n_(calc) (kg mol^–1^)^c^	*M*_n_ (kg mol^–1^)^d^	*Đ* ^d^
1	**2**	>99	53	61	93	17.3	22.4	1.19
2	**3**	>99	94	62	81	19.7	44.2	1.06
3	**4**	>99	14	11	86	11.2	38.1	1.19

a Reaction conditions: [*rac*-LA]_0_ = [ *rac*-β-BL]_0_ = [ε-CL]_0_) = 0.25 M in 8 mL toluene at ambient temperature, [Monomer]_0_/[**Y**] = 200, reaction time 1 h.

b Determined from ^1^H NMR spectrum of crude reaction mixture.

c Theoretical number-average molecular weight (*M*_n_), calculated based on the monomer/I ratio and the percentage conversion of each monomer.

d Determined by GPC-MALLS at 40 °C in CHCl_3_ using d*n*/d*c* values of 0.024 (PLA), 0.033 (P3HB) and 0.11 (PCL) and using d*n*/d*c* (copolymer) = (0.033 × weight fraction B/100) + (0.024 × weight fraction LL/100) + (0.110 × weight fraction C/100).

When NMe_2_ is replaced by OMe in the X position and R = CMe_2_Ph, the conversion of ε-CL only reaches 62% over 2 h (Table 2 entry 2 and Figure S44). There is an obvious difference in reactivity patterns between **3** and **4**: when CMe_2_Ph groups are replaced by *t*Bu groups in the R position with X = OMe, almost no reactivity of either *rac*-β-BL or ε-CL is observed (<15% conversion of both monomers) after the very rapid reaction of *rac*-LA (compare Figures S44 and S46). Considering these results together, it is clear that the ligand in **1** provides a particular environment around yttrium that facilitates the unique reactivity observed. It is only with the combination of X = NMe_2_ and R = CMe_2_Ph in the catalyst that high conversions of all monomers are observed.

It is already known that the NMe_2_ group in the X position promotes *rac*-β-BL ROP vs the OMe group,^36^ and that CMe_2_Ph groups in the *ortho*-position of the phenolate ring promote rapid, highly stereoselective ROP of *rac*-β-BL in toluene,^55,56^ while steric bulk around the metal center impedes rate of reaction of *rac-*LA.^29,49^ Focusing on the first stage of the reaction, it is proposed that using complexes **1** and **3,** with R = CMe_2_Ph, the coordination and/or ring-opening of *rac*-LA is somewhat inhibited compared to when **2** and **4** are used (with R = *t*Bu), due to the increased steric bulk around the metal (although it is still the monomer that reacts most rapidly). The reaction of *rac*-β-BL is promoted, when compared to complexes **2** and **4,** although its rate of reaction is still lower than that of *rac*-LA.

It was of interest to determine the microstructure of the individual blocks in these novel block copolymers. In the copolymerization of *rac*-LA and *rac*-β-BL using **1**, it is shown that, in the presence of any *rac*-LA, no BB homodiads are formed, i.e. after ring-opening of *rac*-β-BL, the next inserted monomer will always be *rac*-LA, if present.^49^ Only after the full consumption of *rac*-LA do multiple sequential *rac*-β-BL insertions occur. To confirm that this reactivity pattern is maintained in the presence of ε-CL, a reaction comprising equimolar amounts of *rac*-LA, *rac*-β-BL and ε-CL with ([*rac*-LA]_0_ = [ *rac*-β-BL]_0_ = [ε-CL]_0_)/[**1**] = 600 in toluene was conducted. In the ^1^H NMR spectrum of an aliquot taken from the reaction mixture after 30 min when conversion of *rac*-LA is 97% (*rac*-β-BL conversion = 37%, ε-CL conversion = 0%), all signals arising from the CH_2_ moiety in B units appear centered at 2.75 and 2.60 ppm, corresponding to BL diads and there is no signal at 2.46 ppm, which would indicate a BB diad. In the ^13^C NMR spectrum of this sample, signals are present for L centered triads (BLL, LLL, LLB) and the LBL triad, but there is no signal at 67.6 ppm, confirming the absence of LBB, BBL and BB/BBB sequences (i.e., any signal indicating sequential insertion of two or more *rac*-β-BL monomers) when *rac*-LA monomer is present (Figure S57). Having established that the LL/B block contains no BB homodiads, it is possible to reliably determine the microstructure of the C/B block from ^13^C NMR spectra; it is notable that peaks representing heterodiads (BC and CB) have lower intensity than those peaks representing homodiads (BB and CC), indicating that the microstructure of this polymer block deviates from statistical.

Given the absence of reactivity of **1** toward ε-CL in the presence of LA, an experiment was designed to test whether the addition of *rac*-β-BL can ′switch-on′ reactivity of ε-CL. **1** was reacted with an equimolar mixture of *S,S*-LA and ε-CL (ratio of **1**:monomers = 1:200 overall, [LA]_0_ = [CL]_0_ = 0.33 M) and aliquots were removed from the reaction mixture at regular intervals and analyzed by ^1^H NMR spectroscopy (Figure 4). Within 30 min the *S,S*-LA reaches >98% conversion and ε-CL reaches <3%. There is evidence of epimerization of the *S,S*-LA during the polymerization, consistent with our previous study.^49^ The reaction was left to react for a further 60 min and ε-CL reacts slowly to 10% conversion during this time. At *t* = 90 min, 200 equiv. *rac*-β-BL was added into the reaction mixture. Both the *rac*-β-BL and ε-CL convert very rapidly within the next 6.5 min to reach conversions of 84% and 69% respectively. GPC data indicate that a single polymeric species is formed in this reaction, i.e. that the *rac*-β-BL and ε-CL form a copolymer with the PLA block rather than a separate copolymer or homopolymers.

**Figure 4:**
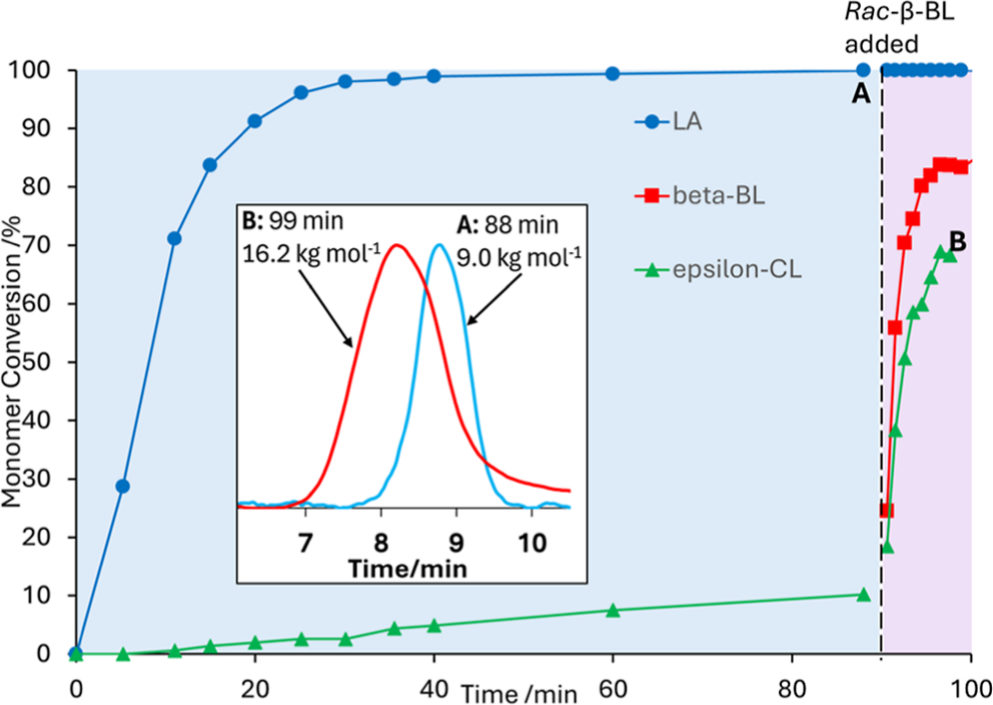
Main plot: Monomer conversion over time in a polymerization reaction, initially of *S,S*-LA (blue circles) and ε-CL catalyzed (green triangles) by **1** (blue region). *Rac*-β-BL (red squares) is added to the reaction mixture after 90 min (black dashed line and pink region). Inset: GPC traces of aliquots removed from the reaction mixture after 88 min (point A, blue trace) and 99 min (point B, red trace).

Furthermore, even the presence of very low concentrations of LA ′switch-off′ catalyst reactivity of **1** toward ε-CL and moderate reactivity toward *rac*-β-BL. A reaction between **1** and an equimolar mixture of *rac*-β-BL and ε-CL (ratio of **1**: monomers = 1:400 overall, [BL]_0_ = [CL]_0_ = 0.5 M) was initiated and monitored by ^1^H NMR spectroscopy (Figure 5). The *rac*-β-BL and ε-CL rapidly react and reach 31% and 15% conversion, respectively, in under 3 min. *S,S*-LA (0.5 mmol) was added at *t* = 3 min and begins to react. The ε-CL immediately ceases reacting and conversion stalls at 17%. The *rac*-β-BL continues to react, but at a much slower rate, reaching 43% over the next 30 min. When [*S,S*-LA] reaches 0, both *rac*-β-BL and ε-CL begin to rapidly react once again, reaching 92% and 76% conversions after a further 4 min and maximum conversions of >99% (*rac*-β-BL) and 96% (ε-CL) after 50 min total reaction time.

**Figure 5:**
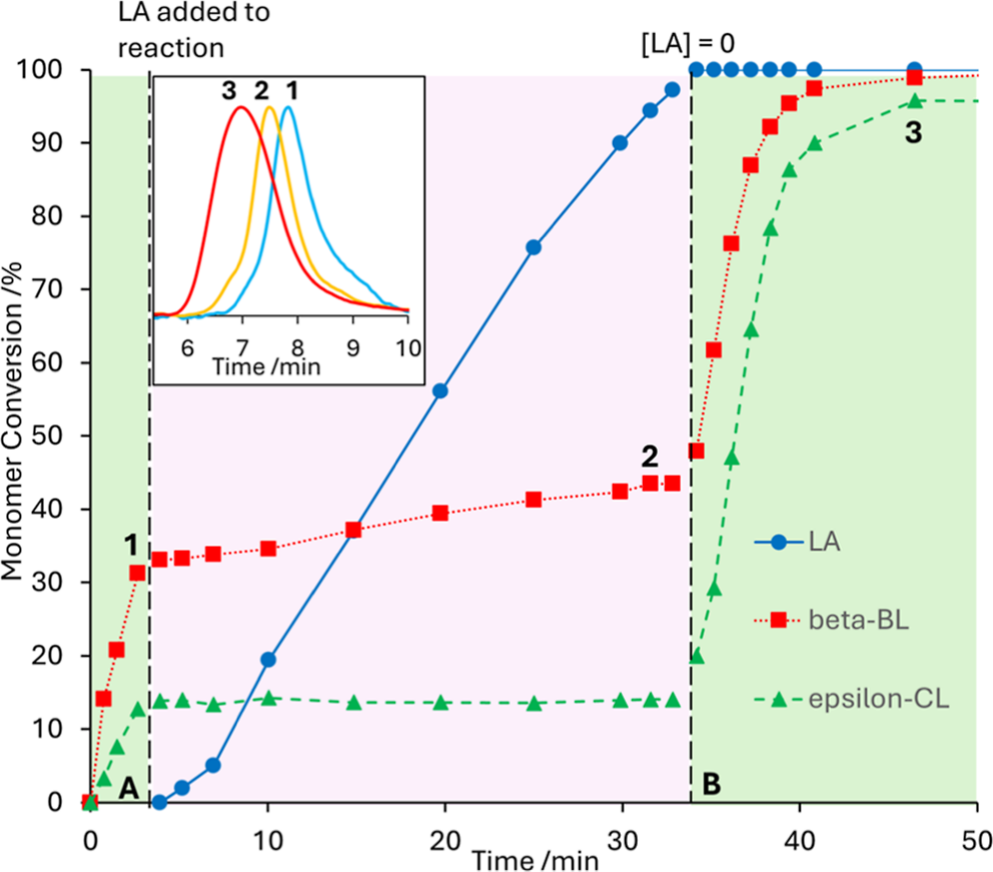
Main plot: Conversion over time in a polymerization reaction, initially of *rac*-β-BL and ε-CL catalyzed by **1**. Green region: consumption of *rac*-β-BL and ε-CL; point A: S,S-LA added to the reaction mixture; pink region: consumption of LA; point B: [LA] = 0; green region: consumption of *rac*-β-BL and ε-CL. Inset: GPC traces of aliquots removed from the reaction mixture after 2.6 min (point 1, blue trace), 31.5 min (point 2, yellow trace) and 50.5 min (point 3, red trace).

The ability of a low concentration of LA to completely halt the reaction of ε-CL and severely retard the rate of reaction of *rac*-β-BL, and the ability of *rac*-β-BL to initiate rapid reactivity of ε-CL in the presence of PLA lead us to further probe the system in order to elucidate the reaction mechanism. The progress of separate homopolymerization reactions of each monomer were followed by ^1^H NMR spectroscopy in toluene at ambient temperature, with [monomer]_0_ = 0.124 M and [monomer]_0_/[**1**] = 66.7. It was not possible to carry out homopolymerization reactions under the same conditions as used for copolymerization reactions due to the rapidity of reaction of ε-CL, the insolubility of poly­(3—hydroxybutyrate) (P3HB) and the insolubility of *rac*-LA in toluene at this concentration (in the copolymerization reaction the presence of the other monomers solubilizes *rac*-LA and P3HB is not formed). In the homopolymerization reactions, the relative rates of reaction of the monomers is ε-CL > *rac*-β-BL ≫ *rac*-LA, which is completely different to monomer reactivities of *rac*-LA > *rac*-β-BL ≈ ε-CL in the copolymerization reaction. The homopolymerization reactions all follow a pseudo first-order rate law, with *k*_obs_ values of 0.29 min^–1^ for ε-CL, 0.10 min^–1^ for *rac*-β-BL and 0.009 min^–1^ for *rac*-LA, respectively (Figure 6). A reversal in reactivity between LA and ε-CL in the presence of each other compared to alone is well documented,^57–62^ but only beginning to be understood.^63,64^ The dramatic change in reactivity we observe clearly demonstrates that some specific interactions between the monomers, the catalyst, the growing polymer chain and/or the identity of the polymer chain end are responsible for the copolymer formation, rather than the inherent monomer reactivities.

**Figure 6:**
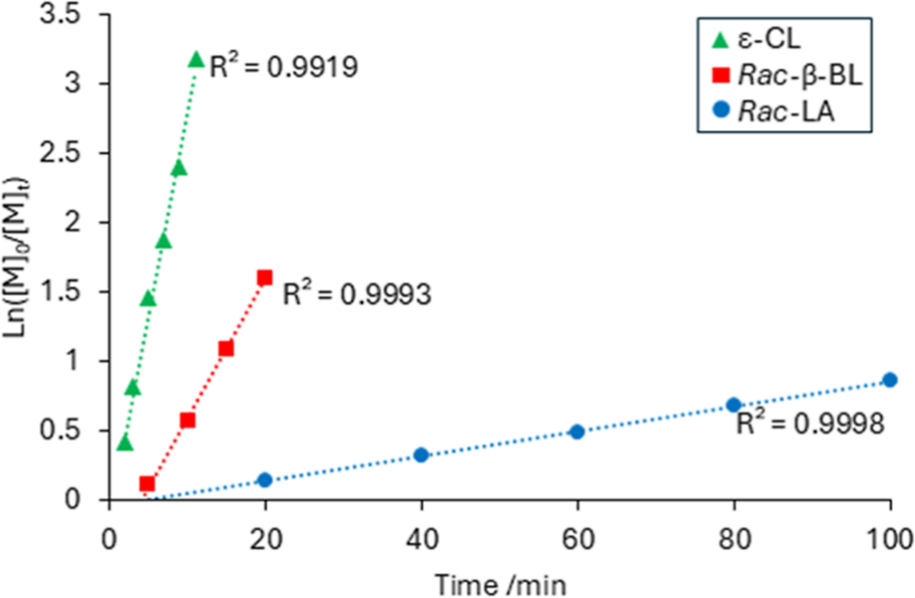
Overlaid plots to show semilogarithmic plots in the separate homopolymerization of ε-CL (green triangles), *rac*-β-BL (red squares) and *rac*-LA (blue circles) by **1** at [monomer]_0_ = 0.124 M and [monomer]_0_/[**1**] = 66.7 in toluene at ambient temperature.

Both the coordination of monomer to the metal and the ring-opening (insertion) step can influence the reactivity of a particular monomer in a copolymerization reaction. The opposite effects of decreasing electron donor ability of lactones with decreasing ring size, (suggesting that the carbonyl group on ε-CL is a stronger electron donor than that on *rac*-β-BL)^57,65^ and a steric influence (the larger size of ε-CL vs *rac*-β-BL impeding coordination to yttrium in a sterically crowded environment)^21^ have been invoked by others to explain reactivity differences between cyclic ester monomers. The comparative binding of lactones and LA to B­(C_6_F_5_)_3_ has also been studied and lactones have been found to bind more strongly than LA to this Lewis acid and even displace coordinated LA from B­(C_6_F_5_)_3_.^66^ We suggest it is unlikely that monomer coordination strengths are alone responsible for the reactivity reported, since this would lead to a reactivity trend similar to that observed in homopolymerizations (ε-CL > *rac*-β-BL > *rac*-LA) rather than that which we observe in the copolymerization reaction.

It is proposed that the identity of the growing polymer chain end and the specific reactivity of each monomer with the metal and last inserted monomer are responsible for the observed reactivity. Stoichiometric reactions on an NMR scale were conducted between **1** and mimics of polymer chain ends to better understand this interaction (Scheme 1). A previous study using a related yttrium complex concluded that the carbonyl groups of both α- and β-alkoxyesters coordinate to yttrium in stoichiometric reactions.^67^ A solution of **1** in C_6_D_6_ was mixed with an equimolar amount of (*S*)-ethyl lactate in C_6_D_6_ and analyzed by ^1^H and ^13^C NMR spectroscopy (Scheme 1 and Figures S58 and S59).

**Scheme 1:**
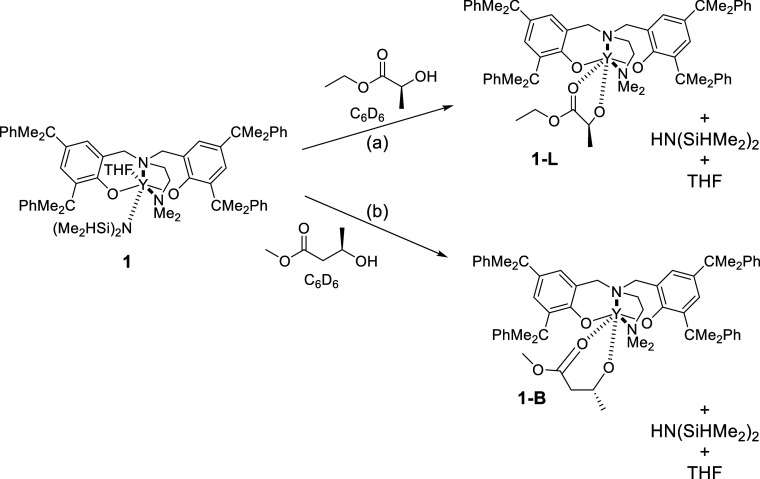
Reaction of **1** with (a) 1 eq. (*S*)-ethyl Lactate and (b) 1 eq. (*R*)-methyl-3-hydroxybutyrate

Signals assigned to one equivalent of 1,1,3,3-tetramethyl disilazane and THF are observed in the ^1^H NMR spectrum, which also contains a single major set of signals suggestive of the formation of a *C*_1_ symmetric yttrium species, assigned to **1-L**. The ^13^C NMR spectrum contains a single signal in the carbonyl region at 190.6 ppm, assigned to C=O in (*S*)-ethyl lactate. This is shifted significantly downfield from noncoordinated (*S*)-ethyl lactate, (176.2 ppm), which suggests that (*S*)-ethyl lactate binds in a κ-^2^ fashion to yttrium and a five-membered chelate is formed. It is proposed that when the polymer chain end is a lactate (L) unit, a five-membered chelate is formed such as in **1-L** can be formed.

The analogous reaction between **1** and (*R*)-methyl-3-hydroxybutyrate was also conducted (Figures S60 and S61). In this case the signals in the ^1^H NMR spectrum obtained were broader, indicating considerable fluxionality on the NMR time scale. However, it is still possible to identify signals for 1,1,3,3-tetramethyl disilazane and free THF, as well as a broad signal at 4.65 ppm assigned to the hydroxybutyrate methine hydrogen and a singlet at 3.31 ppm assigned to the OMe group. In the ^13^C NMR spectrum there is a single signal in the carbonyl region at 178.2 ppm (vs 172.5 ppm in (*R*)-methyl-3-hydroxybutyrate), which suggests coordination of the carbonyl group to yttrium. However, the presence of a low intensity doublet of quartets centered at 5.75 ppm, indicative of the formation of a species containing crotonate groups, even a very short time after preparing the sample, suggests some instability or reactivity of **1-B**.

At a stoichiometric level, it has been found with a related aluminum complex that LA inserts more easily into a six-membered metal β-alkoxybutyrate species than into a five membered metal *O*-lactate species.^68^ The absence of BB diads in the first copolymer block could be due to the high reactivity of LA with the yttrium β-alkoxybutyrate polymer chain end (vs *rac*-β-BL), with reactivity with the 5-membered yttrium *O*-lactate species being much more similar for *rac*-β-BL and LA.

In the first stage of the copolymerization reaction, *rac-*LA is fully consumed and its consumption follows first-order rate behavior, while consumption of *rac*-β-BL follows a zero-order rate law. These data indicate that insertion of LA into the growing polymer chain is rate-limiting and addition of *rac*-β-BL is rapid. A dependence of the presence of LA on reaction rate of *rac*-β-BL using **1** has previously been observed.^49^ The effect of *rac*-β-BL on *rac*-LA polymerization was further explored by comparing the pseudo first-order rate constants of *rac*-LA ROP in the presence of both *rac*-β-BL and ε-CL (*k*_obs_ = 0.23 min^–1^) and with only ε-CL (*k*_obs_ = 0.14 min^–1^), under otherwise identical conditions (Figures S51 and S52). The increase in rate of *rac*-LA polymerization of ∼50% when *rac*-β-BL is present suggests that the presence of *rac*-β-BL in the reaction mixture promotes *rac*-LA polymerization, which supports the theory that LA inserts more rapidly into the growing polymer chain *after* the insertion of *rac*-β-BL than after the insertion of LA.

The absence of any reactivity of ε-CL during the first stage of the polymerization reaction suggests that, when LA is present, either ε-CL coordinates to yttrium, but does not ring-open, or that no coordination to the metal occurs. We find that in the presence of ε-CL in toluene, the ROP of *rac*-LA proceeds with significant tacticity control to give heterotactic PLA (*P*_r_ = 0.92), while in the absence of ε-CL, PLA that is close to atactic is formed (*P*_r_ = 0.60, Figure S50). The high *P*_r_ value in the presence of ε-CL is only slightly lower than that obtained when *rac*-LA homopolymerization is carried out in THF, a coordinating solvent (*P*_r_ = 0.99), where the high heterotactic bias is attributed to solvent coordination to yttrium.^25^ This result provides evidence that ε-CL does coordinate to yttrium but does not ring open.

It was also useful to briefly consider the monomer conversions in copolymerization reactions of the two pairs of monomers that comprise the two polymer blocks (*rac*-LA/*rac*-β-BL and *rac*-β-BL/ε-CL), as a comparison to the one-pot reaction. The copolymerization of *rac*-β-BL and ε-CL was investigated at the same monomer concentration as the one-pot reaction to allow direct comparison ([*rac*-β-BL]_0_ = [ε-CL]_0_ = 0.25 M, ([*rac*-β-BL]_0_ + [ε-CL]_0_)/[**1**] = 400 in toluene at ambient temperature (we have previously reported the copolymerization of LA/BL with **1** ^49^). After an induction period, the consumption of *rac*-β-BL follows pseudo first-order kinetics under these conditions, while ε-CL conversion follows zero-order kinetics at this concentration (Figures S53–S55). This suggests that, in the absence of LA, the reaction of *rac*-β-BL is rate-limiting and the insertion of ε-CL is rapid.

Bringing all the data together, it is proposed that the identity of last inserted monomer is key in controlling the reactivity of the monomer mixture, as is the presence or absence of LA. The reactivity can be split into two different stages, corresponding to the formation of the two copolymer blocks: stage 1, when LA is present and stage 2, when LA is absent. In stage 1, when the last inserted monomer is *rac*-β-BL, the rate of LA insertion is extremely high and the rate of either *rac*-β-BL or ε-CL insertion is zero. When the last inserted monomer is LA, the rate of LA and *rac*-β-BL insertions are comparable and the rate of ε-CL insertion is zero. Once all LA has been consumed, insertion of a *rac*-β-BL monomer into the yttrium *O*-lactate species takes the polymerization to stage 2. With no LA present, the rates of insertion of either *rac*-β-BL or ε-CL into both the yttrium β-alkoxybutyrate polymer chain end and the yttrium *O*-caproyl chain end are high and comparable. However, reintroduction of any LA into the reaction mixture returns the polymerization immediately to stage 1.

The thermal properties of selected polymers were explored using DSC (Table S1). For a polymer with composition 33:34:33 (LL/B:C, Table 1, entry 1), two distinct glass transitions (*T*_g_) are observed, at 17.9 °C (*T*_g_1) and at −36.6 °C *(T*_g_2). *T*_g_1 is intermediate between the *T*_g_ of PLA and of P3HB, while *T*_g_2 is intermediate between the *T*_g_ of PCL and P3HB, indicating microphase separation between the two blocks of the polymer. For this polymer, no melting or crystallization of the material is observed in the temperature range −80 °C to 200 °C. However, for polymers with high proportions of B or C units, melting and crystallization transitions were observed.

In summary, we report an yttrium amine bis­(phenolate) catalyst that operates with excellent selectivity to prepare sequence-controlled block copolymers from mixtures of cyclic esters. The catalyst switches between LA/*rac*-β-BL copolymerization and *rac*-β-BL/ε-CL copolymerization, with the presence or absence of LA and *rac*-β-BL acting as dual switches. Block copolymers with a unique microstructure are obtained: a block of LL and B units excluding C, and a block of B and C units excluding LL. Depending on the initial monomer concentration, the composition of each block can be manipulated, and the thermal properties (*T*_m_/*T*_g_) reflect block compositions. The nature of the switch between ROP of the different monomer pairs was demonstrated—switching on ε-CL ROP by addition of *rac*-β-BL to a PLA/ε-CL mixture, switching off ε-CL ROP by adding *rac*-LA to a mixture of *rac*-β-BL/ε-CL undergoing polymerization. We envisage this system as an effective entry point to a range of stereo- and sequence-controlled polyesters with tunable properties that are not accessible by current methods.

## Experimental Section

### Materials and Methods

Metal precursors, complexes, and all polymers and copolymers were prepared under an inert (nitrogen) atmosphere using standard Schlenk or glovebox (Innovative Technologies) techniques. Dry solvents (toluene, hexane, tetrahydrofuran, dichloromethane) were obtained from an Inert SPS (solvent purification system). All solvents and chemicals were purchased from commercial suppliers (Sigma-Aldrich, Strem, Alfa Aesar, TCI) and used as received except where otherwise specified. *Rac*-LA and (*S,S*)-LA were recrystallized once from dry toluene and sublimed once under vacuum at 50 °C. ε-CL and *rac-*β-butyrolactone were stirred over CaH_2_ under N_2_ at 50 °C overnight, distilled under vacuum and degassed via 3 freeze–pump–thaw cycles. Benzene-*d*_6_ was dried over activated 4 Å molecular sieves and degassed via 3 freeze–pump–thaw cycles. Y­(N­(SiHMe_2_)_2_)_3_(THF)_2_,^69^ complexes **1** ^36^ and **2–4** ^25^ were prepared following literature procedures.

^1^H and ^13^C NMR spectra were recorded at 400.1 and 100.6 MHz, respectively, on a Bruker Avance III 400 or Bruker Neo 400 spectrometer equipped with a broadband-observe probe (BBFO). ^1^H NMR spectra are referenced to residual solvent signals; ^13^C NMR spectra are referenced to the signal for the solvent ^13^C nucleus. The temperature was calibrated using deuterated methanol, and unless stated otherwise, were all acquired at 298.0 K. Spectra were processed using either ACD/NMR Processor or Mestrenova. Chemical shifts are reported in ppm and coupling constants in Hz.

Molecular weights of polymers were determined by gel permeation chromatography (GPC) multiangle laser light scattering (MALLS) in chloroform using a Shimadzu liquid chromatograph equipped with a Shimadzu LC-20AD pump and autosampler, two Phenogel 5 μm linear (2) columns (300 × 7.8 mm), a Shimadzu RID-20A refractive index detector, a Wyatt miniDAWN treos LLS detector and a Wyatt ViscoStar-II viscometer. The column temperature was maintained at 40 °C and the flow rate was 1 mL min^–1^. Samples were dissolved in chloroform at an approximate concentration of 10 mg mL^–1^ and filtered prior to analysis. Data was processed using ASTRA software using d*n*/d*c* values of 0.024 (PLA), 0.110 (PCL) and 0.33 (P3HB).

Thermal properties of the polymers were determined by differential scanning calorimetry (DSC) using a Mettler Toledo DSC1 STARe instrument equipped with a Julabo FT900 intracooler. Polymer samples of known mass (generally 2–8 mg) were weighed into 40 μL aluminum standard pans. Samples were heated to 200 °C (at a rate of 10 °C min^–1^) and held at this temperature for 10 min, before being cooled to −70 °C (at a rate of −10 °C min^–1^) and held at this temperature for 10 min. The heating and cooling cycles were repeated once. The sample was returned to 25 °C (at a rate of 10 °C min^–1^). Data was processed using STARe software. Glass transitions (*T*_g_) and melting temperatures (*T*_m_) were taken from the second heating cycle and crystallization (*T*_c_) values were taken from the first cooling cycle.

### General Procedure for Polymerization Reactions

The required masses of ε-CL, *rac*-β-BL and *rac*-LA (6 mmol total) were added to a vial equipped with a stirrer bar and dissolved in toluene (6 mL). A solution of complex **1** (2 mL of a 0.015 M solution in toluene, 0.03 mmol) was added to the stirred monomer mixture. The reaction mixture was stirred at ambient temperature for 2 h. After 2 h the reaction vial was removed from the glovebox. A pipet was used to remove 1–2 drops of reaction mixture, which was added to approximately 0.7 mL chloroform and used for NMR analysis. The remaining reaction mixture was quenched with hexane (approximately 2–3 mL), causing polymer to precipitate as a colorless material. The solvents were removed using rotary evaporation, leaving a colorless residue. The crude product was dried in a vacuum oven overnight at 60 °C. The crude product was dissolved in the minimum amount of chloroform (approximately 5 mL) and the resulting solution was added dropwise to methanol (50 mL) with vigorous stirring. The precipitate that formed was collected by filtration under reduced pressure and the resulting polymer was dried in a vacuum oven at 60 °C overnight.

^1^H NMR (400 MHz, C_6_D_6_, 298 K) δ_H_, ppm: 5.37–5.04 (m, 3H, 2 × C*H* (LL unit), and 1 × C*H* (B unit)), 4.08 (t, *J* = 6.7 Hz, 2H, OC*H*_2_ (C unit)), 2.80–2.40 (m, 2H, CH_2_ (B unit)), 2.35–2.22 (m, 2H, C*H*_2_ (C unit)), 1.70–1.59 (m, 4H 2 × *C*H_2_ (C unit)), 1.57–1.45 (m, 6H, 2 × CH_3_ (LL unit)), 1.42–1.32 (m, (m, 2H, CH_2_ (C unit)), 1.32–1.23 (m, 3H, CH_3_).

^13^C NMR (100 MHz, C_6_D_6_, 298 K) δ_C_, ppm: 173.5 (CO, CL unit), 169.2 (CO, BL and LA units), 69.3–67.2 (CH, CL, BL and LA units), 64.5 (CH_2_, CL-BL, CL unit), 64.1 (CH_2_, CL–CL, CL unit), 40.7–40.0 (CH_2_, BL unit), 34.1 (CH_2_, CL unit), 28.4 (CH_2_, CL unit), 25.5 (CH_2_, CL unit), 24.6 (CH_2_, CL unit), 19.8–19.6 (CH_3_, LA unit), and 16.7 (CH_3_, BL unit).

### Procedure for Reaction Comparing Polymer Composition in Crude Aliquots and Purified Copolymer

*Rac*-LA (720 mg, 5.0 mmol), ε-CL (570 mg, 5.0 mmol) and *rac*-β-BL (430 mg, 5.0 mmol) were added to a vial equipped with a stirrer bar and dissolved in toluene (16 mL). A solution of 1,3,5-trimethoxybenzene (0.070 g, 0.42 mmol) in toluene (2 mL) was added to the monomer mixture. An aliquot (0.1 mL) was taken, and chloroform was added (0.4 mL). A solution of complex **1** (77 mg, 0.074 mmol) in toluene (2 mL) was added to the reaction at a known time. Samples (0.4 mL) were taken every 5 min and quenched with chloroform (0.8 mL). At 20, 30, 35, and 60 min, larger samples were taken (4.0 mL), an aliquot (0.4 mL) was taken and quenched with chloroform (0.8 mL) while the remainder of the sample was quenched with hexane (2.0 mL) and chloroform (2.0 mL). Samples were analyzed by ^1^H NMR spectroscopy to determine monomer conversion. Solvent was removed using rotary evaporation for samples at 20, 30, 35, and 60 min to give a colorless residue, the crude product was dried in a vacuum oven overnight at 60 °C and analyzed by NMR spectroscopy before and after purification (detailed in the general procedure for polymerization reactions), to determine polymer composition.

### Procedure for ′Switch-On′ Reaction

To two 28 mL vials equipped with stir bars were added (*S,S*)-LA (288 mg, 2 mmol), ε-CL (228 mg, 2 mmol), 1,3,5-trimethoxybenzene (14 mg) and toluene (6 mL). The vials were labeled ′A′ and ′B′. Separately, two solutions of **1** (21 mg, 0.02 mmol) in toluene (1 mL) in 2 mL vials were prepared and one solution of *rac*-β-BL (172 mg, 2 mmol) in toluene (1 mL) was prepared in a 7 mL vial. A 0.1 mL aliquot was removed from each of the (*S,S*)-LA/ε-CL monomer mixtures, which were removed from the glovebox and separately added to chloroform (0.8 mL). The solutions of **1** were added in quick succession (within 10 s) to each of the stirred (*S,S*)-LA/ε-CL monomer mixtures at a known time (*t* = 0 min). From reaction A, aliquots (0.3–0.4 mL) were withdrawn from the reaction mixture at regular intervals, removed from the glovebox and quenched into chloroform (0.8–1.0 mL) at known times. After a 60 min reaction time and an 88 min reaction time, aliquots were removed from both reactions A and B and quenched separately (within 20 s). At *t* = 90 min, the *rac*-β-BL solution was added to reaction B via syringe. Aliquots (0.3–0.4 mL) were withdrawn from reaction mixture B at regular intervals, removed from the glovebox and quenched into chloroform (0.8–1.0 mL) at known times. Each aliquot solution was transferred directly to an NMR tube and analyzed by ^1^H NMR using a solvent suppression experiment that suppresses solvent peaks from chloroform and toluene. The solvents were then allowed to evaporate from these samples. The samples were redissolved in CDCl_3_ and analyzed by ^1^H NMR spectroscopy. The solvents were then allowed to evaporate. The samples were then dried in a vacuum oven overnight to remove residual ε-CL monomer. Residual rac-LA and 1,3,5-trimethoxybenzene are also removed under these conditions. Selected samples were reanalyzed by ^1^H and ^13^C NMR spectroscopy and GPC.

### Procedure for ′Switch-Off′ Reaction

To a 28 mL vial equipped with stir bar were added *rac*-β-BL (344 mg, 4 mmol), ε-CL (456 mg, 4 mmol), 1,3,5-trimethoxybenzene (14 mg) and toluene (6 mL). A 0.1 mL aliquot was removed from this solution via a 1 mL syringe, removed from the glovebox and to chloroform (0.8 mL). Separately, a solution of **1** (21 mg, 0.02 mmol) in toluene (1 mL) in a 2 mL vial was prepared. Another solution was prepared of (*S,S*)-LA (72 mg, 0.5 mmol) in toluene (1 mL). The solution of **1** was added to the stirred monomer mixture at a known time (t = 0 min). Three aliquots (0.3–0.4 mL) were withdrawn from the reaction mixture at regular intervals, removed from the glovebox and quenched into chloroform (0.8–1.0 mL) at known times. At *t* = 3 min, the (*S,S*)-LA solution was added to the reaction mixture via pipet. An aliquot was removed immediately from the reaction mixture, removed from the glovebox and quenched into chloroform (0.8–1.0 mL) at known times. The process of aliquot removal was continued until *t* = 50 min. A visible increase in the viscosity of the reaction mixture indicated that all three monomers had reached high conversion. Each aliquot solution was transferred directly to an NMR tube and analyzed by ^1^H NMR using a solvent suppression experiment that suppresses solvent peaks from chloroform and toluene. The solvents were then allowed to evaporate from these samples. The samples were redissolved in CDCl_3_ and analyzed by ^1^H NMR spectroscopy. The solvents were then allowed to evaporate. The samples were dried in a vacuum oven overnight at 55 °C to remove residual ε-CL monomer. Residual rac-LA and 1,3,5-trimethoxybenzene are also removed under these conditions. Selected samples were reanalyzed by ^1^H and ^13^C NMR spectroscopy and GPC.

### NMR Scale Reaction of **1** with (*S*)-Ethyl Lactate

To a solution of **1** (0.038 g, 0.036 mmol) in C_6_D_6_ (0.5 mL) in a small vial was added a solution of (*S*)-ethyl lactate (0.004 g, 0.036 mmol) in C_6_D_6_ (0.25 mL). The resulting solution was gently shaken to mix, transferred to a J-Young NMR tube and analyzed by NMR spectroscopy within 30 min of sample preparation. The product was not isolated.

^1^H NMR (400 MHz, C_6_D_6_, 298 K) δ_H_, ppm: 7.63 (1H, m, Ar*H*), 7.46 (4H, m, Ar*H*), 7.34 (3H, m, Ar*H*), 7.28–7.20 (6H, m, Ar*H*), 7.13–7.02 (6H, m, Ar*H*), 6.97 (1H, m, Ar*H*), 6.92–6.85 (1H, m, Ar*H*), 6.66 (1H, m, Ar*H*), 5.70 (1H, q, *J* = 7.1 Hz, OC*H*), 4.53 (1H, d, *J* = 12.2 Hz, NC*H*_2_Ph), 4.11 (1H, d, *J* = 12.4 Hz, NC*H*_2_Ph), 3.92 (1H, dq, *J* = 10.5, 7.2 Hz, OC*H*_2_CH_3_), 3.64 (1H, dq, *J* = 10.5, 7.1 Hz, OC*H*_2_CH_3_), 2.88 (1H, d, *J* = 12.2 Hz, NC*H*_2_Ph), 2.77 (2H, d + m, *J* = 12.4 Hz, 1 x NC*H*_2_Ph and 1 × NC*H*_2_), 2.52 (1H, m, NC*H*_2_), 2.02 (1H, m, NC*H*_2_), 1.99 (3H, s, C*H*_3_), 1.96 (3H, s, C*H*_3_), 1.85 (3H, s, C*H*_3_), 1.79 (3H, s, C*H*_3_), 1.75 (3H, s, C*H*_3_), 1.68 (3H, br s, NC*H*_3_), 1.61 (3H, s, C*H*_3_), 1.56 (3H, s, C*H*_3_), 1.54 (3H, d, *J* = 7.1 Hz, OCHC*H*_3_), 1.52 (3H, s, C*H*_3_), 1.26 (3H, br s, NC*H*_3_), 1.02 (1H, m, NC*H*_2_), 0.76 (3H, t, *J* = 7.2 Hz, OCH_2_C*H*_3_);

^13^C NMR (100 MHz, C_6_D_6_, 298 K) δ_C_, ppm: 190.6 (*C*=O), 164.0 (d, ^2^ *J*_C–Y_ = 2.9 Hz, Ar*C*O), 163.9 (d, ^2^ *J*_C–Y_ = 2.2 Hz, Ar*C*O), 153.6 (Ar*C*), 153.23 (Ar*C*), 153.19 (Ar*C*), 152.8 (Ar*C*), 136.3 (Ar*C*), 135.3 (Ar*C*), 134.9 (Ar*C*), 134.4 (Ar*C*), 130.56 (Ar*C*H), 130.5 (br, Ar*C*H), 129.3 (Ar*C*H), 129.2 (br, Ar*C*H), 129.1 (Ar*C*H), 128.5 (Ar*C*H), 128.4 (Ar*C*H), 128.34 (Ar*C*H), 128.32 (Ar*C*H), 128.1 (Ar*C*H), 127.8 (Ar*C*H), 127.7 (Ar*C*H), 127.57 (Ar*C*H), 127.54 (Ar*C*H), 126.7 (br, Ar*C*H), 126.7 (Ar*C*), 126.0 (Ar*C*H), 125.9 (Ar*C*H), 125.5 (Ar*C*H), 125.4 (br, Ar*C*H), 124.5 (Ar*C*), 73.5 (O*C*H), 68.3 (O*C*H_2_ THF), 66.2 (N*C*H_2_Ph), 65.7 (N*C*H_2_Ph), 63.6 (O*C*H_2_), 59.7 (N*C*H_2_), 48.4 (N*C*H_2_), 47.3 (N*C*H_3_), 44.9 (*C*(CH_3_)_2_Ph), 44.4 (N*C*H_3_), 43.8 (*C*(CH_3_)_2_Ph), 43.1 (*C*(CH_3_)_2_Ph), 42.7 (*C*(CH_3_)_2_Ph), 34.3 (C­(*C*H_3_)_2_Ph), 32.2 (C­(*C*H_3_)_2_Ph), 32.1 (C­(*C*H_3_)_2_Ph), 31.72 (C­(*C*H_3_)_2_Ph), 31.66 (C­(*C*H_3_)_2_Ph), 29.9 (C­(*C*H_3_)_2_Ph), 28.8 (C­(*C*H_3_)_2_Ph), 26.2 (C­(*C*H_3_)_2_Ph), 26.1 (C*H*_2_ THF), 25.5 (OCH*C*H_3_), 14.3 (OCH_2_ *C*H_3_).

### NMR Scale Reaction of **1** with (*R*)-methyl-3-hydroxybutyrate

To a solution of **1** (0.043 g, 0.04 mmol) in C_6_D_6_ (0.5 mL) in a small vial was added a solution of (*R*)-methyl-3-hydroxybutyrate (0.005 g, 0.04 mmol) in C_6_D_6_ (0.25 mL). The resulting solution was gently shaken to mix, transferred to a J-Young NMR tube and analyzed by NMR spectroscopy within 30 min of sample preparation. The product was not isolated.

## Supplementary Material



## Footnotes

1 A potential explanation for the similarity in *M*_n_ between polymers in entries 2/3 may relate to incomplete initiation of polymerization at high catalyst loading.

## Notes

Full experimental details and characterization data for all polymers (PDF)
