# Supplementary material for: Access to Microstructurally Complex Block Copolymers via Switchable Ring-Opening Polymerization of Cyclic Ester Mixtures
Source: Macromolecules. 2025 Jul 2;58(14):7026–36. doi: 10.1021/acs.macromol.5c00822 (PMC12288056; doi:10.1021/acs.macromol.5c00822)
Supplement: Supplementary file 1 [file ma5c00822_si_001.pdf]

Access to Microstructurally Complex Block-Copolymers via Switchable Ring-Opening Polymerization of Cyclic Ester Mixtures

*David J. E. Seed, Amelia B. Milner, George R. Walker, Rachel H. Platel**
Department of Chemistry, Lancaster University, Lancaster, LA1 4YB, United Kingdom.

Supporting Information

Contents

1. NMR Spectra of 1 – 4 6

Figure S1: ^{1}H NMR Spectrum of **1** 6

Figure S2: ^{13}C{^{1}H} NMR Spectrum of **1** 6

Figure S3: ^{1}H NMR Spectrum of **2** 7

Figure S4: ^{13}C{^{1}H} NMR Spectrum of **2** 7

Figure S5: ^{1}H NMR Spectrum of **3** 8

Figure S6: ^{13}C{^{1}H} NMR Spectrum of **3** 9

Figure S7: ^{1}H NMR Spectrum of **4** 9

Figure S8: ^{13}C{^{1}H} NMR Spectrum of **4** 10

2. Determination of Polymer Compositions 10

2.1 Determination of overall polymer composition from 1H NMR spectrum 10

Figure S9: ^{1}H NMR Spectrum of a typical copolymer 10

2.2 Determination of Composition of each polymer block using ^{1}H NMR spectrum 11

Figure S10: Block Composition using ^{1}H NMR 11

2.3 Determination of block microstructure using ^{13}C NMR spectra 12

Figure 3 (From main manuscript, Polymer from Table 1, entry 1) 12

Detail from Figure 3 (Table 1 entry 1) 13

3. Representative NMR spectra of copolymers 14

Figure S11: ^{1}H NMR Spectrum of Polymer in Table 1, Entry 1 14

Figure S12: ^{13}C NMR Spectrum of Polymer in Table 1, Entry 1 14

Figure S13: ^{1}H NMR Spectrum of Polymer in Table 1, Entry 2 15

Figure S14: ^{13}C NMR Spectrum of Polymer in Table 1, Entry 2 15

Figure S15: ^{1}H NMR Spectrum of Polymer in Table 1, Entry 3 16

Figure S16: ^{13}C NMR Spectrum of Polymer in Table 1, Entry 3 16

Figure S17: ^{1}H NMR Spectrum of Polymer in Table 1, Entry 4 17

Figure S18: ^{13}C NMR Spectrum of Polymer in Table 1, Entry 4 17

Figure S19: ^{1}H NMR Spectrum of Polymer in Table 1, Entry 5 18

Figure S20: ^{13}C NMR Spectrum of Polymer in Table 1, Entry 5 18

Figure S21: ^{1}H NMR Spectrum of Polymer in Table 1, Entry 6 19

Figure S22: ^{13}C NMR Spectrum of Polymer in Table 1, Entry 6 19

Figure S23: ^{1}H NMR Spectrum of Polymer in Table 1, Entry 7 20

Figure S24: ^{13}C NMR Spectrum of Polymer in Table 1, Entry 7 20

Figure S25: ^{1}H NMR Spectrum of Polymer in Table 1, Entry 8 21

Figure S26: ^{13}C NMR Spectrum of Polymer in Table 1, Entry 8 21

Figure S27: ^{1}H NMR Spectrum of Polymer in Table 1, Entry 9 22

Figure S28: ^{13}C NMR Spectrum of Polymer in Table 1, Entry 9 22

Figure S29: ^{1}H NMR Spectrum of Polymer in Table 1, Entry 10 23

Figure S30: ^{13}C NMR Spectrum of Polymer in Table 1, Entry 10 23

4. Representative GPC traces of copolymers 24

Figure S31 GPC trace from polymer in Table 1, Entry 1.... 24

Figure S32: GPC trace from polymer in Table 1, Entry 2.... 24

Figure S33: GPC trace from polymer in Table 1, Entry 3.... 25

Figure S34: GPC trace from polymer in Table 1, Entry 4.... 25

Figure S35: GPC trace from polymer in Table 1, Entry 5.... 26

Figure S36: GPC trace from polymer in Table 1, Entry 6.... 26

Figure S37: GPC trace from polymer in Table 1, Entry 7.... 27

Figure S38: GPC trace from polymer in Table 1, Entry 8.... 27

Figure S39: GPC trace from polymer in Table 1, Entry 9 28

Figure S40: GPC trace from polymer in Table 1, Entry 10 28

5. Polymerization Reaction and Copolymer Composition Profiles with 2 – 4 ... 28

Table S1: One-pot ROP of *rac*-LA, *rac*-β-BL and ε-CL catalyzed by **2** – **4**. .... **Error! Bookmark not defined.**

Figure S41: Plot of Monomer Conversion using $Y(N(SiHMe_2)_2)_3(THF)_2$ 29

Figure S42: Plot of Monomer Conversion for Table 2, Entry 1 29

Figure S43: Plot of overall polymer composition with monomer conversion, Table 2, Entry 1 30

Figure S44: Plot of Monomer Conversion for Table 2, Entry 2 30

Figure S45: Plot of overall polymer composition with monomer conversion, Table 2, Entry 2 31

Figure S46: Plot of Monomer Conversion for Table 2, Entry 3 31

6. DSC Data for Polymers 32

Table S2: Thermal properties of $(AB)_x(BC)_y$ block copolymers[a] 32

7. Additional Experiments 32

Figure S47: Plot of Monomer Conversion for Table 1, Entry 8 32

Figure S48: Plot of Monomer Conversion for Table 1, Entry 9 33

Figure S49: Plot of Monomer Conversion for Table 1, Entry 9 34

Figure S50: BASHD (band selective homonuclear decoupled) ^{1}H NMR spectrum of PLA 34

Figure S51: Semi-logarithmic plot of the polymerization of *rac*-LA in the presence of ε-CL and *rac*-β-BL 35

Figure S52: Semi-logarithmic plot of the polymerization of *rac*-LA in the presence of ε-CL 35

Figure S53: Plot to show conversion over time in the copolymerization of *rac*-β-BL and ε-CL by **1.** 35

Figure S54: Semilogarithmic plot for ε-CL polymerization between 4min and 20 min in the copolymerization of *rac*-β-BL and ε-CL by **1.** 36

Figure S55: Semilogarithmic plot for *rac*-β-BL polymerization between 4 min and 17 min in the copolymerization of *rac*-β-BL and ε-CL by **1.** 36

Figure S56: ^{13}C NMR spectra of aliquots removed from a copolymerization reaction after 2 h (bottom spectrum) and 24 h (top spectrum). 37

Figure S57: ^{13}C NMR spectra of aliquots removed from a copolymerization reaction after 30 min (red trace, bottom), and 2 h (green trace, top). 38

Figure S58: ^{1}H NMR Spectrum obtained after mixing **1** with 1 eq. (*S*)-Ethyl lactate in C_6D_6 39

Figure S59: ^{13}C NMR Spectrum obtained after mixing **1** with 1 eq. (*S*)-Ethyl lactate in C_6D_6 39

Figure S60: ^{1}H NMR Spectrum obtained after mixing **1** with 1 eq. (*R*)-methyl-3-hydroxybutyrate in C_6D_6 40

Figure S61: ^{13}C NMR Spectrum obtained after mixing **1** with 1 eq. (*R*)-methyl-3-hydroxybutyrate in C_6D_6 41

1. NMR Spectra of 1 – 4

Figure S1: ^{1}H NMR Spectrum of **1**

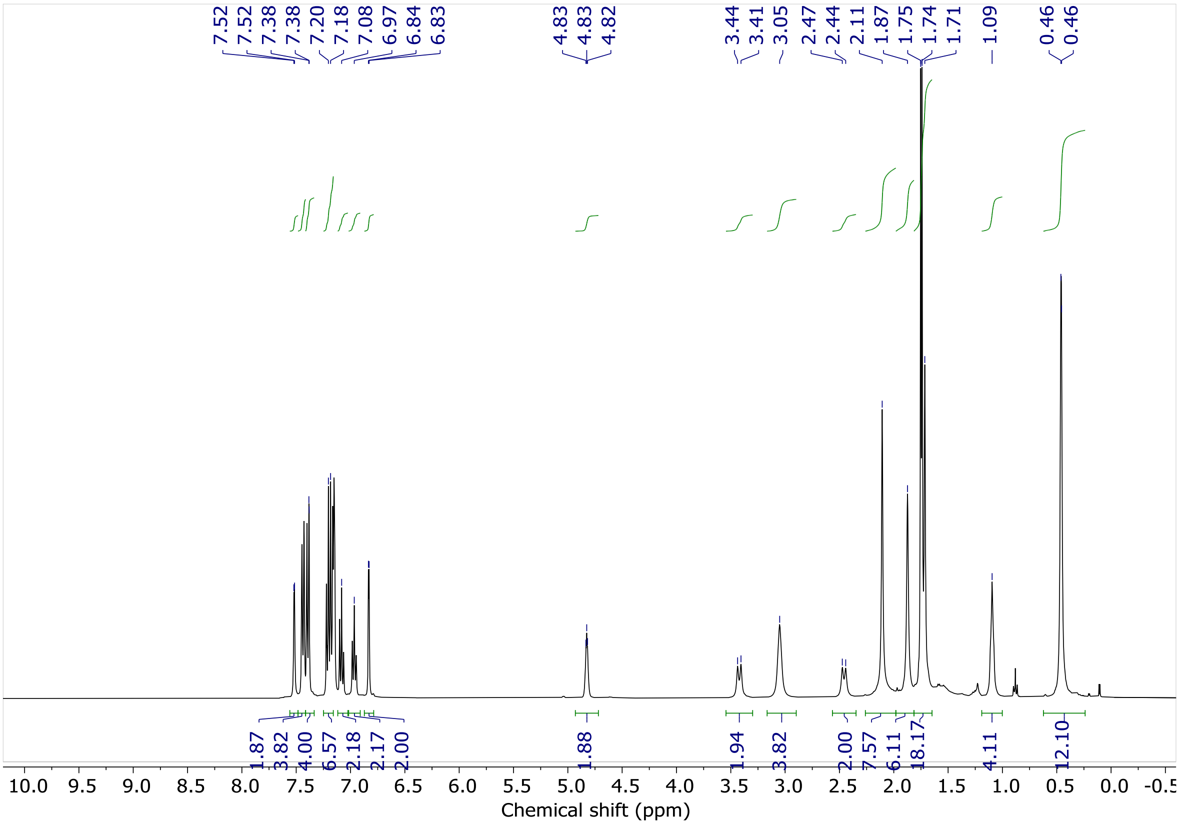

Figure S2: $^{13}C\{^{1}H\}$ NMR Spectrum of **1**

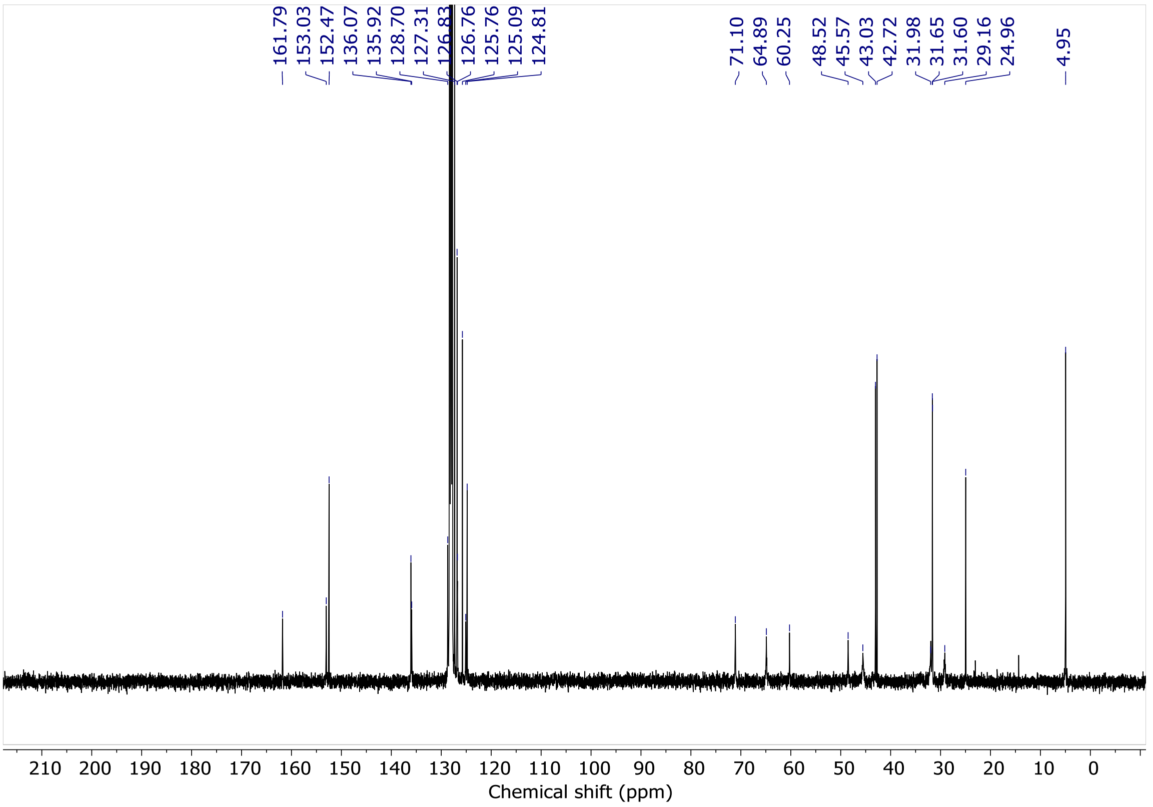

Figure S3: ^{1}H NMR Spectrum of **2**

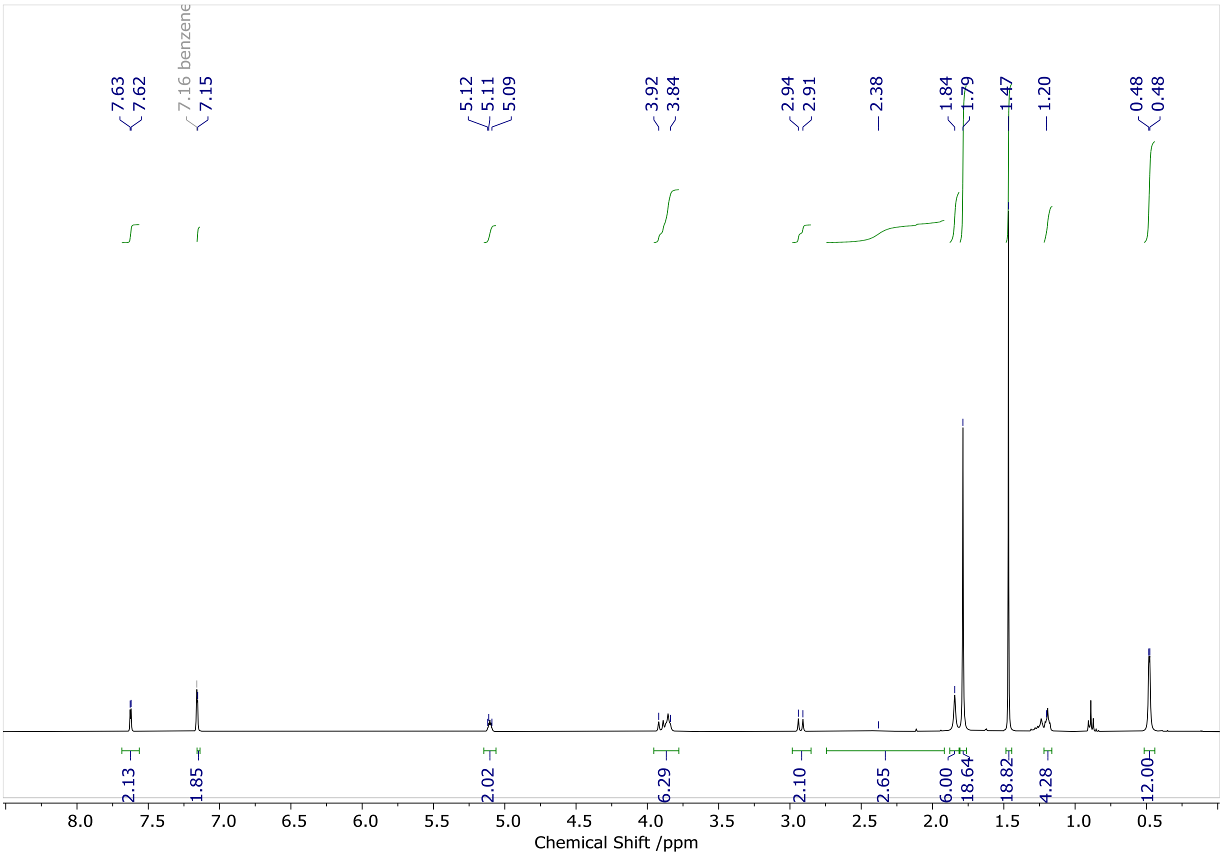

Figure S4: ^{13}C{^{1}H} NMR Spectrum of **2**

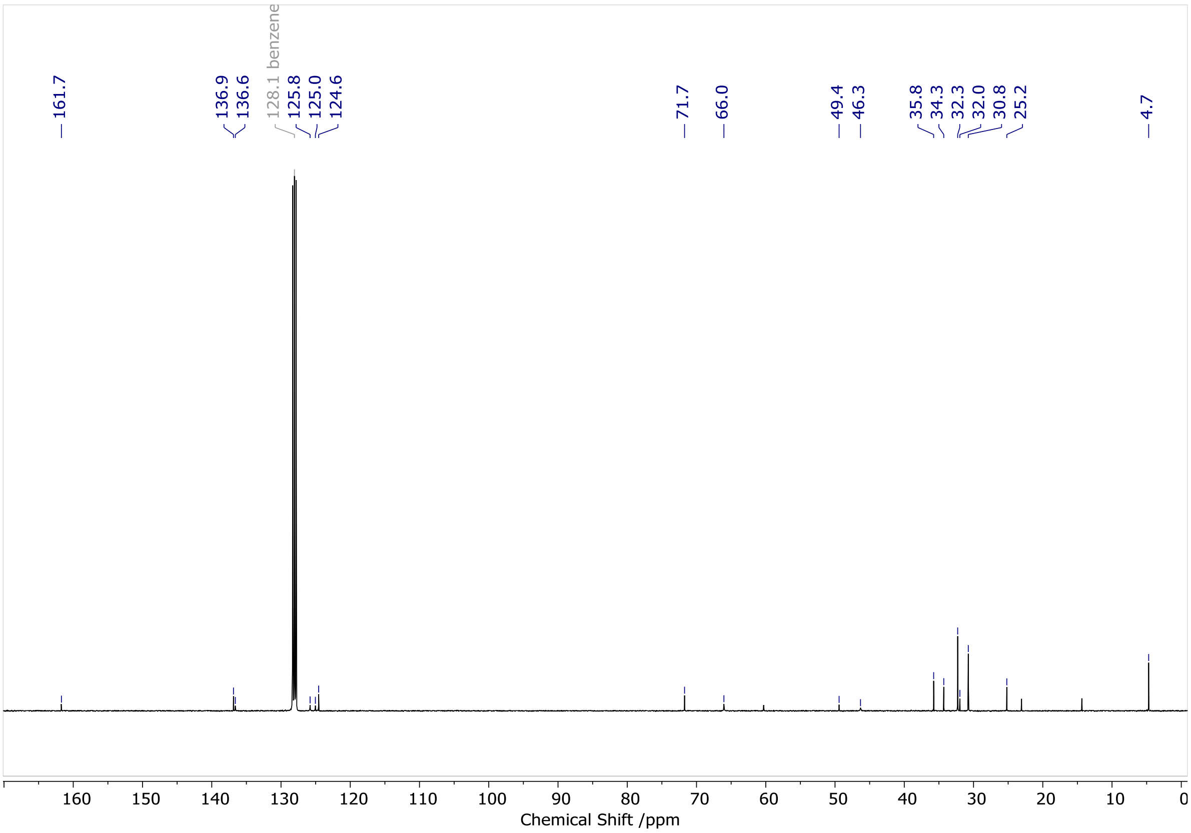

Figure S5: ^{1}H NMR Spectrum of **3**

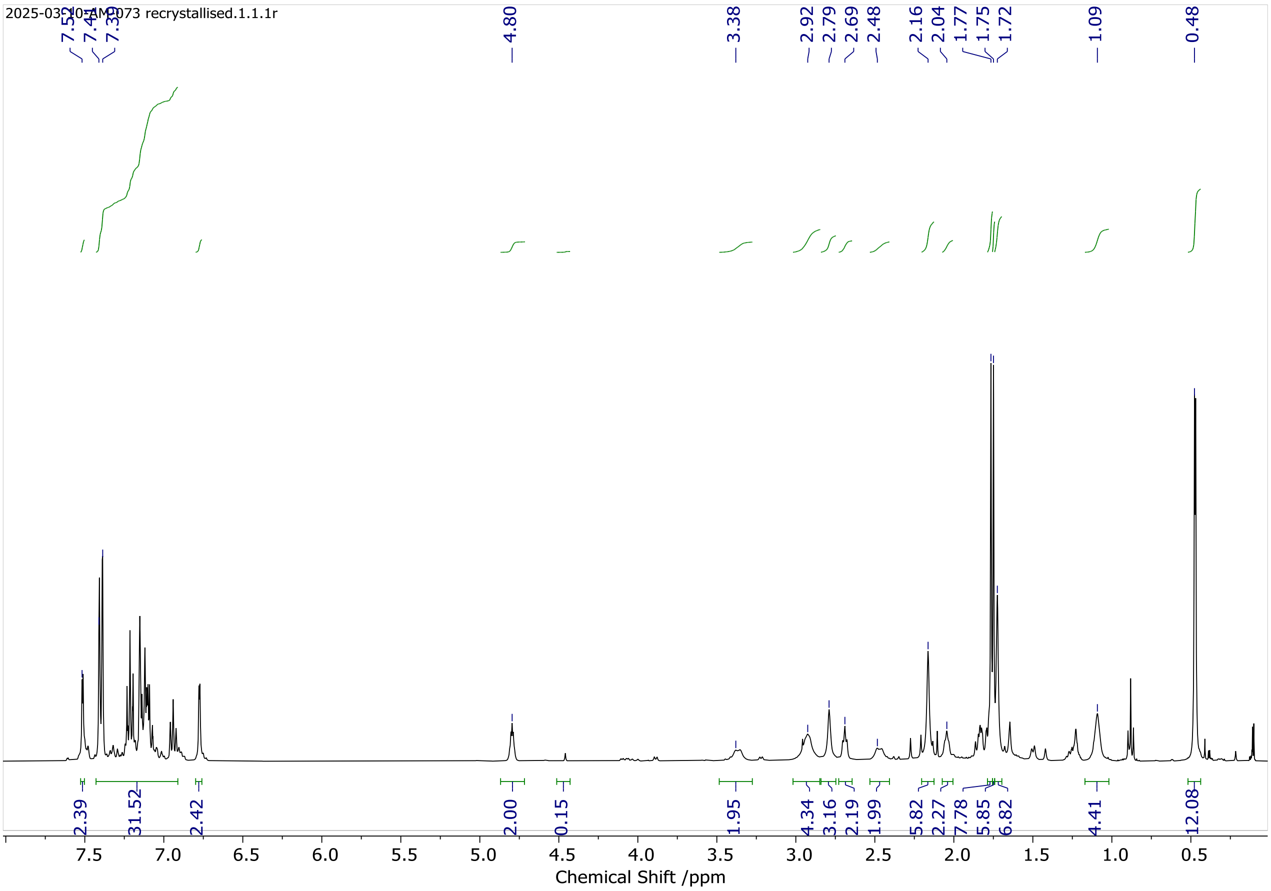

Figure S6: $^{13}C\{^{1}H\}$ NMR Spectrum of **3**

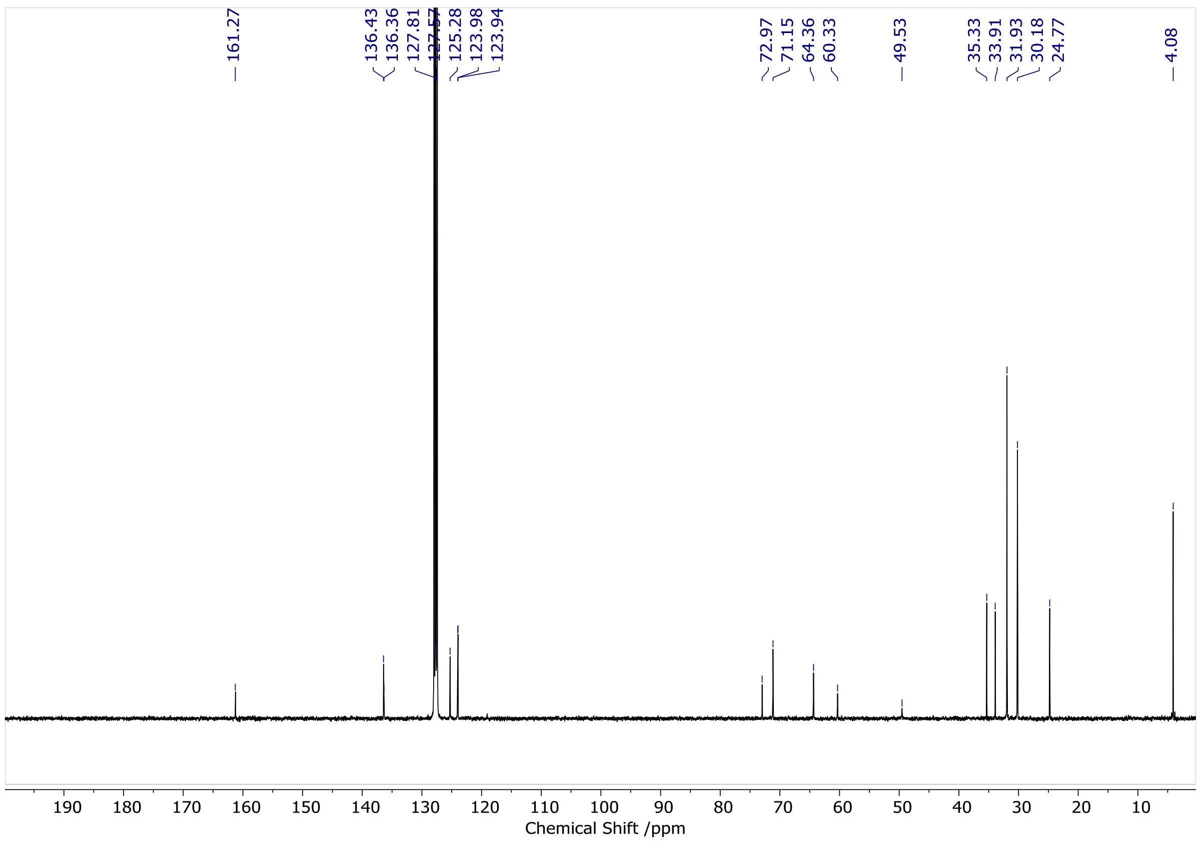

Figure S7: ^{1}H NMR Spectrum of **4**

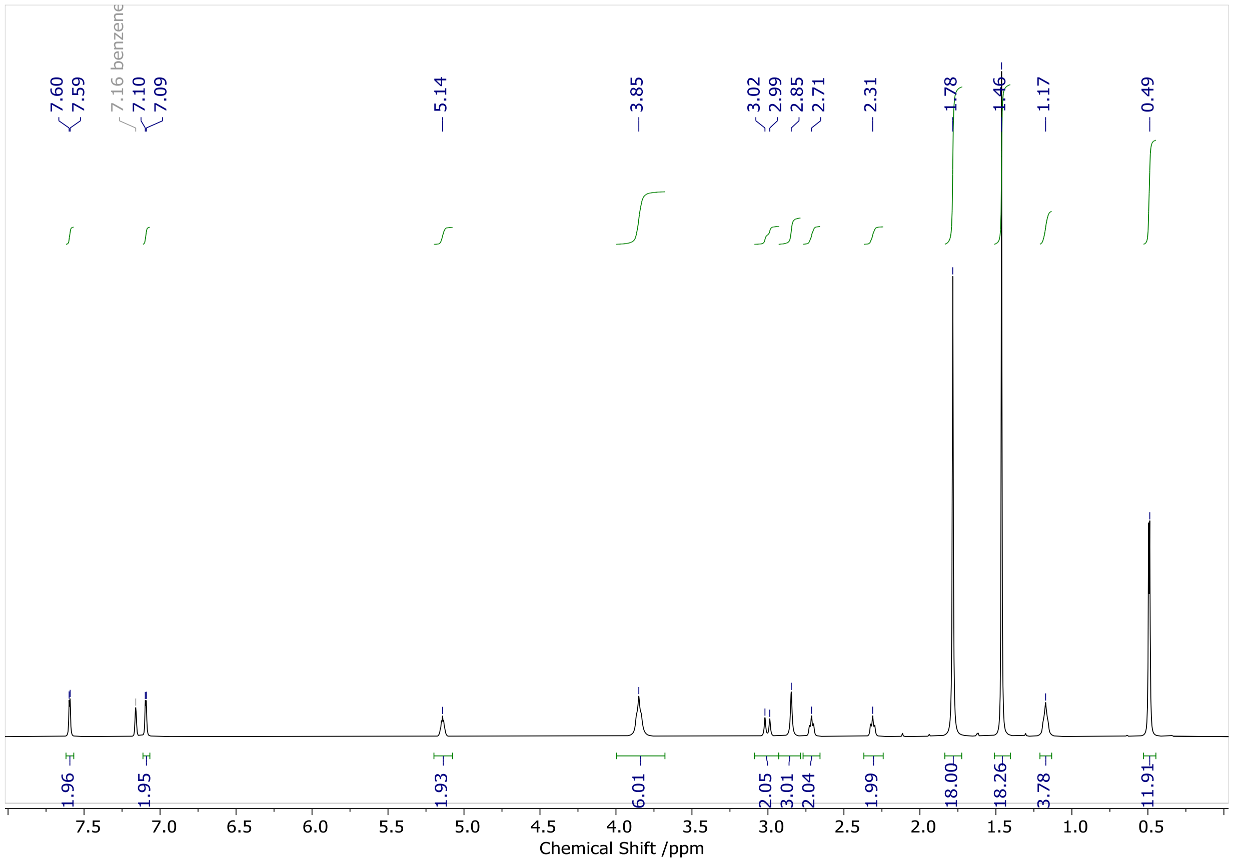

Figure S8:$^{13}C\{^{1}H\}$ NMR Spectrum of **4**

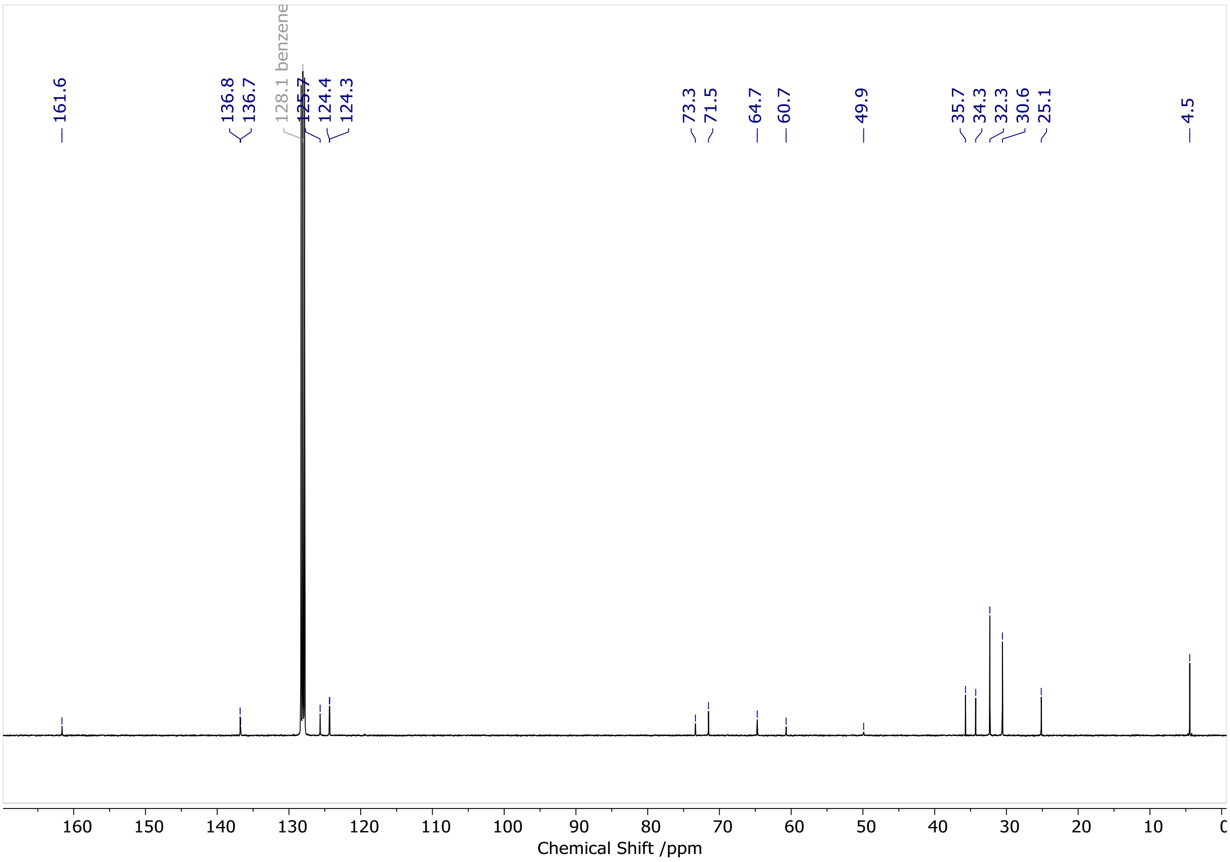

2. Determination of Polymer Compositions

2.1 Determination of overall polymer composition from 1H NMR spectrum

Figure S9:^{1}H NMR Spectrum of a typical copolymer

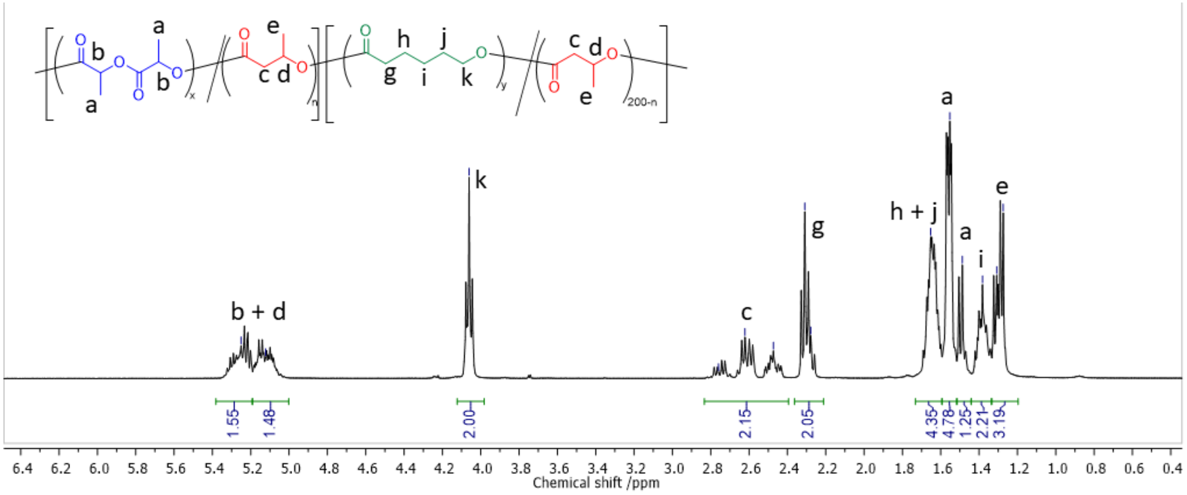

Overall polymer composition was determined using from an NMR spectrum similar to that in Figure S3, using the integrals of protons b and d (2 x CH of LL and 1 x CH of B units), k (1 x CH_2 of C unit), c (1 x CH_2 of B unit).

$Int_B = A_{2.41-2.82}$

$Int_{LL} = A_{5.00-5.35} - 0.5\ A_{2.41-2.82}$

$Int_C = A_{3.98-4.11}$

$F_B = Int_B/(Int_B + Int_{LL} + Int_C)$

$F_{LL} = Int_{LL}/(Int_B + Int_{LL} + Int_C)$

$F_C = Int_C/(Int_B + Int_{LL} + Int_C)$

2.2 Determination of Composition of each polymer block using ^{1}H NMR spectrum

LL and C units are in different polymer blocks, confirmed by the absence of a signal from H_k at 4.12 ppm.[1] The chemical shift of H_c in B units is sensitive to environment. Signals for H_c in BLL lie at 2.75 ppm (1H) and 2.60 ppm (1H); signals for BB and BC lie at 2.60 (1H) and 2.46 ppm (1H).[2]

Figure S10: Block Composition using ^{1}H NMR

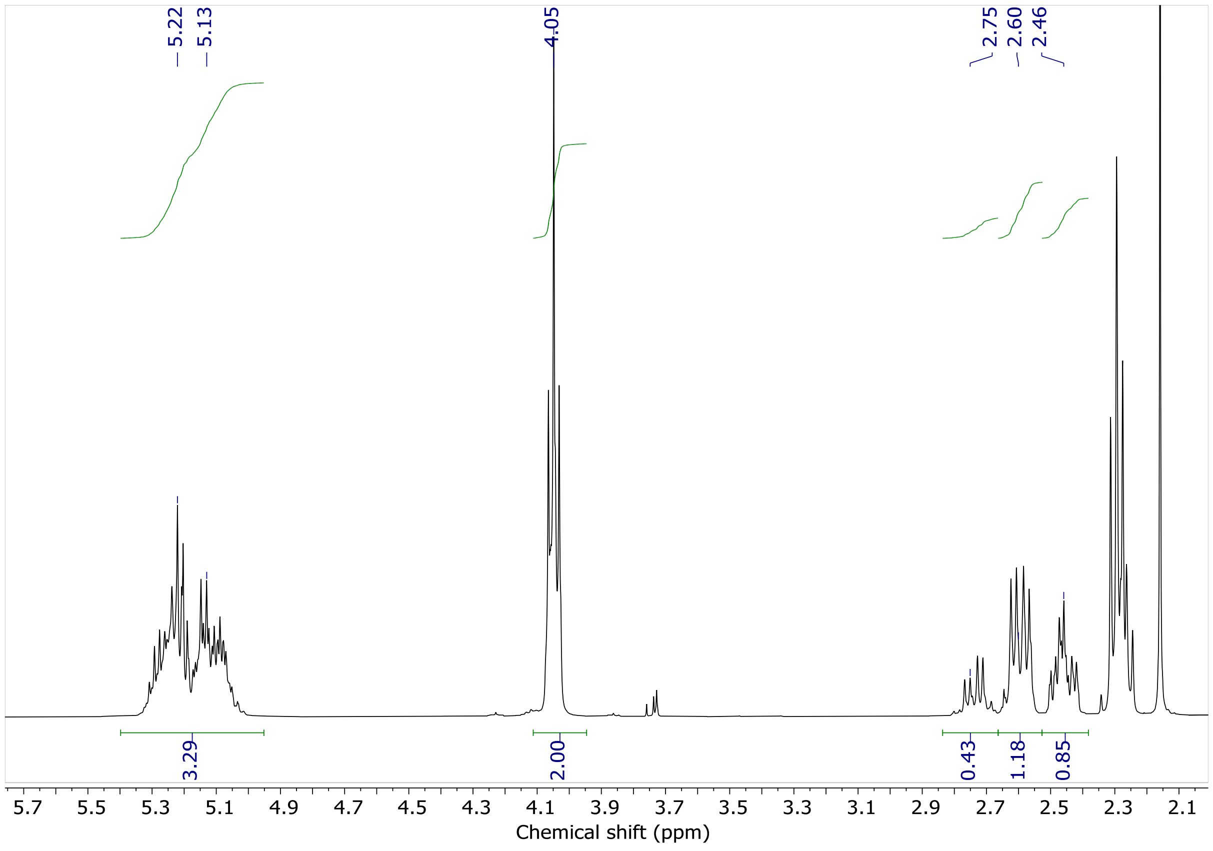

B units in BC block:

$Int_{B(BC)} = 2 \times A_{2.41-2.53} = 2 \times 0.85 = 1.70$

The composition of the BC block is calculated as follows:

$F_B = Int_{B(BC)}/(Int_{B(BC)} + Int_C) = 1.70/(1.70 + 2.00) = 0.46$

$F_C = Int_C/(Int_{B(BC)} + Int_C) = 2.00/(1.70 + 2.00) = 0.54$

B units in LLB block:

$Int_{B(BLL)} = 2 \times A_{2.66-2.82} = 2 \times 0.43 = 0.86$

LL units:

$Int_{LL} = A_{5.00-5.35} - 0.5\ A_{2.41-2.82} = 3.29 - (0.5 \times 2.46) = 2.06$

The composition of the BLL block is calculated as follows:

$F_B = Int_{B(BLL)}/(Int_{B(BLL)} + Int_{LL}) = 0.86/(0.86 + 2.06) = 0.29$

$F_{LL} = Int_{LL}/(Int_{B(BLL)} + Int_{LL}) = 2.06/(0.86 + 2.06) = 0.71$

2.3 Determination of block microstructure using ^{13}C NMR spectra

Figure 3 (From main manuscript, Polymer from Table 1, entry 1)

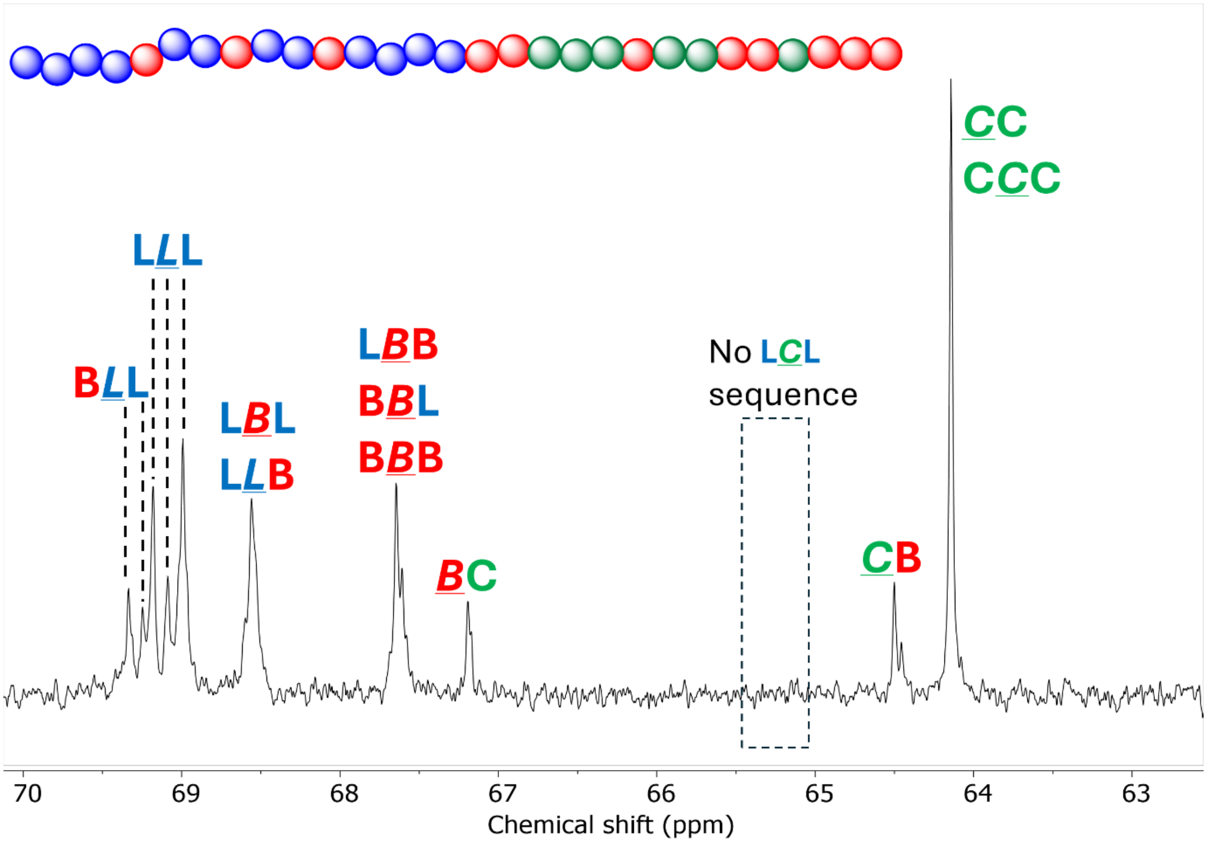

Determination of microstructure of the LL/B Block

Peak assignments from literature.[3]

% BLL sequences = $A_{69.6-69.2}$

% LLL sequences = $A_{69.2-68.9}$

%LLB + %LBL sequences = $A_{68.75-68.45}$

Determination of microstructure of the B/C Block

Peak assignments from literature.[4]

% BB sequences = $A_{67.7-67.5}$ = 31%

% BC sequences = $A_{67.3-67.1}$ = 10%

% CB sequences = $A_{64.6-64.4}$ = 12%

% CC sequences = $A_{64.2-64.0}$ = 47%

Detail from Figure 3 (Table 1 entry 1)

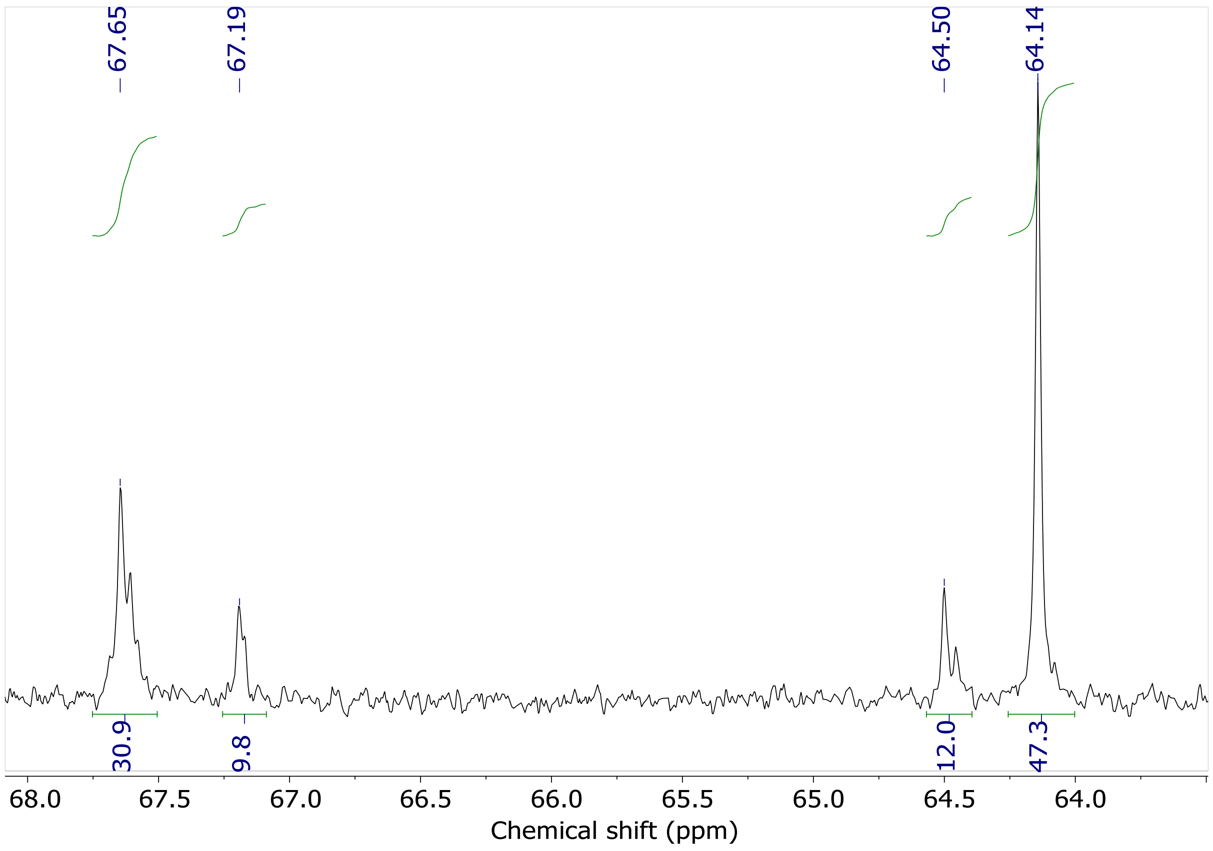

3. Representative NMR spectra of copolymers

Figure S11: ^{1}H NMR Spectrum of Polymer in Table 1, Entry 1

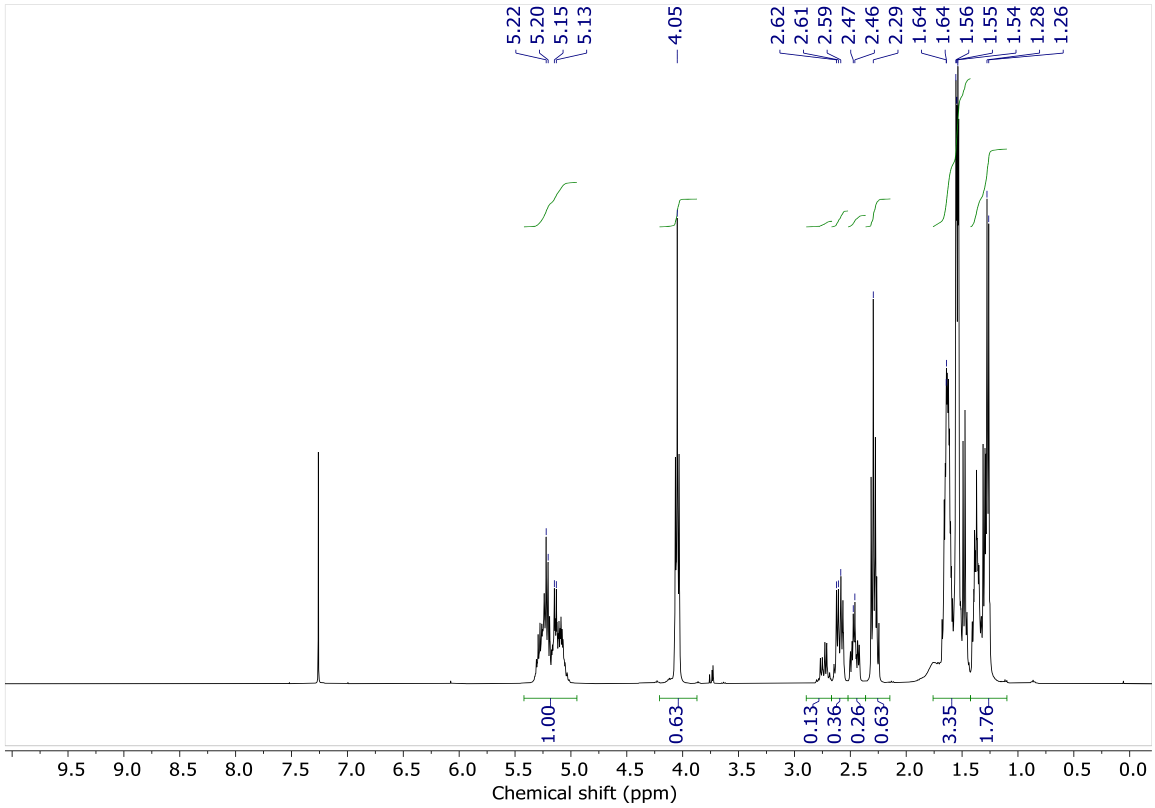

Figure S12: ^{13}C NMR Spectrum of Polymer in Table 1, Entry 1

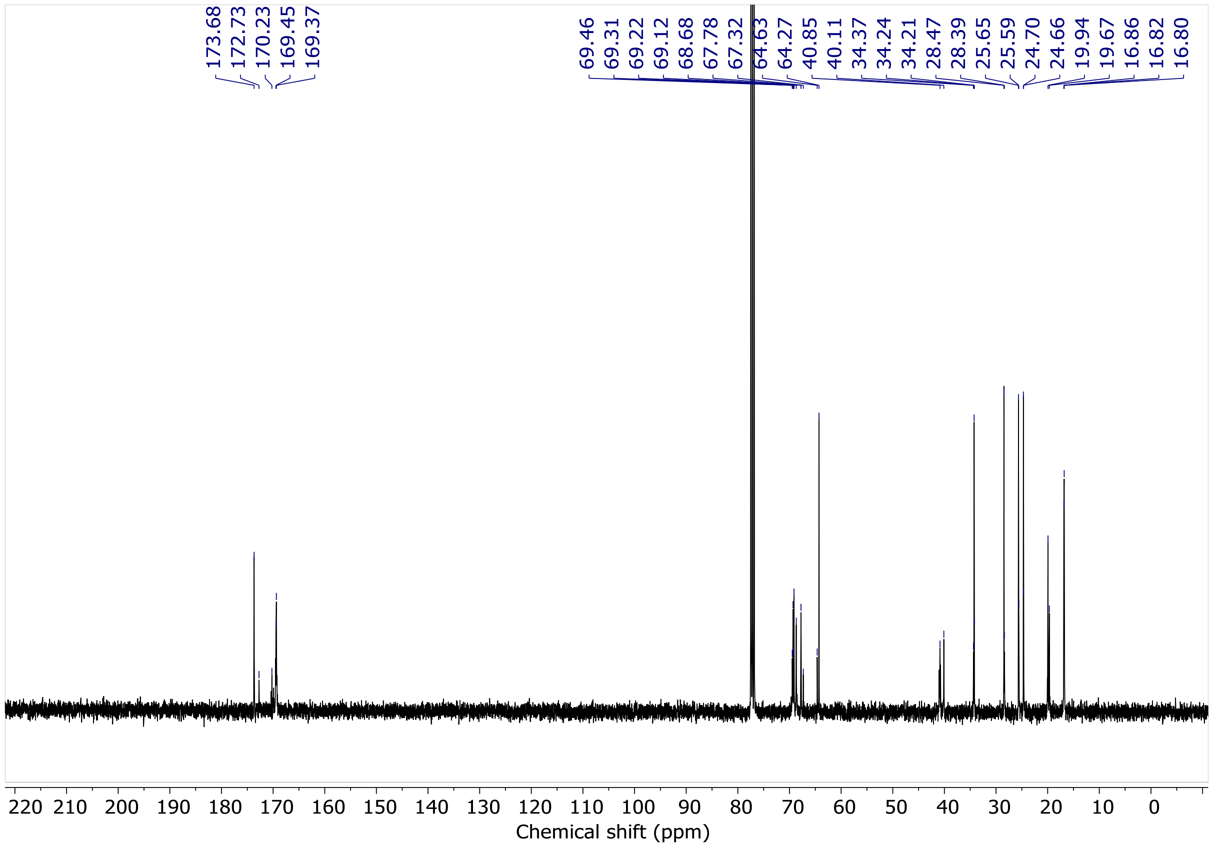

Figure S13: ^{1}H NMR Spectrum of Polymer in Table 1, Entry 2

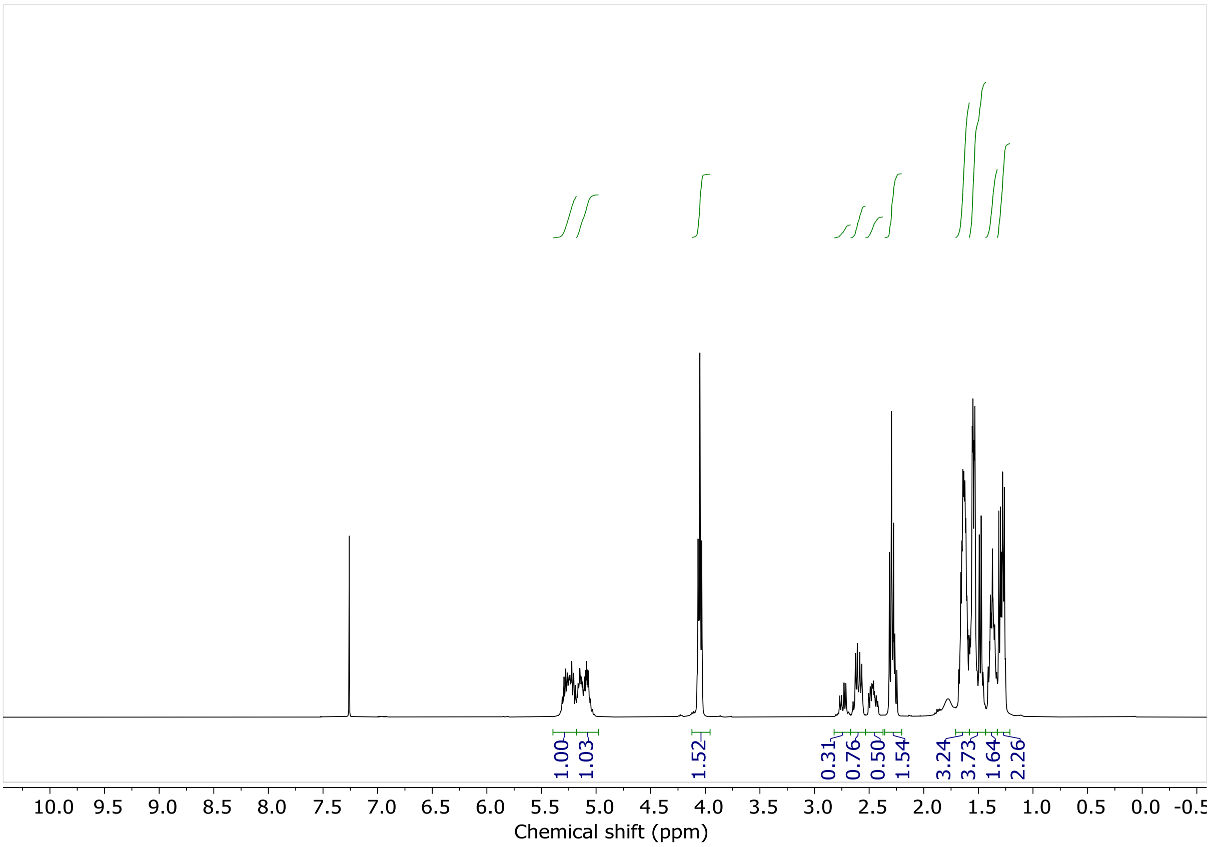

Figure S14: ^{13}C NMR Spectrum of Polymer in Table 1, Entry 2

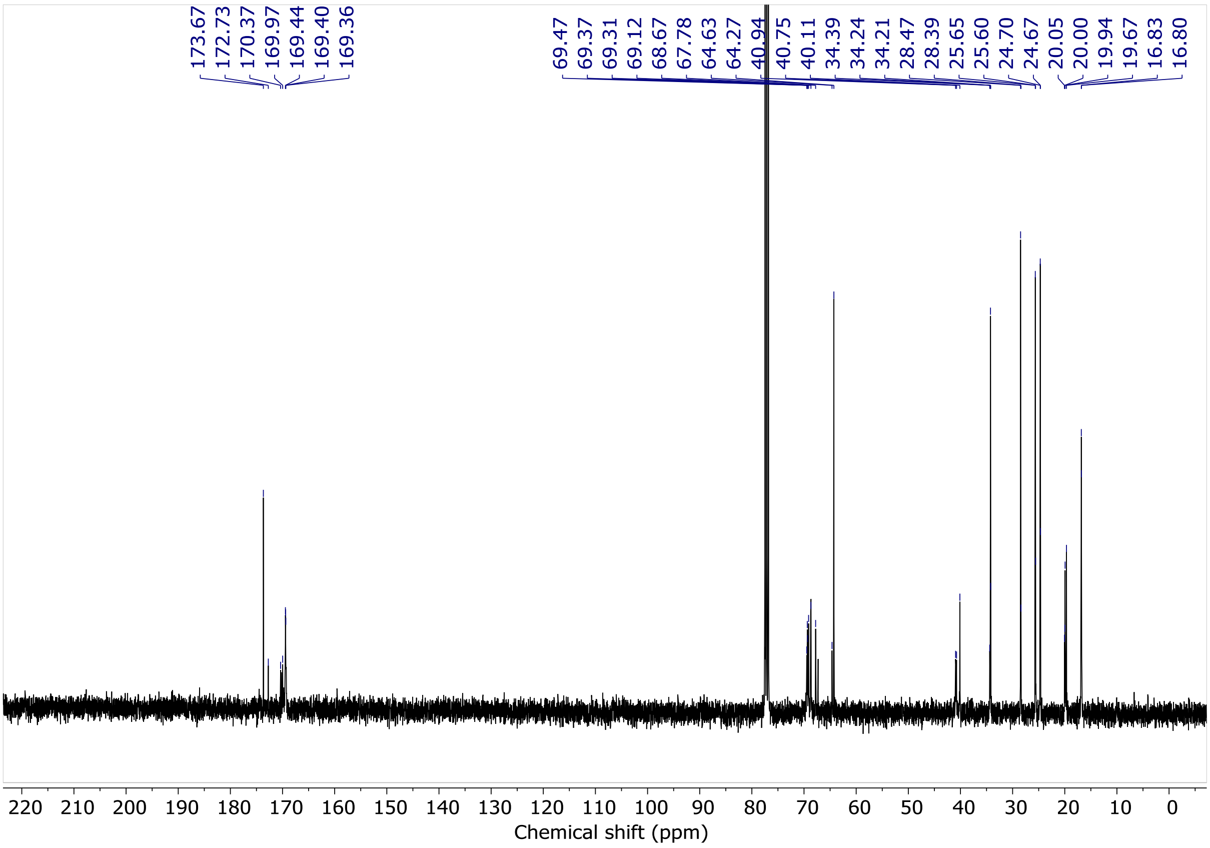

Figure S15: ^{1}H NMR Spectrum of Polymer in Table 1, Entry 3

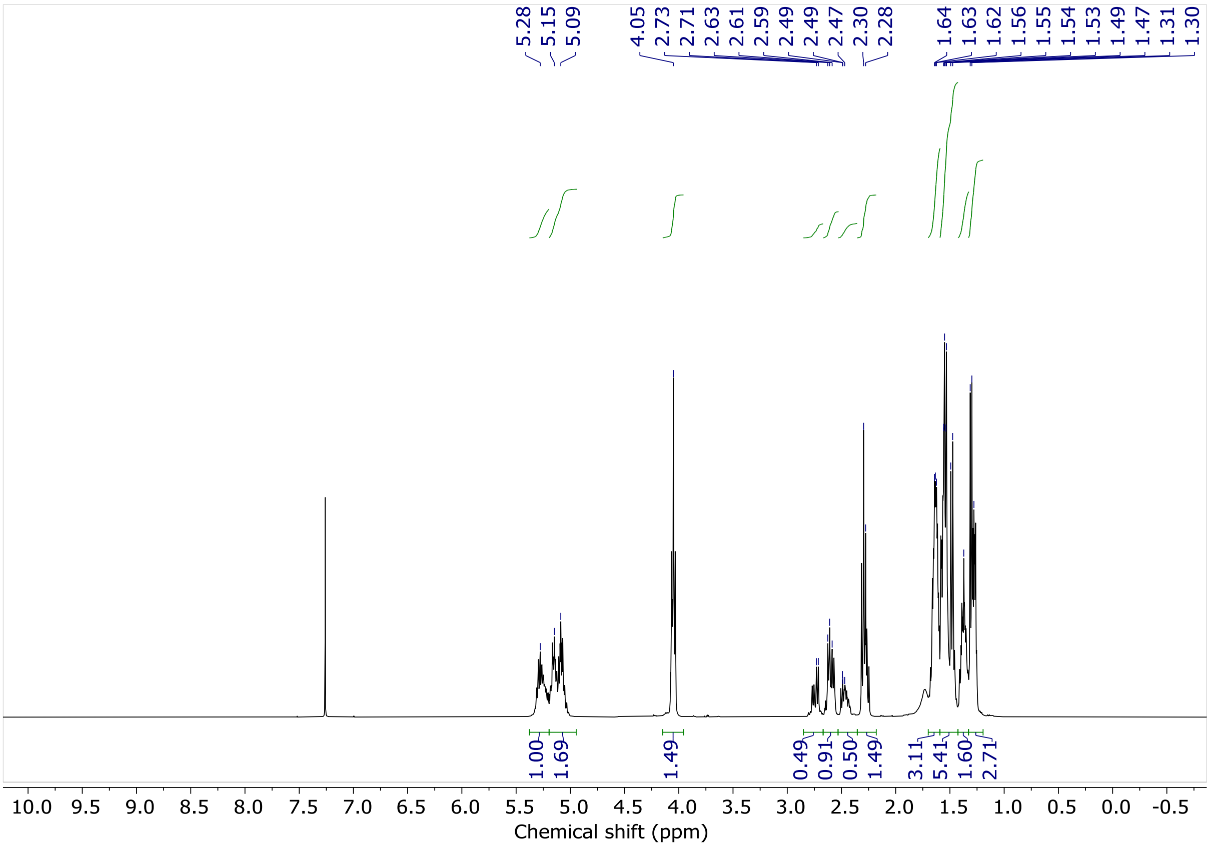

Figure S16: ^{13}C NMR Spectrum of Polymer in Table 1, Entry 3

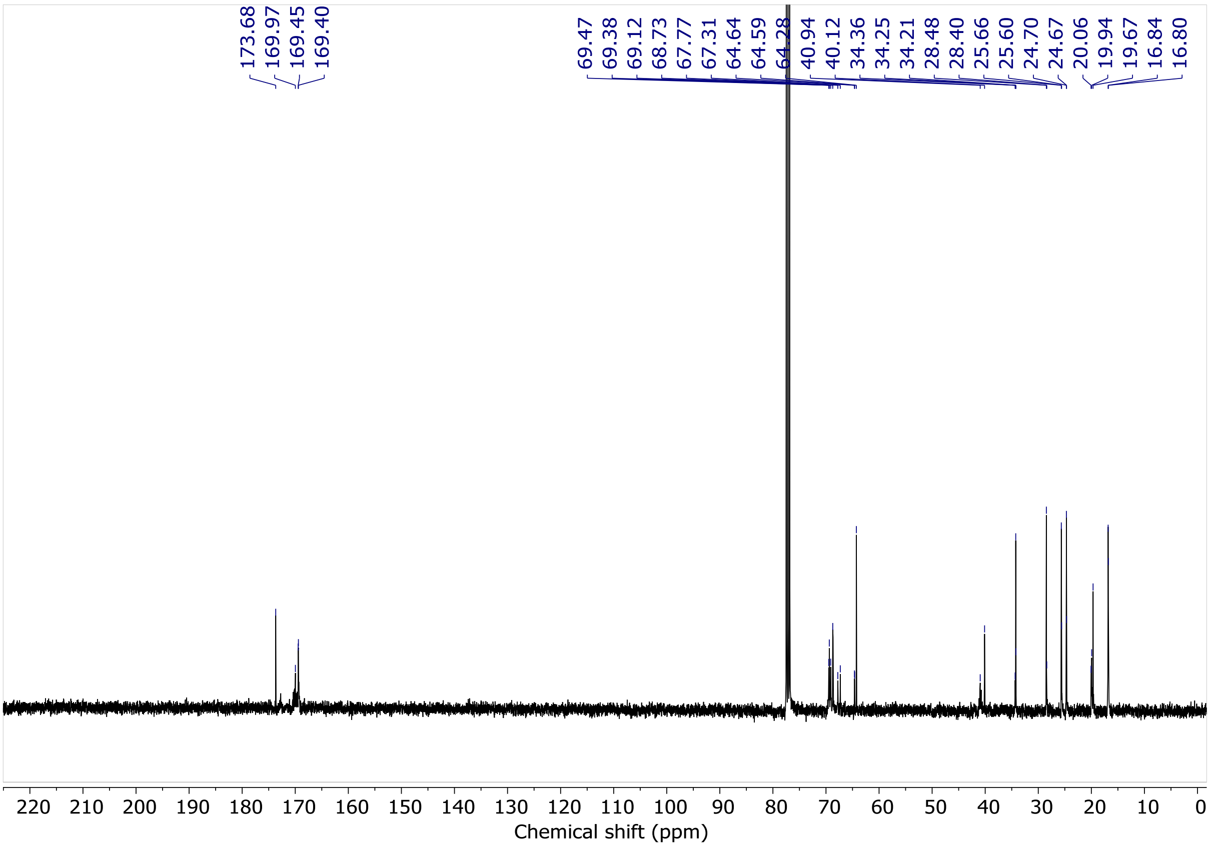

Figure S17: ^{1}H NMR Spectrum of Polymer in Table 1, Entry 4

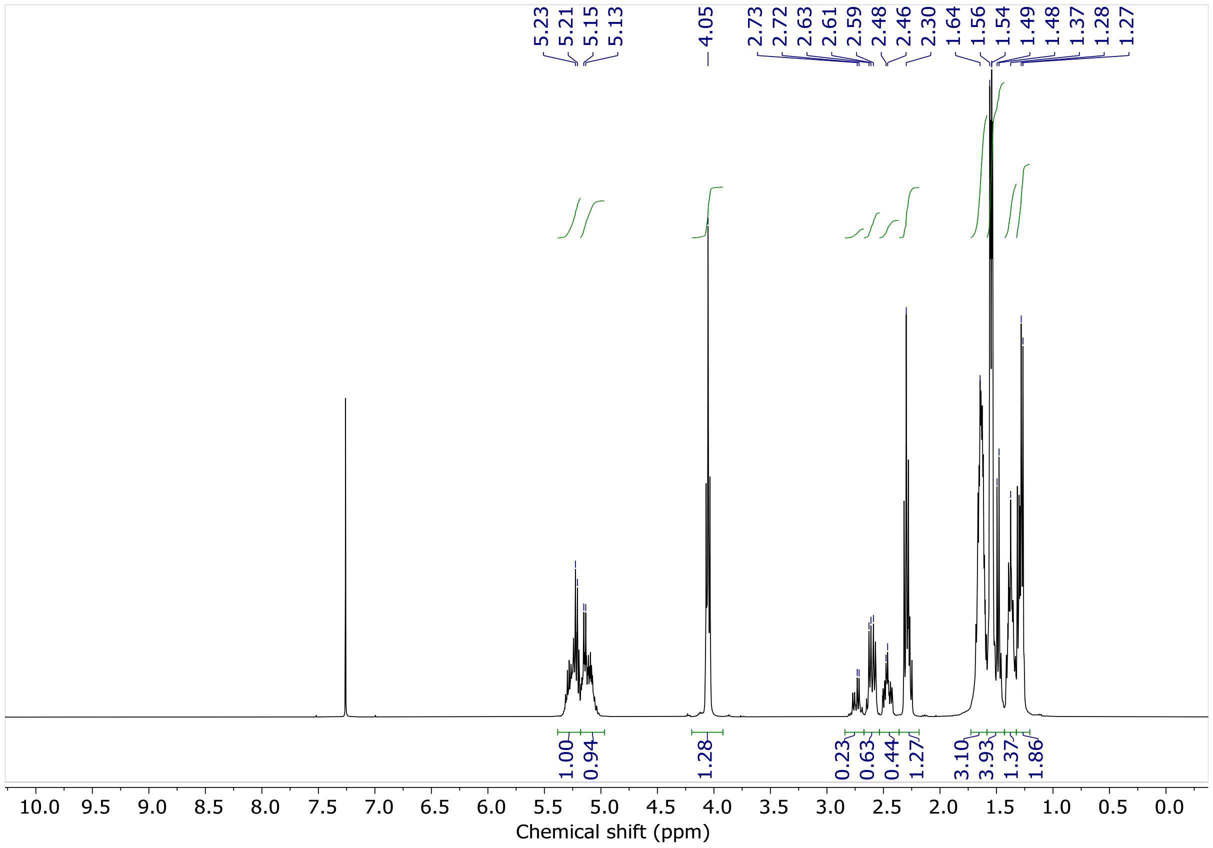

Figure S18: ^{13}C NMR Spectrum of Polymer in Table 1, Entry 4

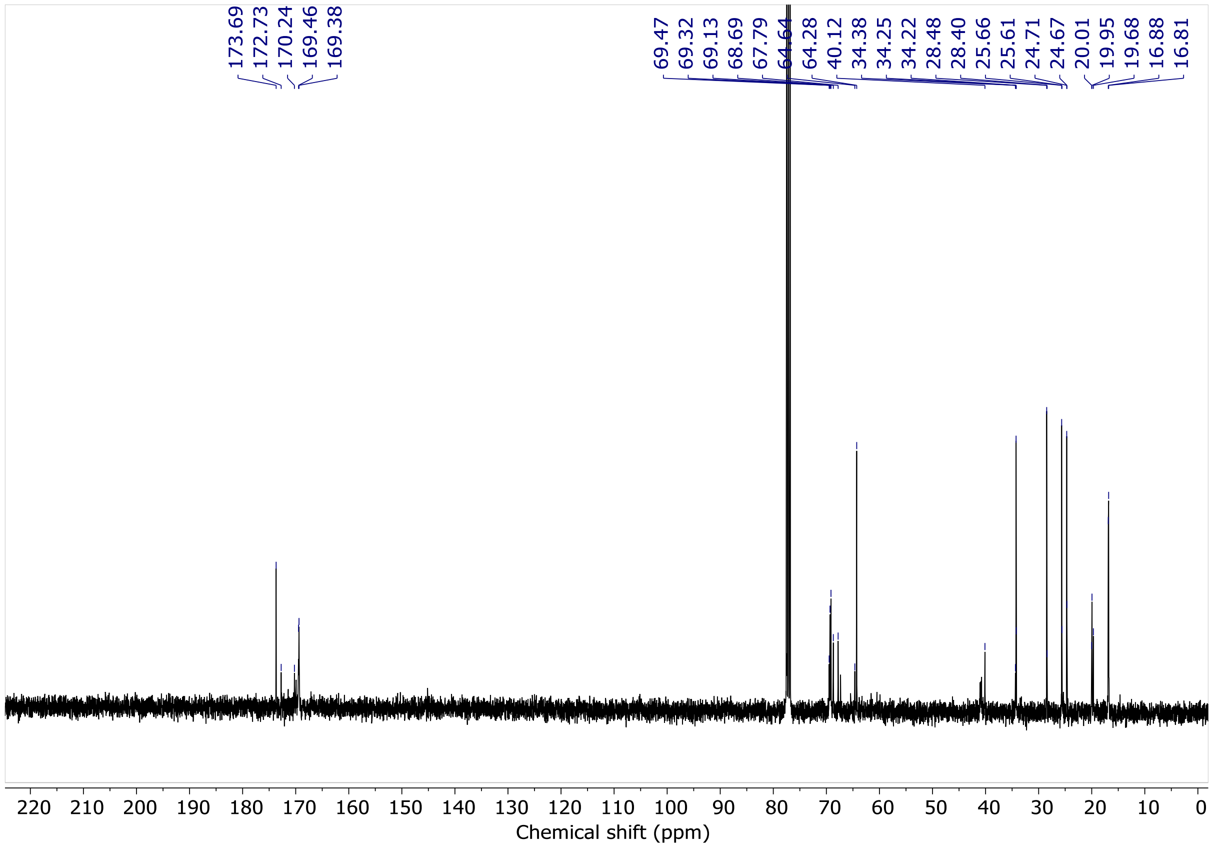

Figure S19: ^{1}H NMR Spectrum of Polymer in Table 1, Entry 5

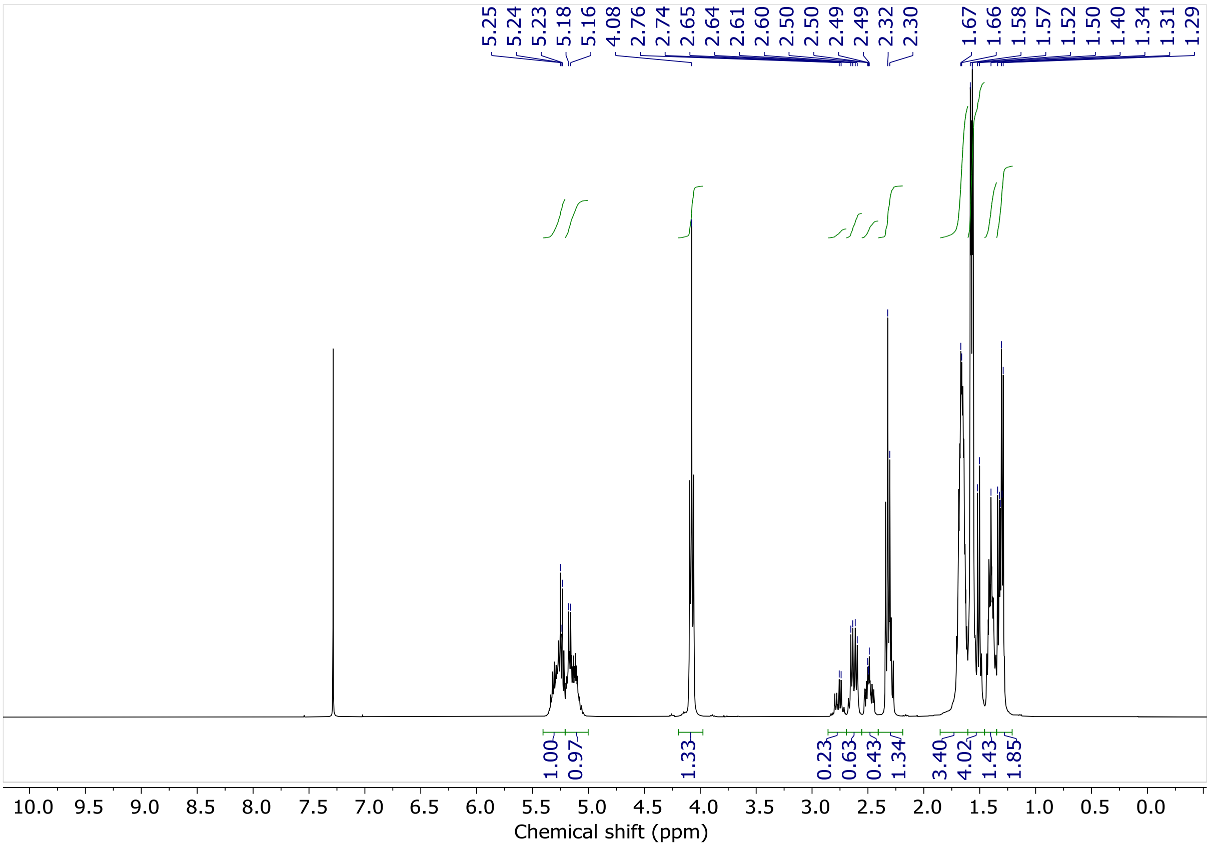

Figure S20: ^{13}C NMR Spectrum of Polymer in Table 1, Entry 5

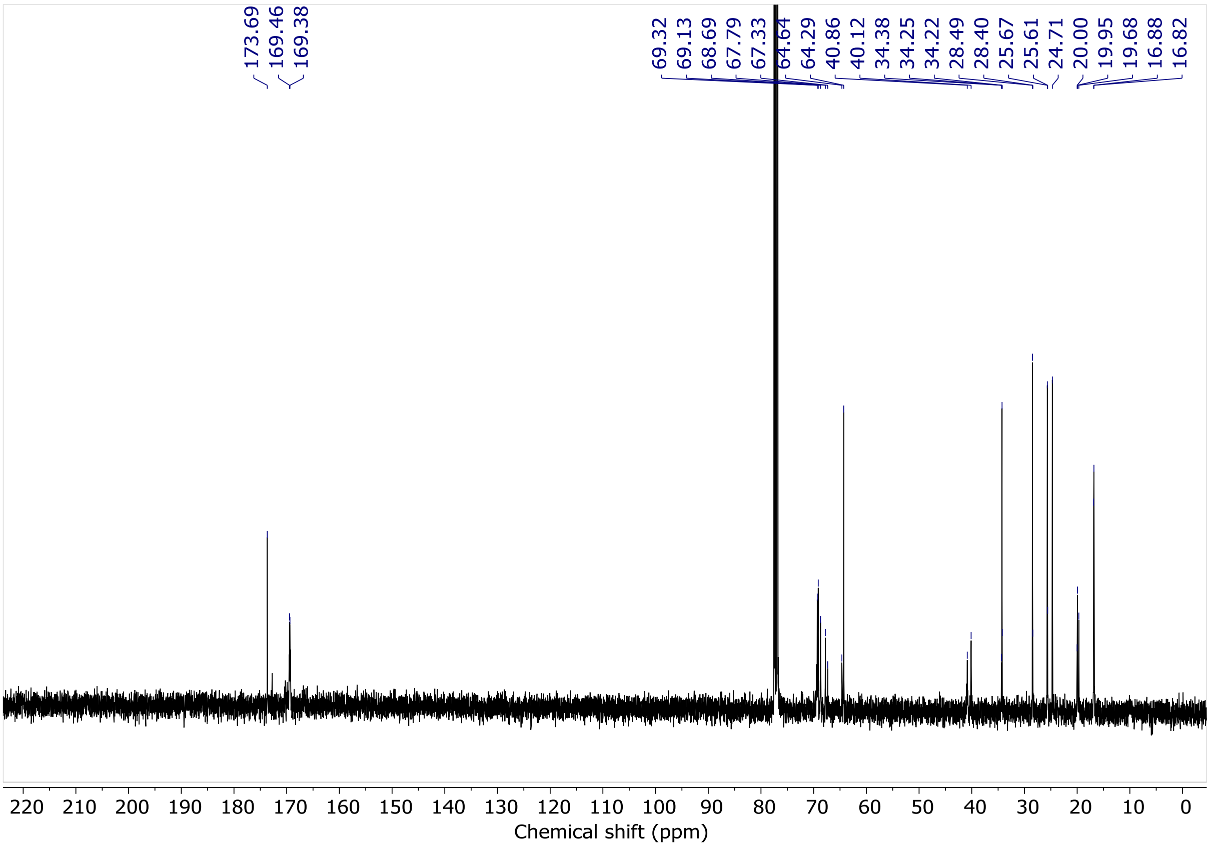

Figure S21: ^{1}H NMR Spectrum of Polymer in Table 1, Entry 6

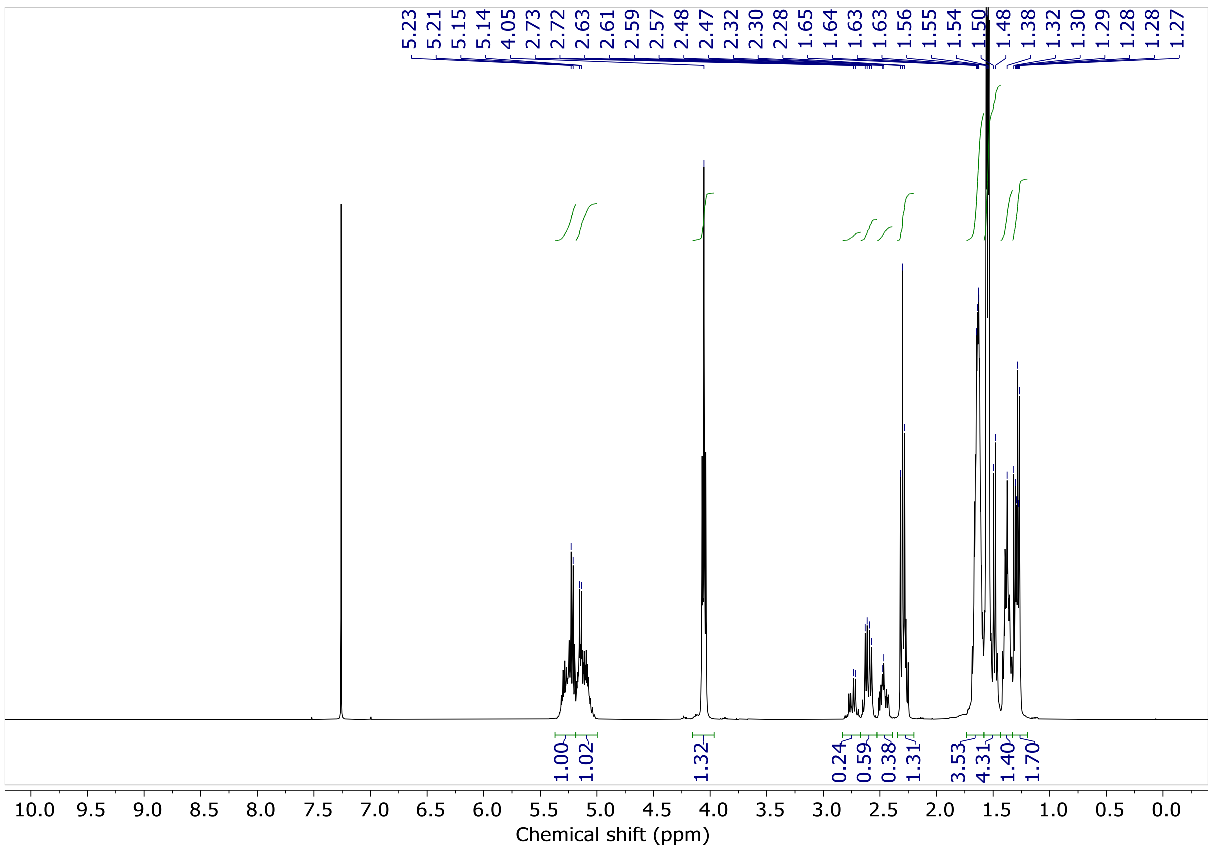

Figure S22: ^{13}C NMR Spectrum of Polymer in Table 1, Entry 6

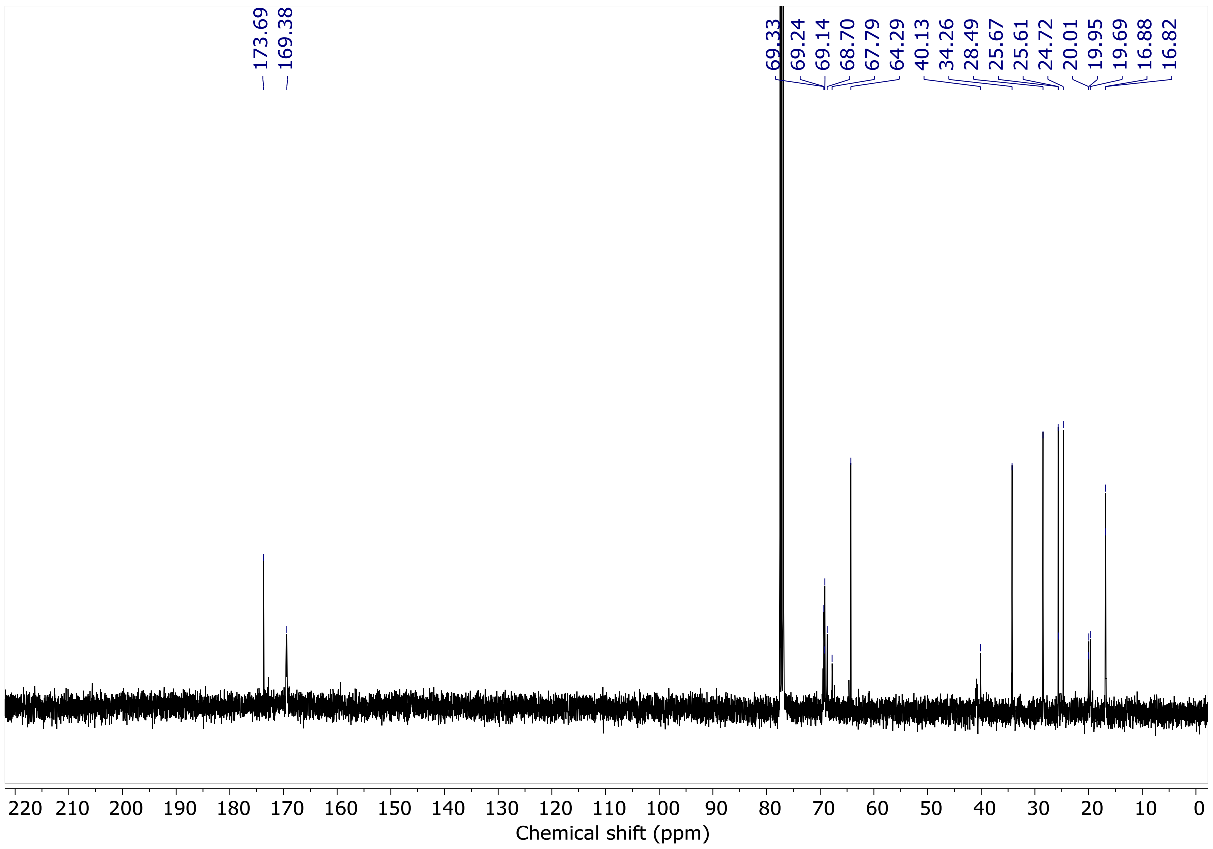

Figure S23: ^{1}H NMR Spectrum of Polymer in Table 1, Entry 7

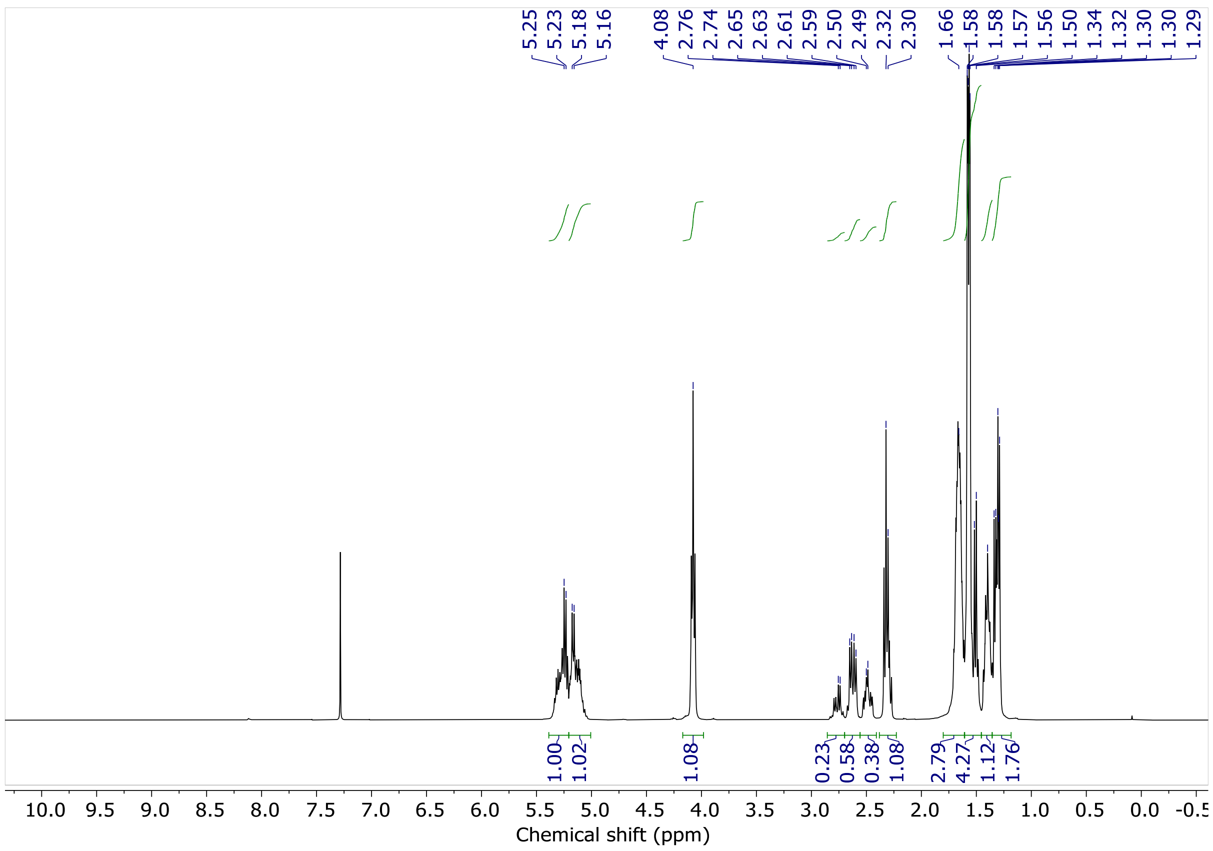

Figure S24: ^{13}C NMR Spectrum of Polymer in Table 1, Entry 7

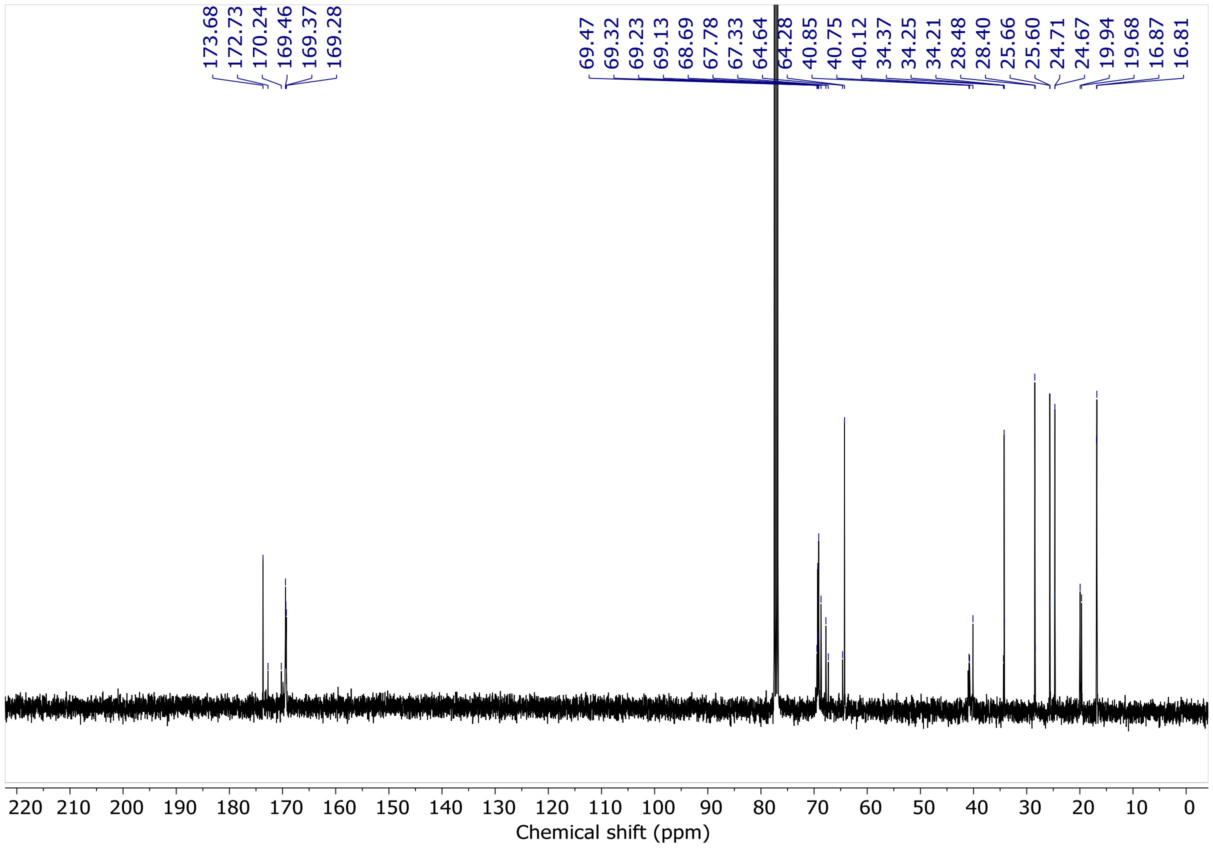

Figure S25: ^{1}H NMR Spectrum of Polymer in Table 1, Entry 8

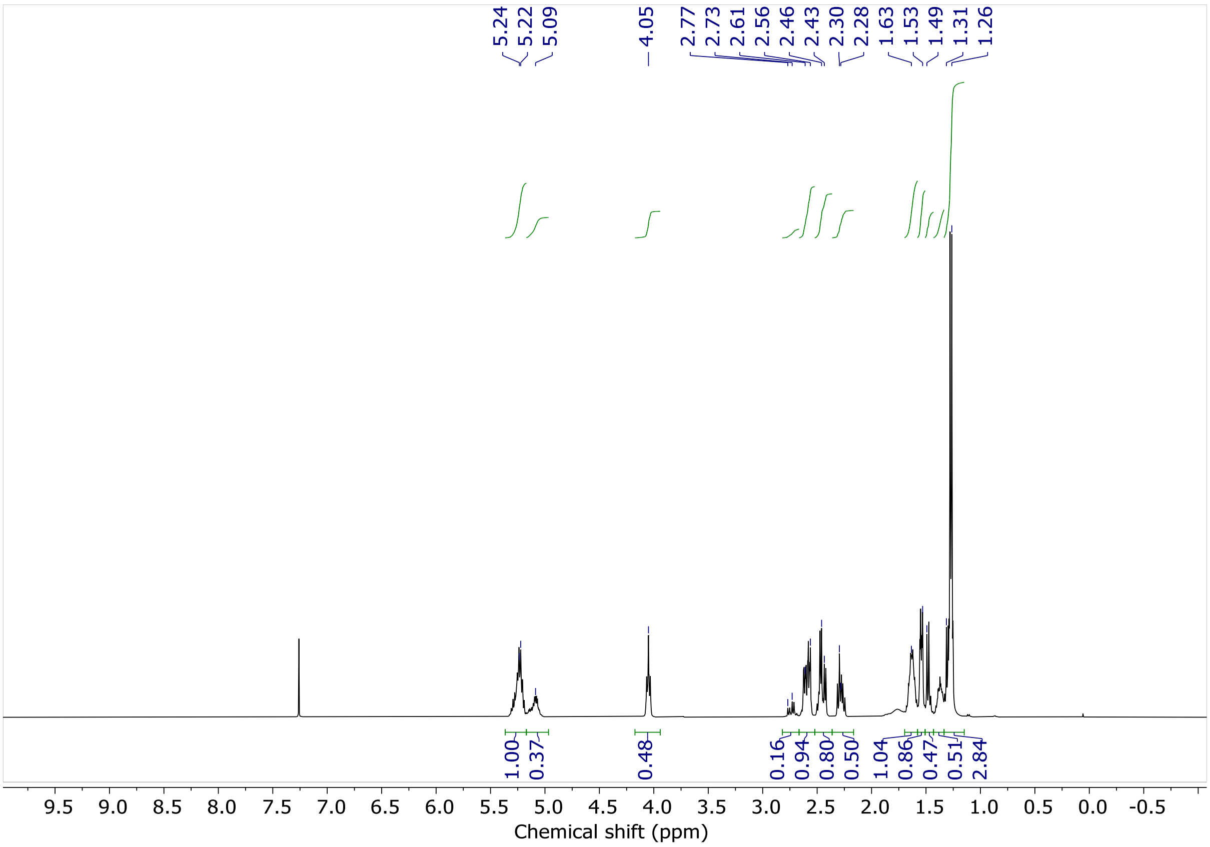

Figure S26: ^{13}C NMR Spectrum of Polymer in Table 1, Entry 8

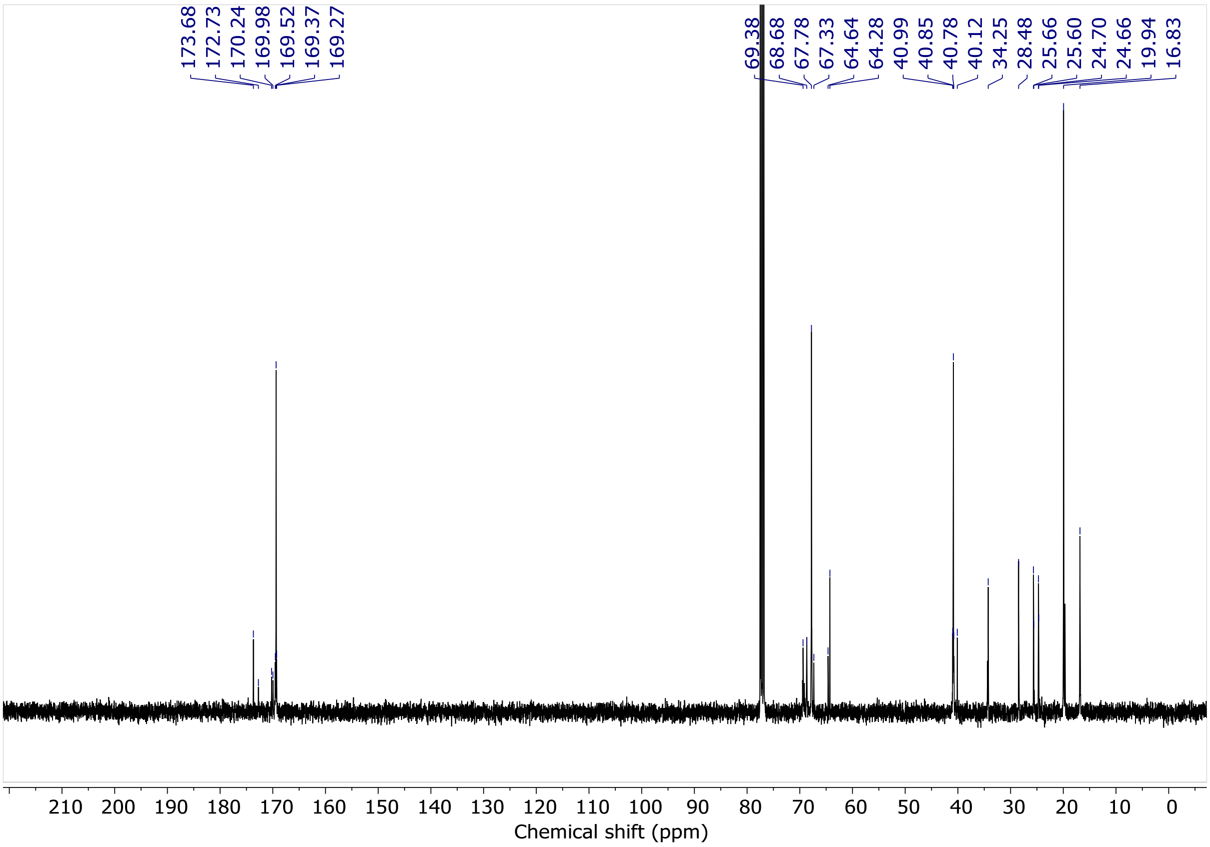

Figure S27: ^{1}H NMR Spectrum of Polymer in Table 1, Entry 9

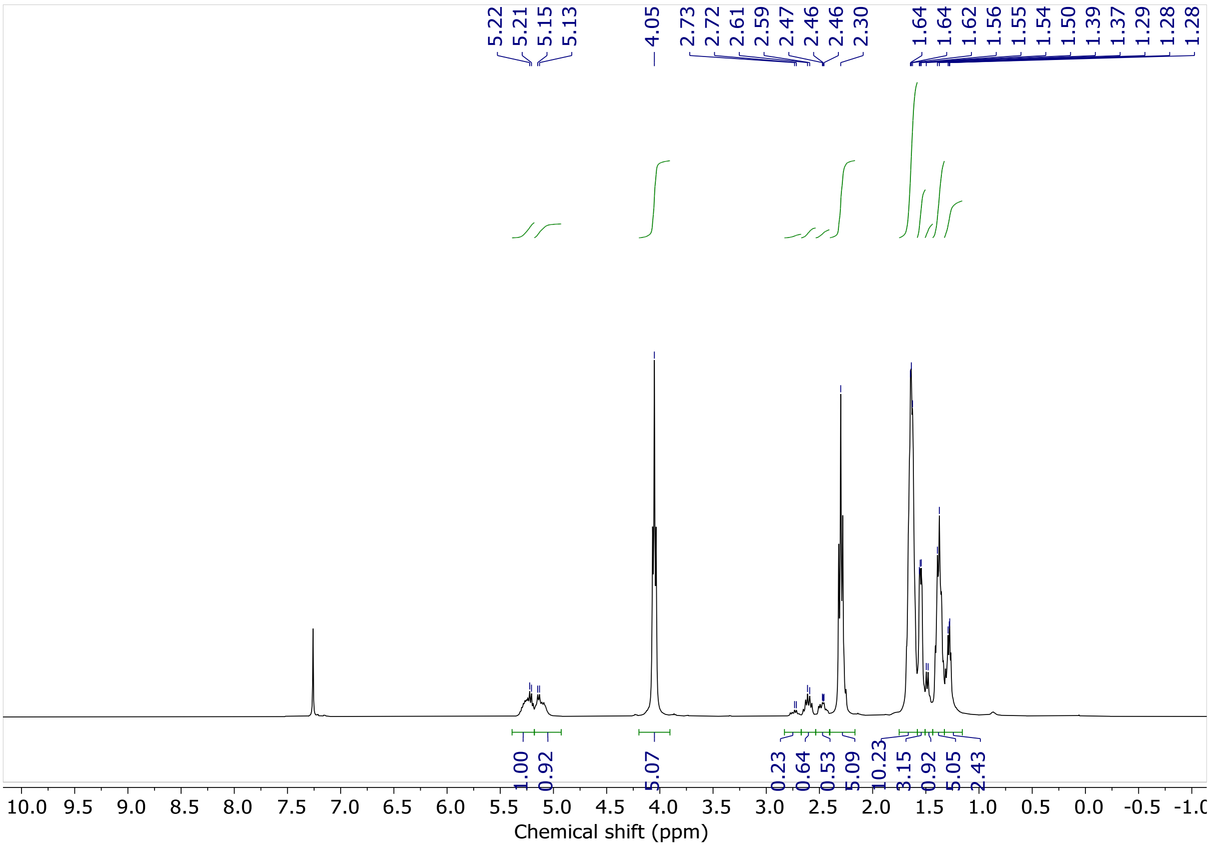

Figure S28: ^{13}C NMR Spectrum of Polymer in Table 1, Entry 9

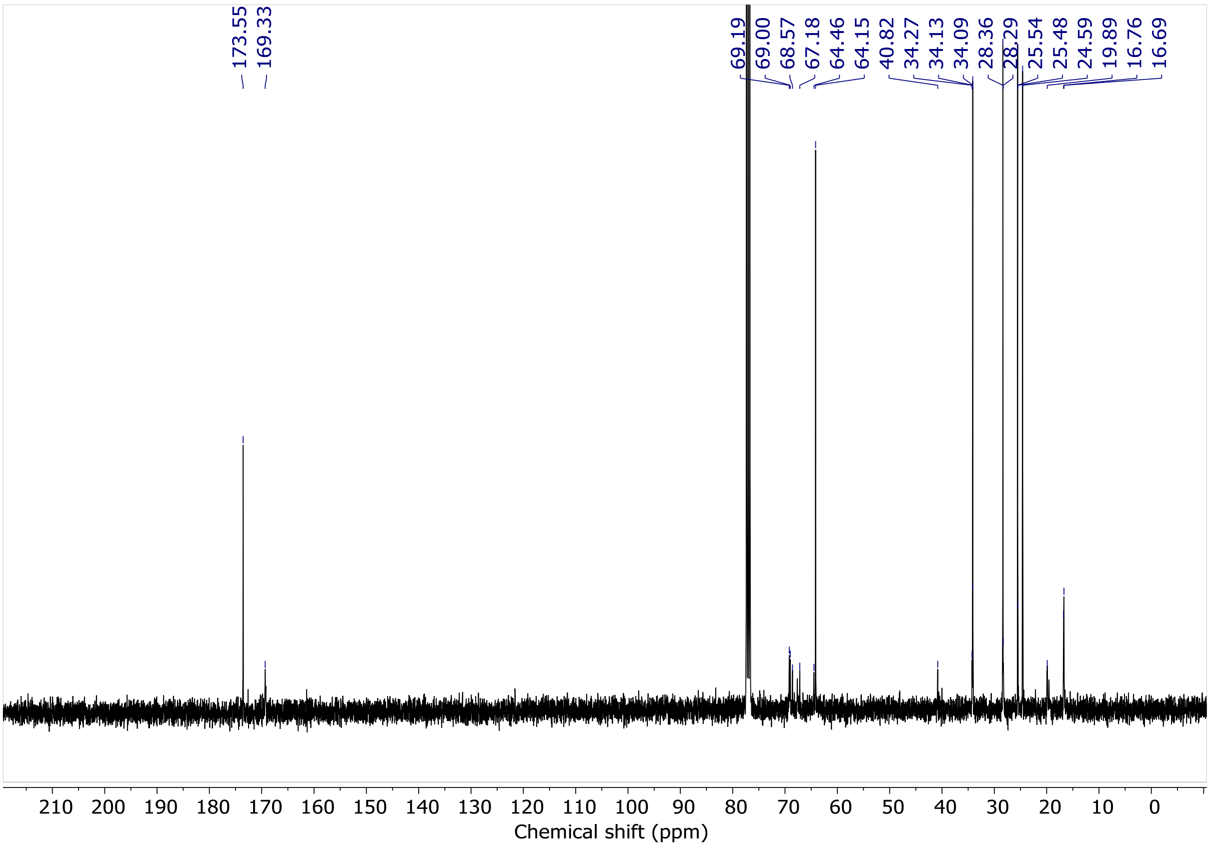

Figure S29: ^{1}H NMR Spectrum of Polymer in Table 1, Entry 10

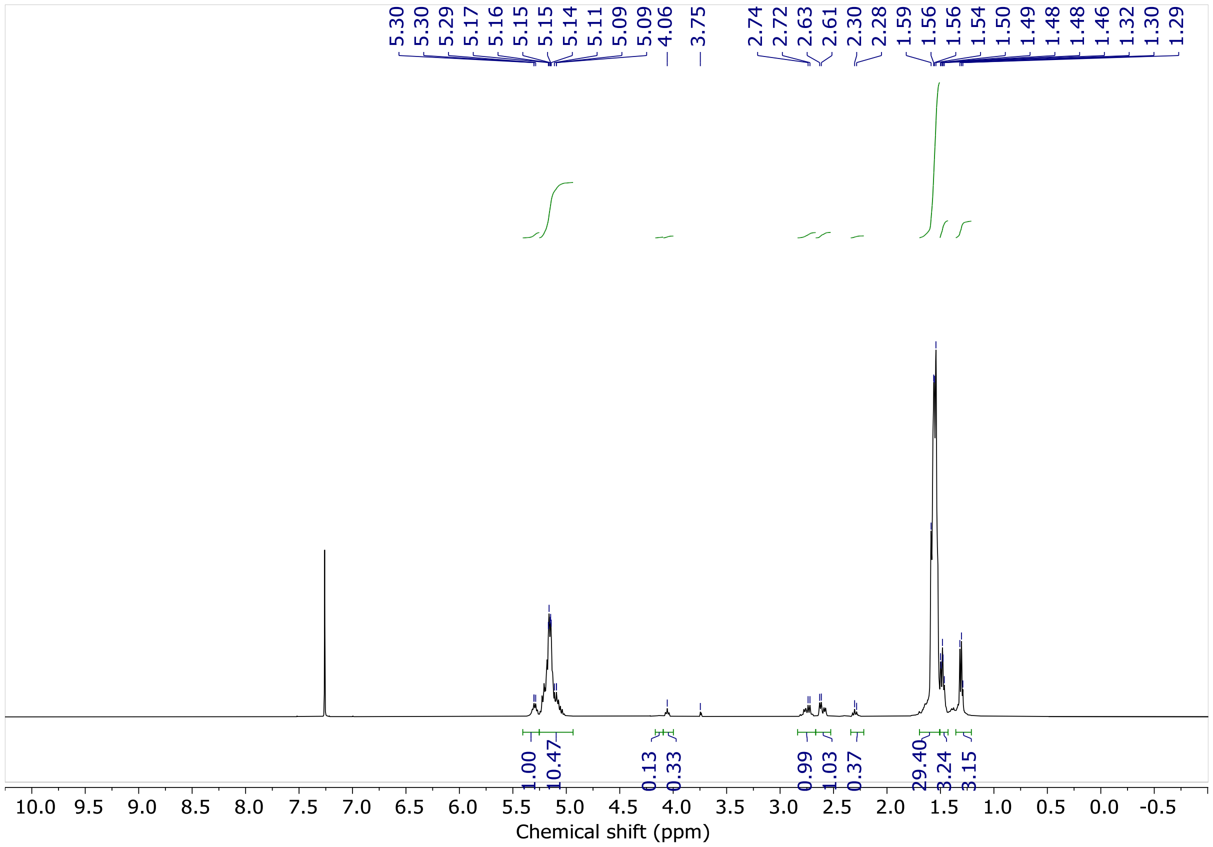

Figure S30: ^{13}C NMR Spectrum of Polymer in Table 1, Entry 10

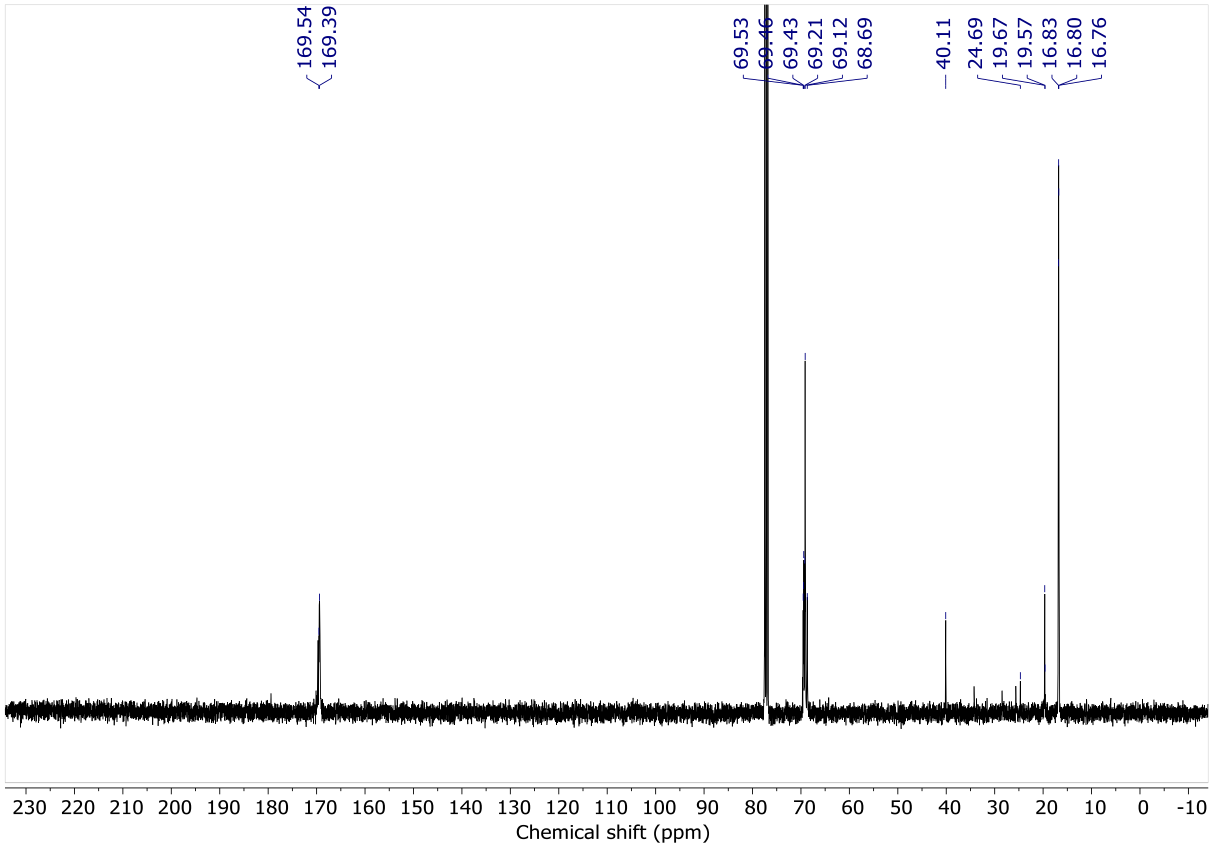

4. Representative GPC traces of copolymers

Figure S31 GPC trace from polymer in Table 1, Entry 1

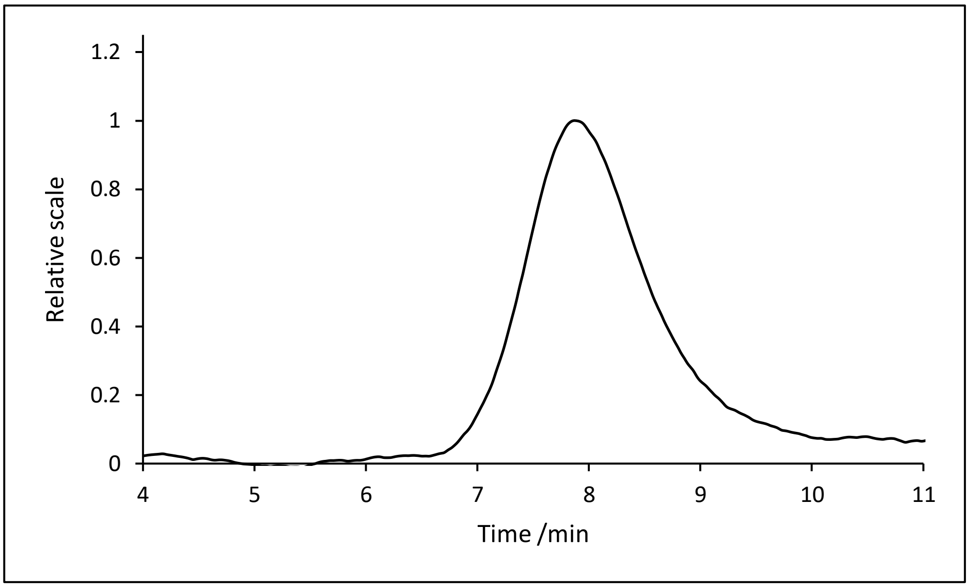

Figure S32: GPC trace from polymer in Table 1, Entry 2

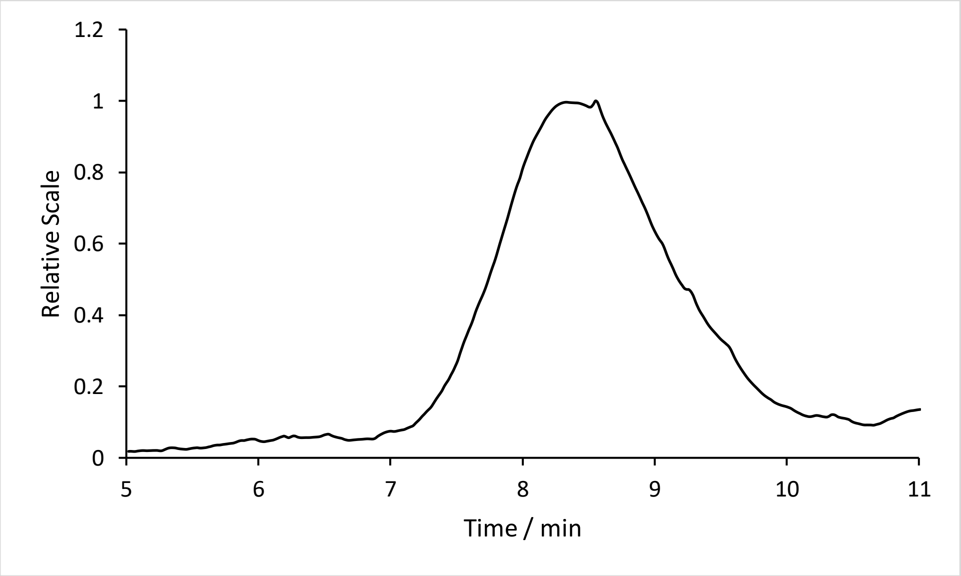

Figure S33: GPC trace from polymer in Table 1, Entry 3

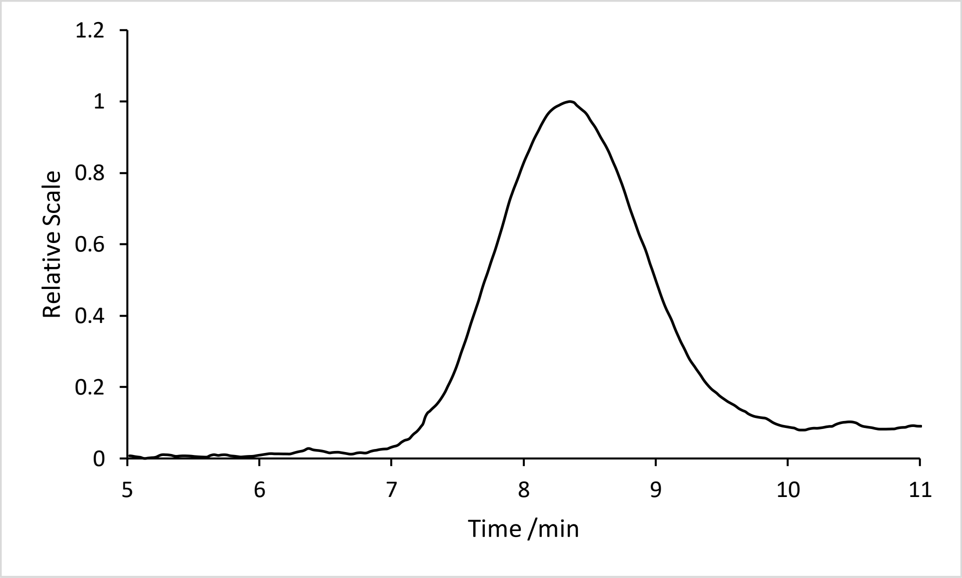

Figure S34: GPC trace from polymer in Table 1, Entry 4

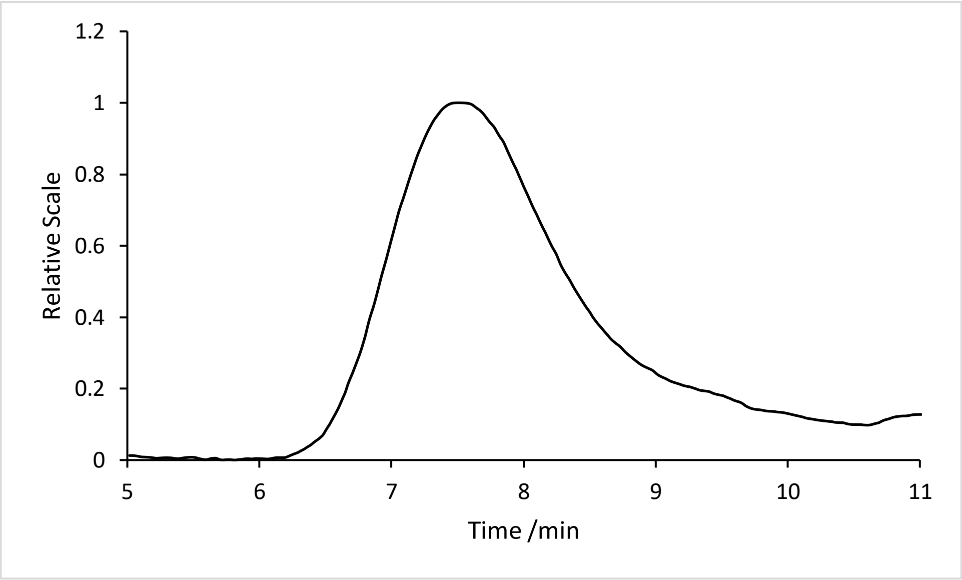

Figure S35: GPC trace from polymer in Table 1, Entry 5

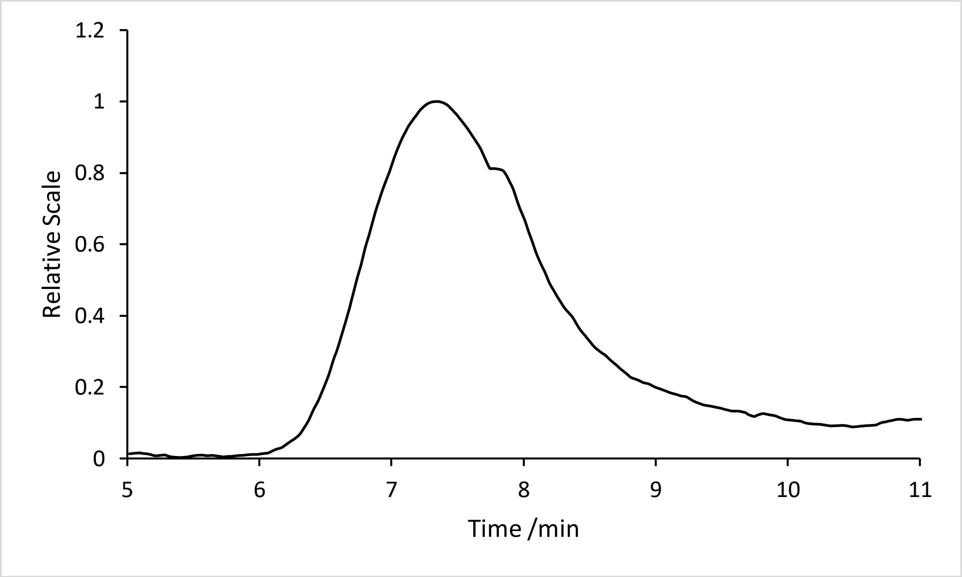

Figure S36: GPC trace from polymer in Table 1, Entry 6

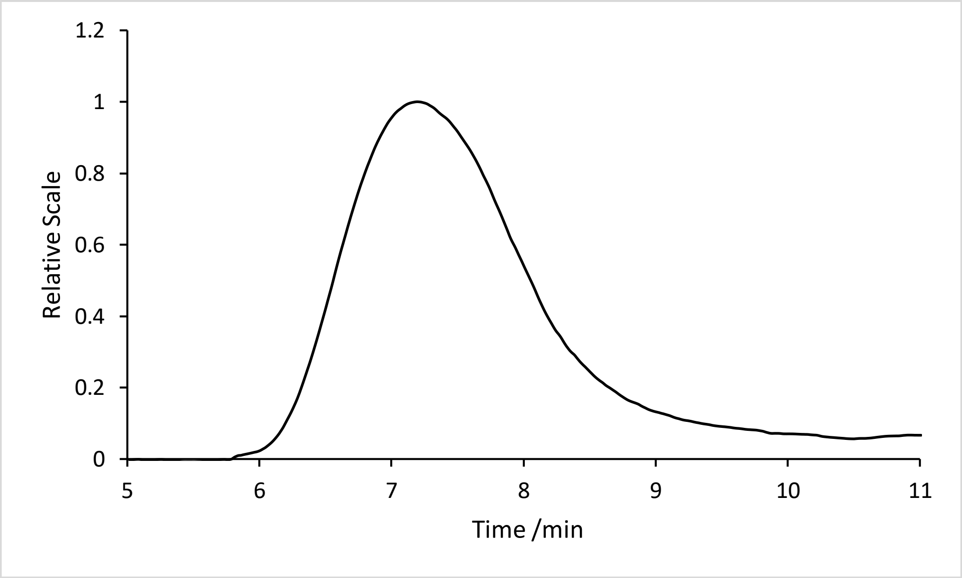

Figure S37: GPC trace from polymer in Table 1, Entry 7

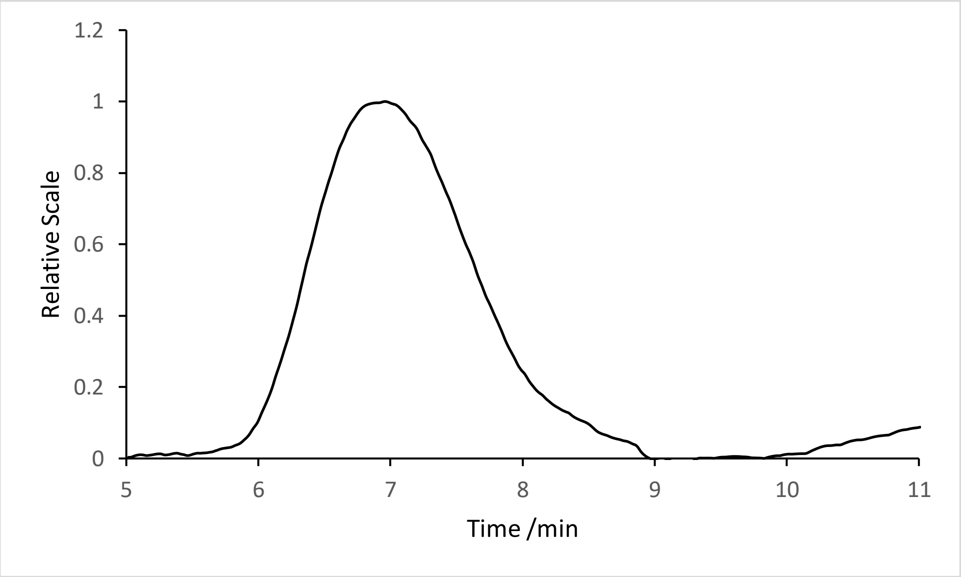

Figure S38: GPC trace from polymer in Table 1, Entry 8

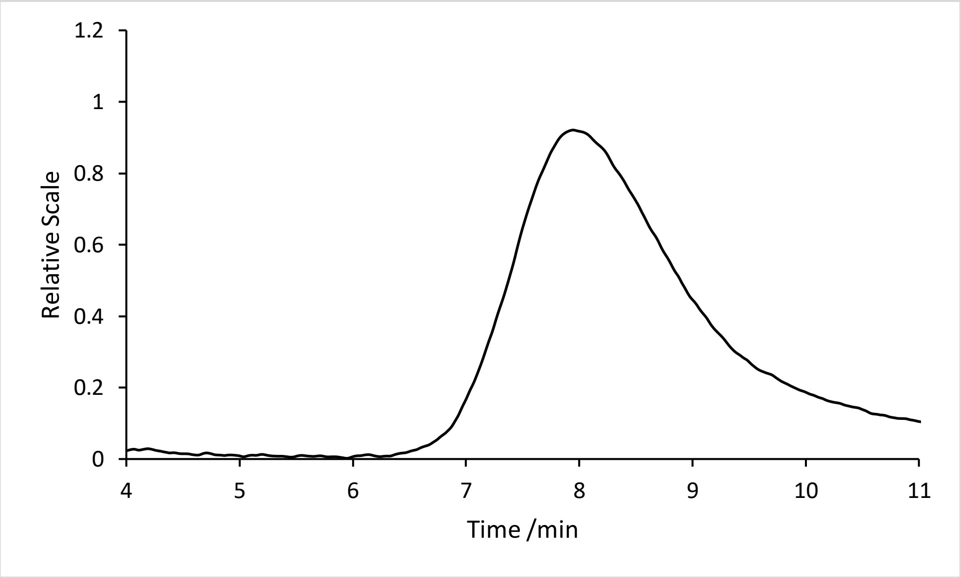

Figure S39: GPC trace from polymer in Table 1, Entry 9

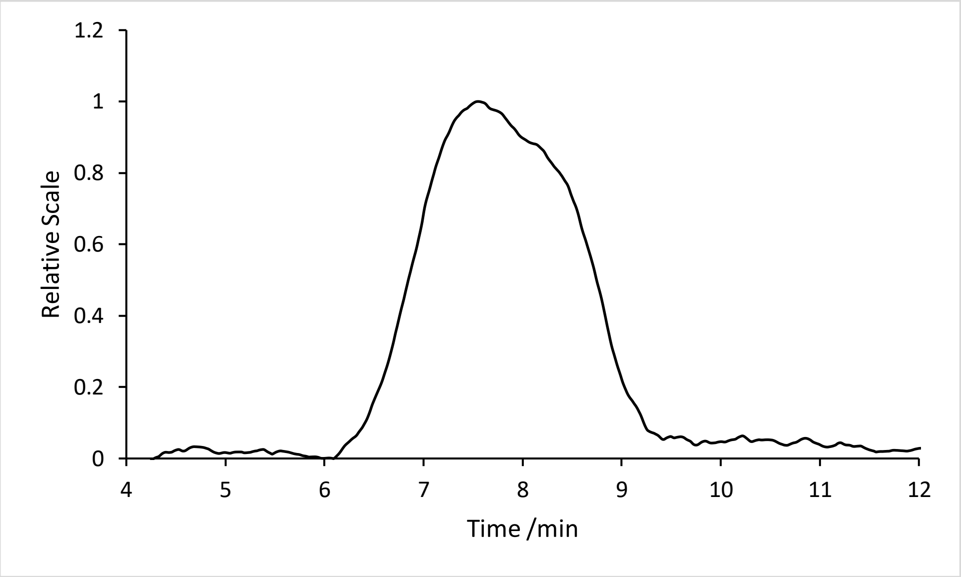

Figure S40: GPC trace from polymer in Table 1, Entry 10

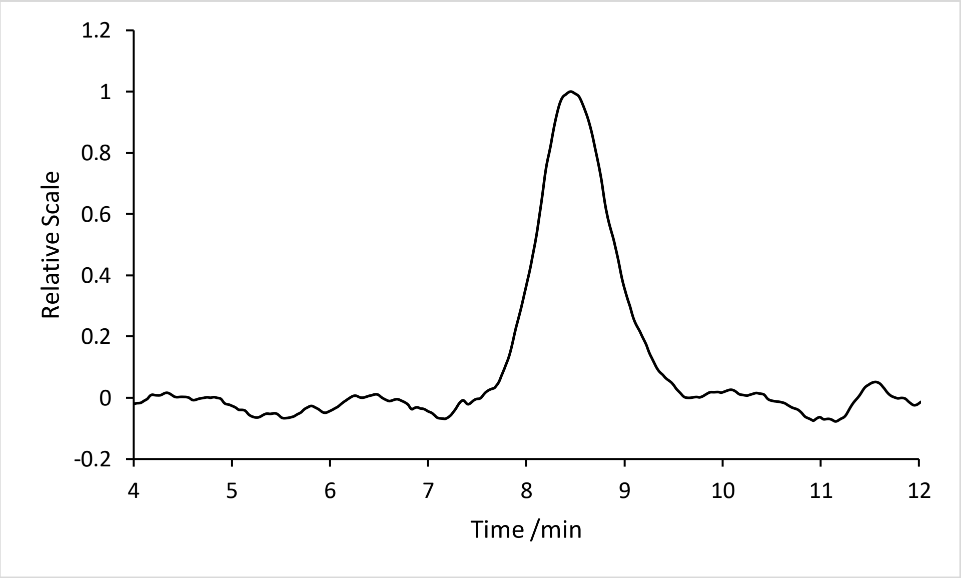

5. Polymerization Reaction and Copolymer Composition Profiles with 2 – 4

Figure S41: Plot of Monomer Conversion using $Y(N(SiHMe_2)_2)_3(THF)_2$

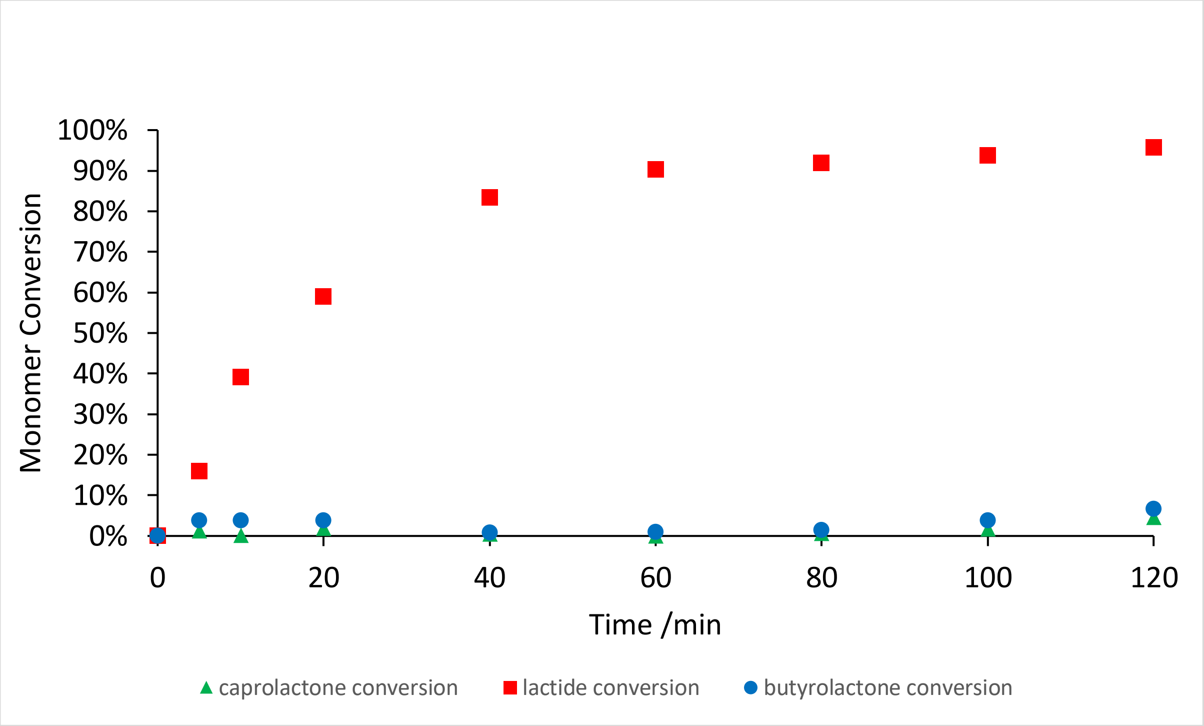

Figure S42: Plot of Monomer Conversion for Table 2, Entry 1

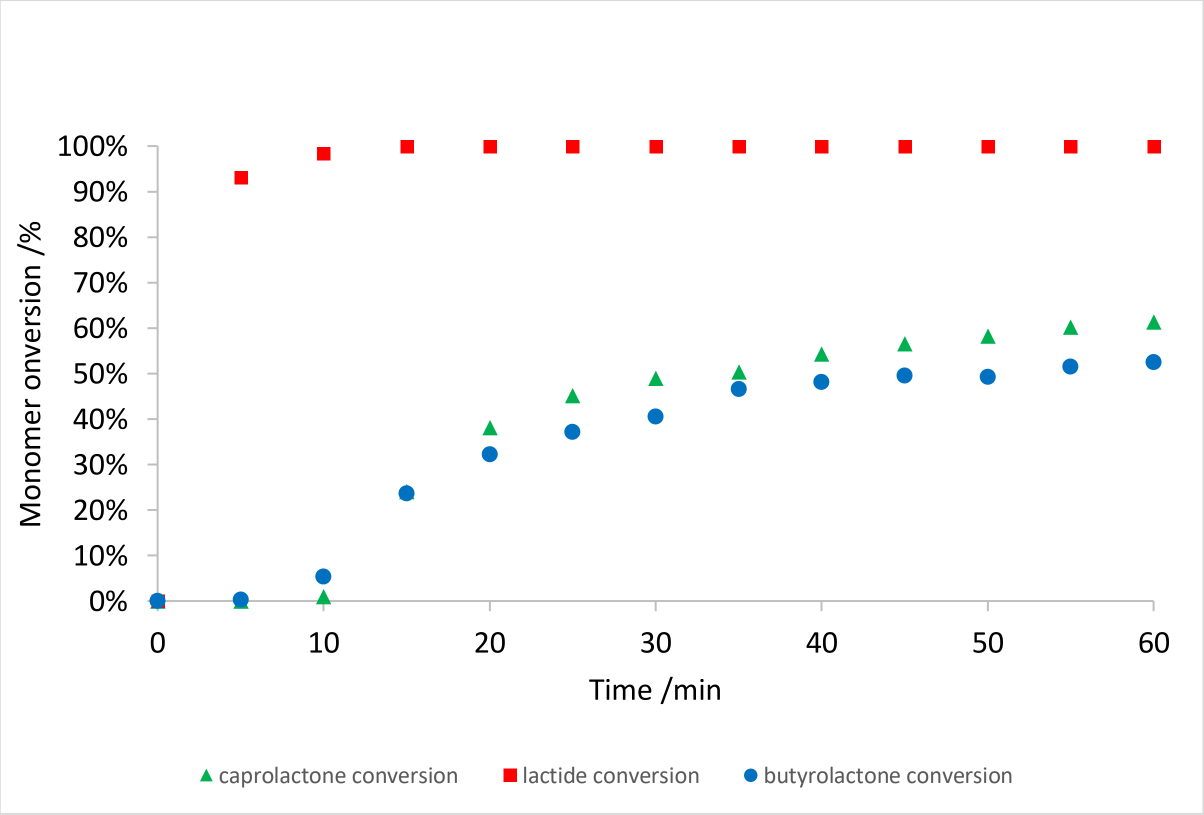

Figure S43: Plot of overall polymer composition with monomer conversion, Table 2, Entry 1

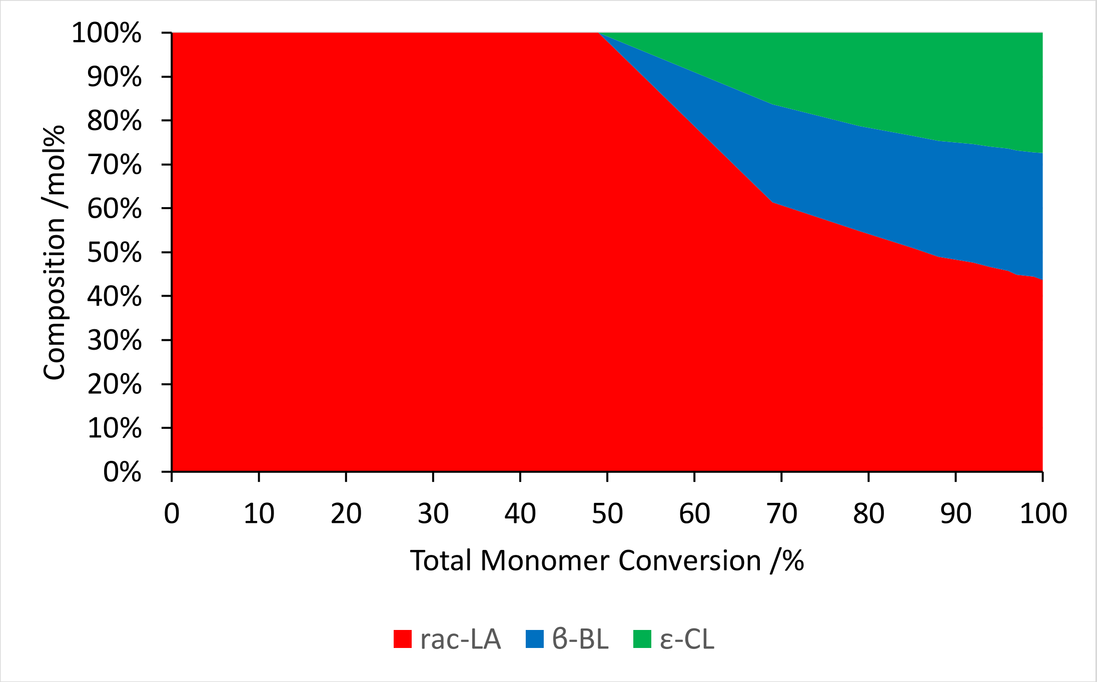

Figure S44: Plot of Monomer Conversion for Table 2, Entry 2

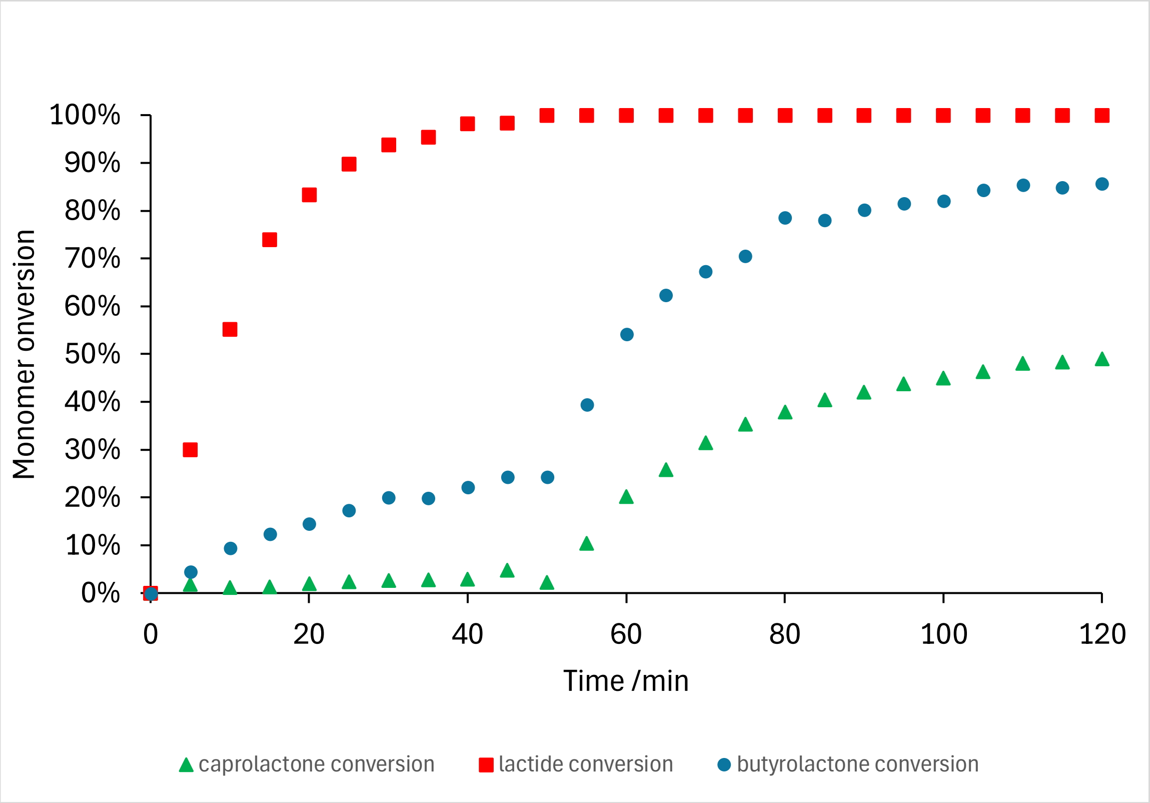

Figure S45: Plot of overall polymer composition with monomer conversion, Table 2, Entry 2

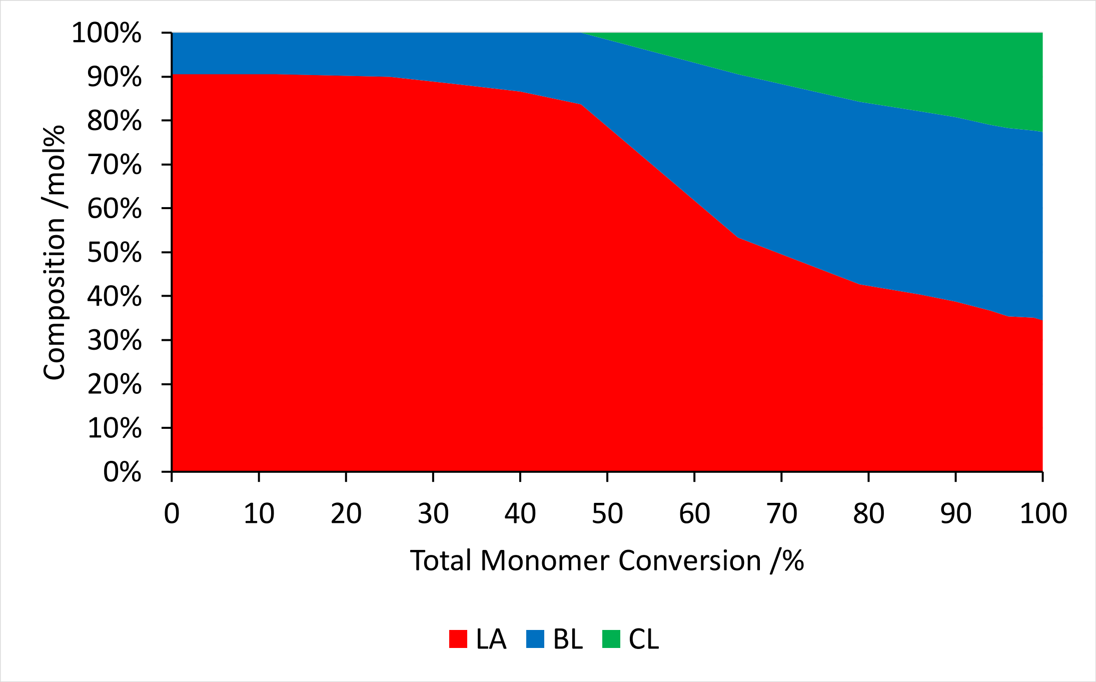

Figure S46: Plot of Monomer Conversion for Table 2, Entry 3

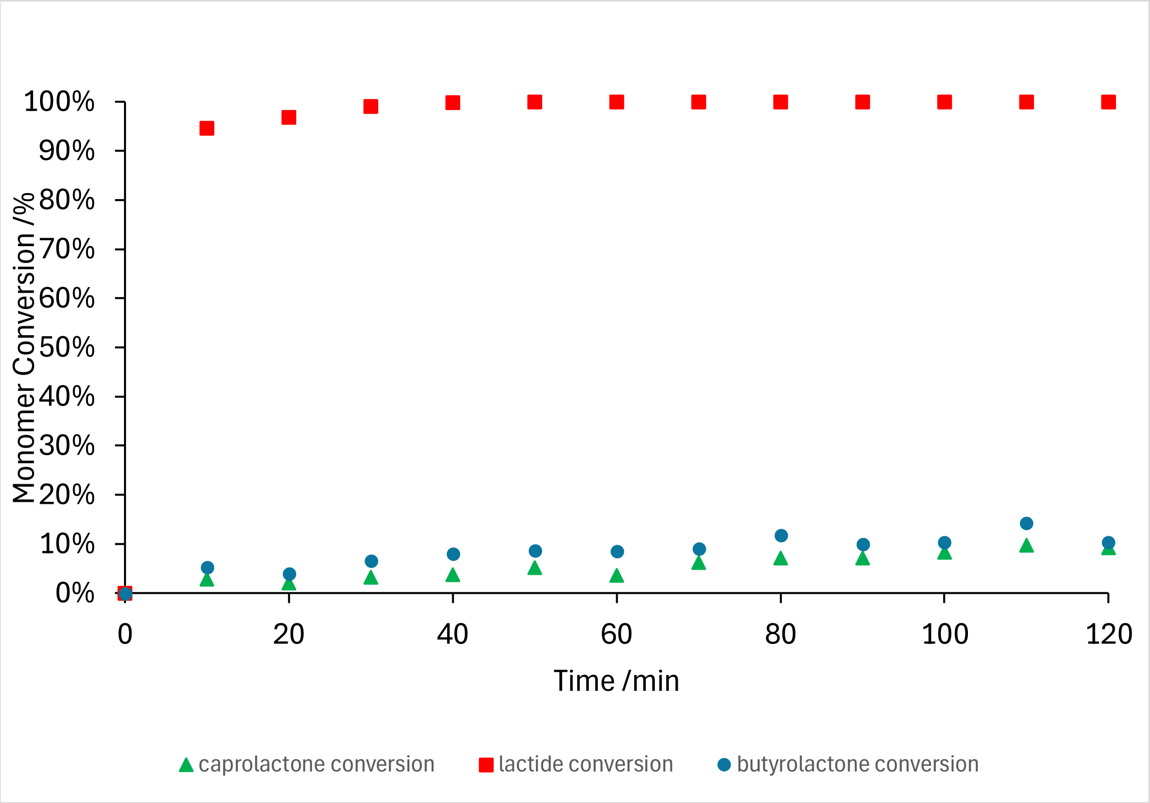

6. DSC Data for Polymers

Table S1: Thermal properties of $(AB)_x(BC)_y$ block copolymers[a]

Entry[b]	Polymer composition[c]	T_g1 /°C[d]	T_g2 /°C[d]	T_m /°C[d]	ΔH_m /J g^{-1}	T_c /°C[e]	ΔH_m /J g^{-1}
1	33:34:33	-36.6	17.9	-	-	-	-
8	17:66:17	-15.1	-	122.4	17.1	69.4	-16.4
9	17:17: 66	-34.8	-	44.4	34.3	9.4	-33.6
10	83:15:2	43.2	-	-	-	-	-

[a] Determined by DSC. Heating and cooling rates were 10 °C min^{-1}. Blank entries correspond to an absence of the transition. [b]Number corresponds to entry in table 1 (main manuscript). [c]Determined by ^{1}H NMR spectrum. [d]Taken from the second heating cycle. [e]Taken from the first cooling cycle.

7. Additional Experiments

Figure S47: Plot of Monomer Conversion for Table 1, Entry 8

Monomer conversion has been normalised so that the maximum percentage conversion reflects the monomer feed ratio (17:66:17 *rac*-LA:*rac*-β-BL:ε-CL)

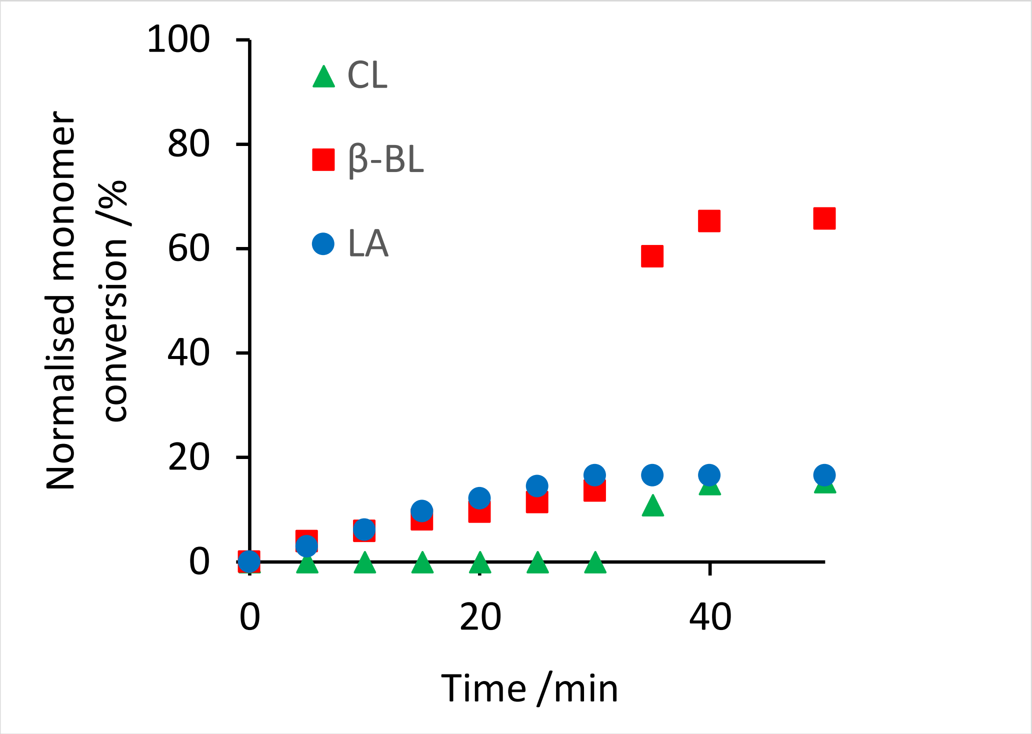

Figure S48: Plot of Monomer Conversion for Table 1, Entry 9

Monomer conversion has been normalised so that the maximum percentage conversion reflects the monomer feed ratio (17:17:66 *rac*-LA:*rac*-β-BL:ε-CL)

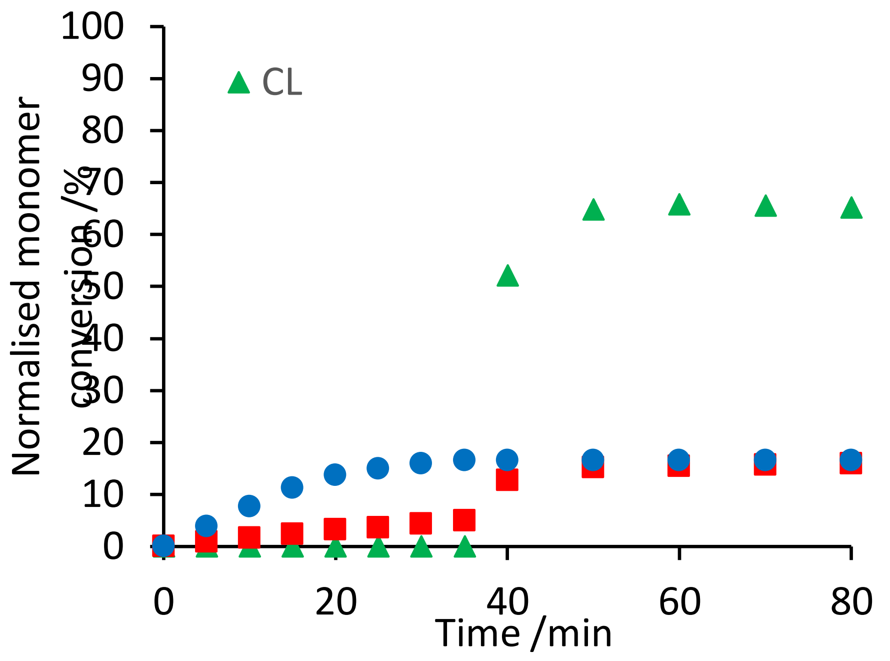

Figure S49: Plot of Monomer Conversion for Table 1, Entry 9

Monomer conversion has been normalised so that the maximum percentage conversion reflects the monomer feed ratio (17:17:66 *rac*-LA:*rac*-β-BL:ε-CL)

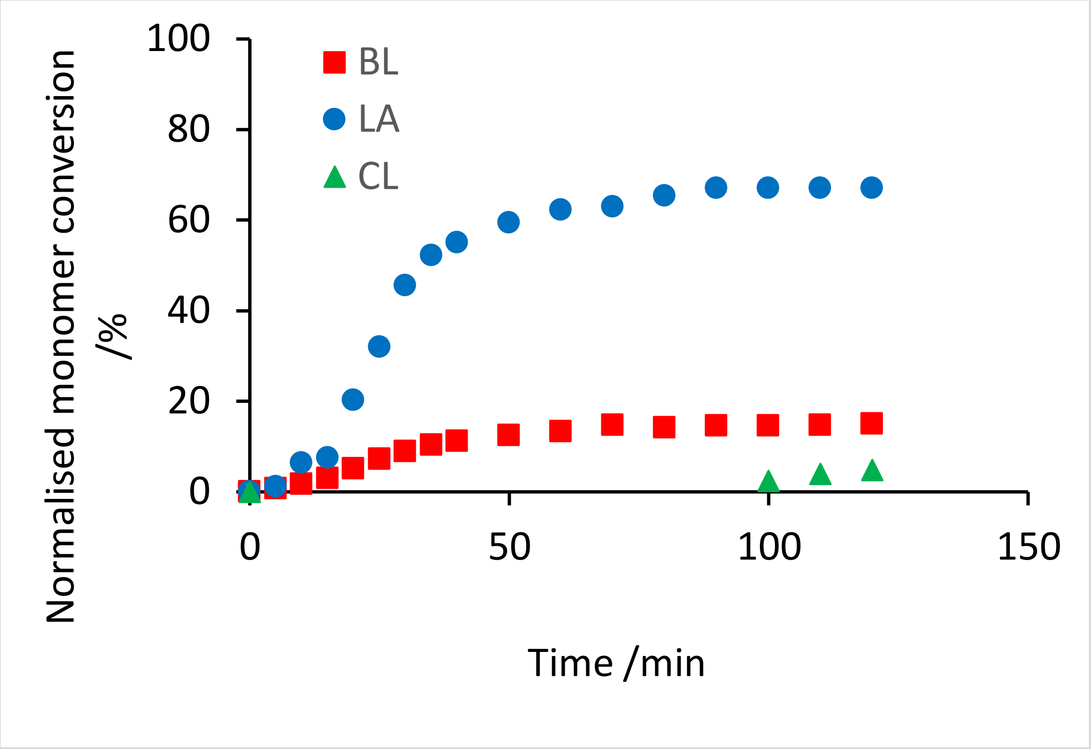

Figure S50: BASHD (band selective homonuclear decoupled) ^{1}H NMR spectrum of PLA

Polymerized using **1** in the presence of 200 eqiv. ε-CL in toluene at ambient temperature ($[LA]_0$ = $[\varepsilon\text{-CL}]_0$ = 0.25, $[monomer]_0/[\mathbf{1}]$ = 200) and showing the region 5.28-5.14 ppm.

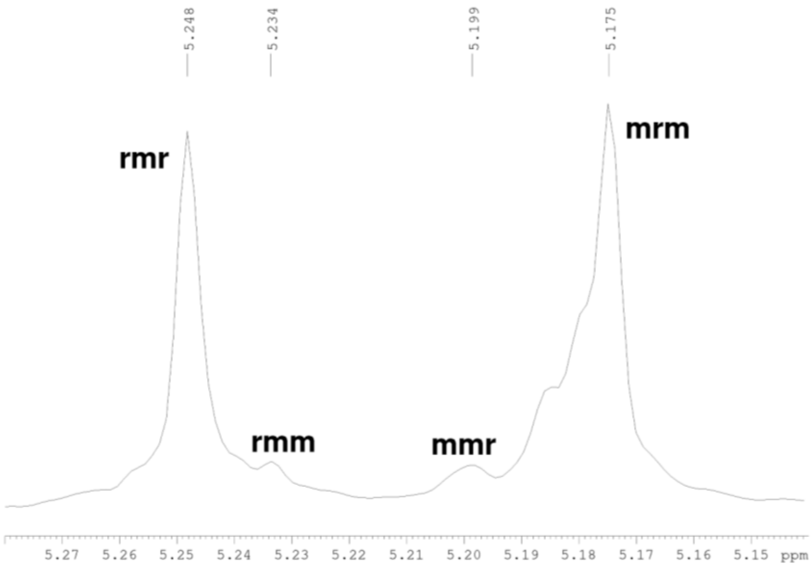

Figure S51: Semi-logarithmic plot of the polymerization of *rac*-LA in the presence of ε-CL and *rac*-β-BL

Reaction conditions: $[LA]_0$ = $[\varepsilon\text{-CL}]_0$ =[*rac*-β-BL] = 0.25 in 8 mL toluene at ambient temperature, $[\text{monomer}]_0/[\mathbf{1}]$ = 300.

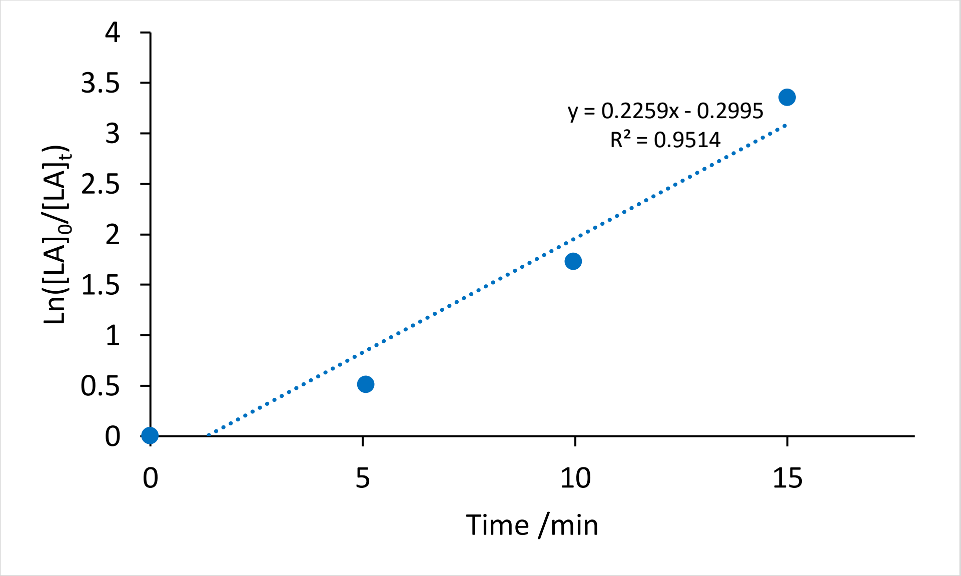

Figure S52: Semi-logarithmic plot of the polymerization of *rac*-LA in the presence of ε-CL

Reaction conditions: $[LA]_0$ = $[\varepsilon\text{-CL}]_0$ = 0.25 in 8 mL toluene at ambient temperature, $[\text{monomer}]_0/[\mathbf{1}]$ = 300.

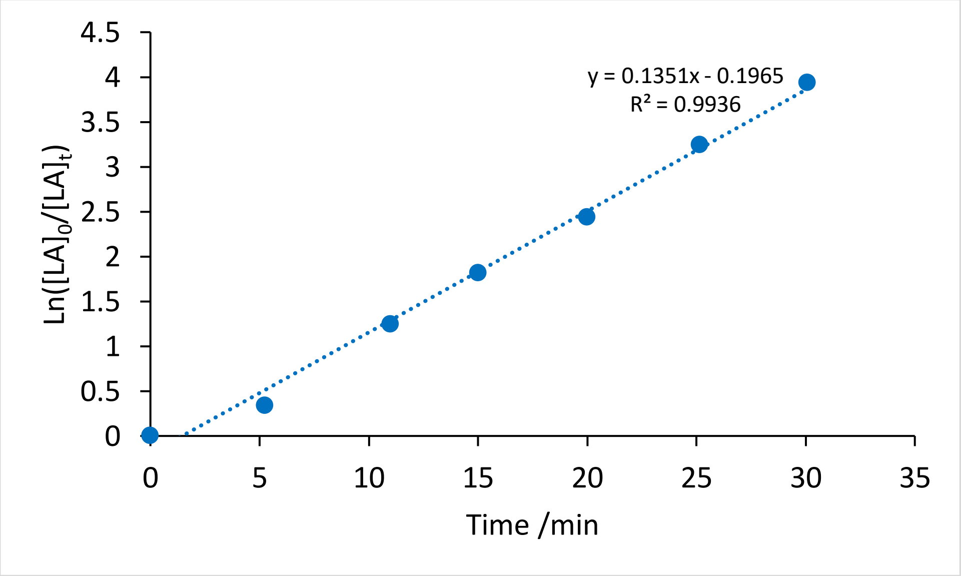

Figure S53: Plot to show conversion over time in the copolymerization of *rac*-β-BL and ε-CL by **1**.

Reaction conditions: $[LA]_0$ = $[\varepsilon\text{-CL}]_0$ = 0.25 in 8 mL toluene at ambient temperature, ($[rac\text{-}\beta\text{-BL}]_0$ + $[\varepsilon\text{-CL}]_0$)/ [**1**] = 400. *Rac*-β-BL conversion is shown by red squares, ε-CL is shown by green triangles.

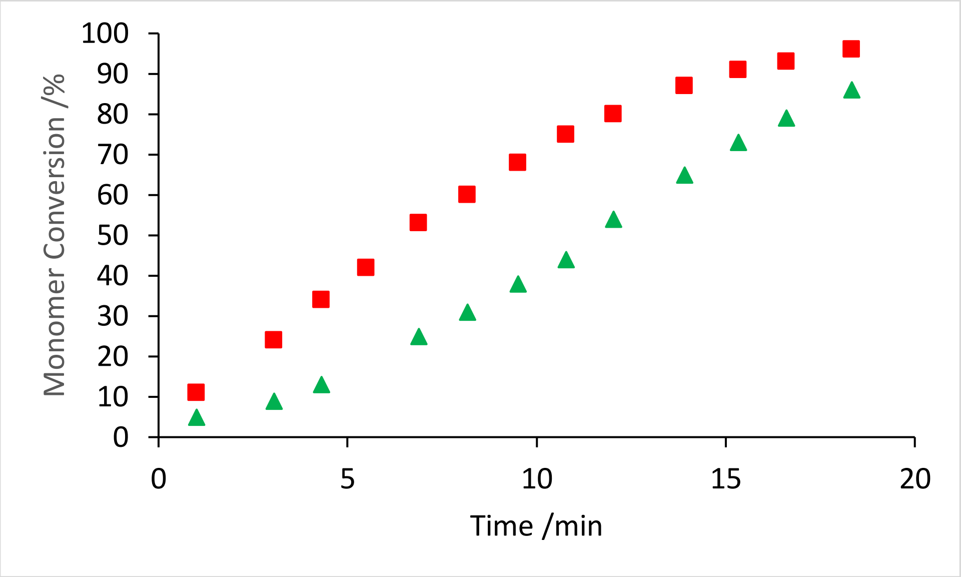

Figure S54: Semilogarithmic plot for ε-CL polymerization between 4min and 20 min in the copolymerization of *rac*-β-BL and ε-CL by **1.**

Reaction conditions: $[LA]_0 = [ε\text{-}CL]_0 = 0.25$ in 8 mL toluene at ambient temperature, ($[rac\text{-}β\text{-}BL]_0 + [ε\text{-}CL]_0$)/ [**1**] = 400.

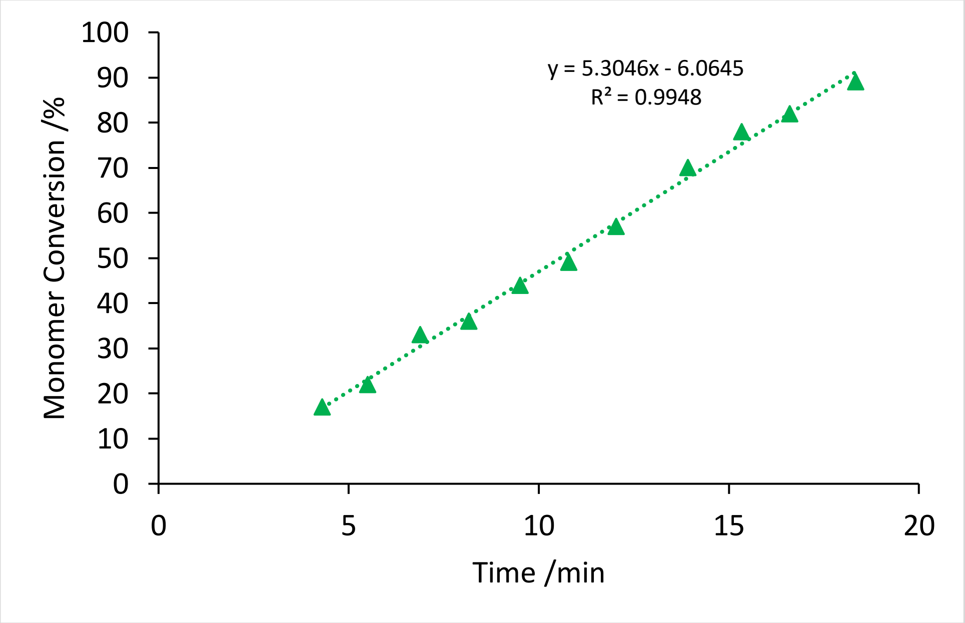

Figure S55: Semilogarithmic plot for *rac*-β-BL polymerization between 4 min and 17 min in the copolymerization of *rac*-β-BL and ε-CL by **1.**

Reaction conditions: $[LA]_0 = [ε\text{-}CL]_0 = 0.25$ in 8 mL toluene at ambient temperature, ($[rac\text{-}β\text{-}BL]_0 + [ε\text{-}CL]_0$)/ [**1**] = 400.

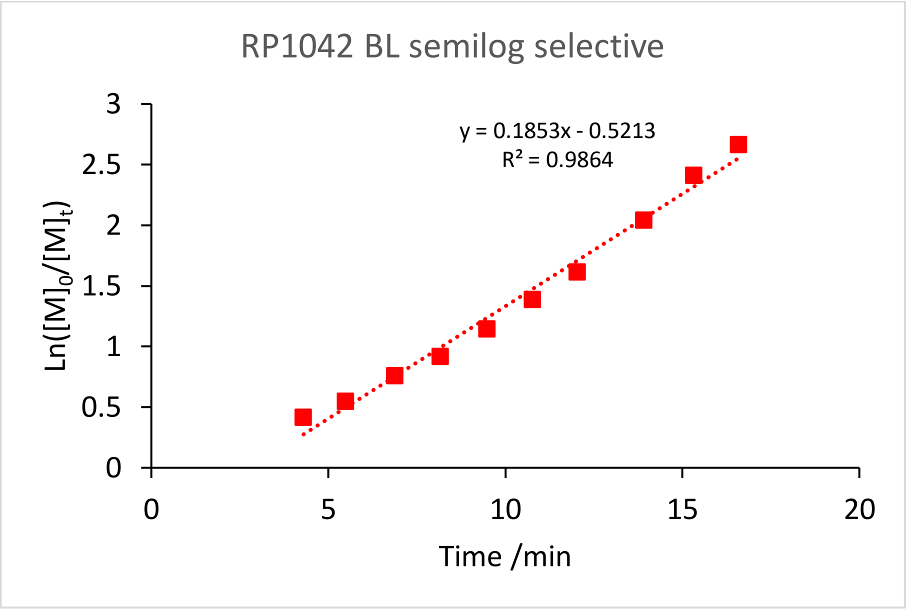

Figure S56: ^{13}C NMR spectra of aliquots removed from a copolymerization reaction after 2 h (bottom spectrum) and 24 h (top spectrum).

Reaction conditions: [*rac*-LA]$_0$=[*rac*-β-BL]$_0$=[ε-CL]$_0$) = 0.25 M in 8 mL toluene at ambient temperature, [Monomer]$_0$/[**Y**]= 200,

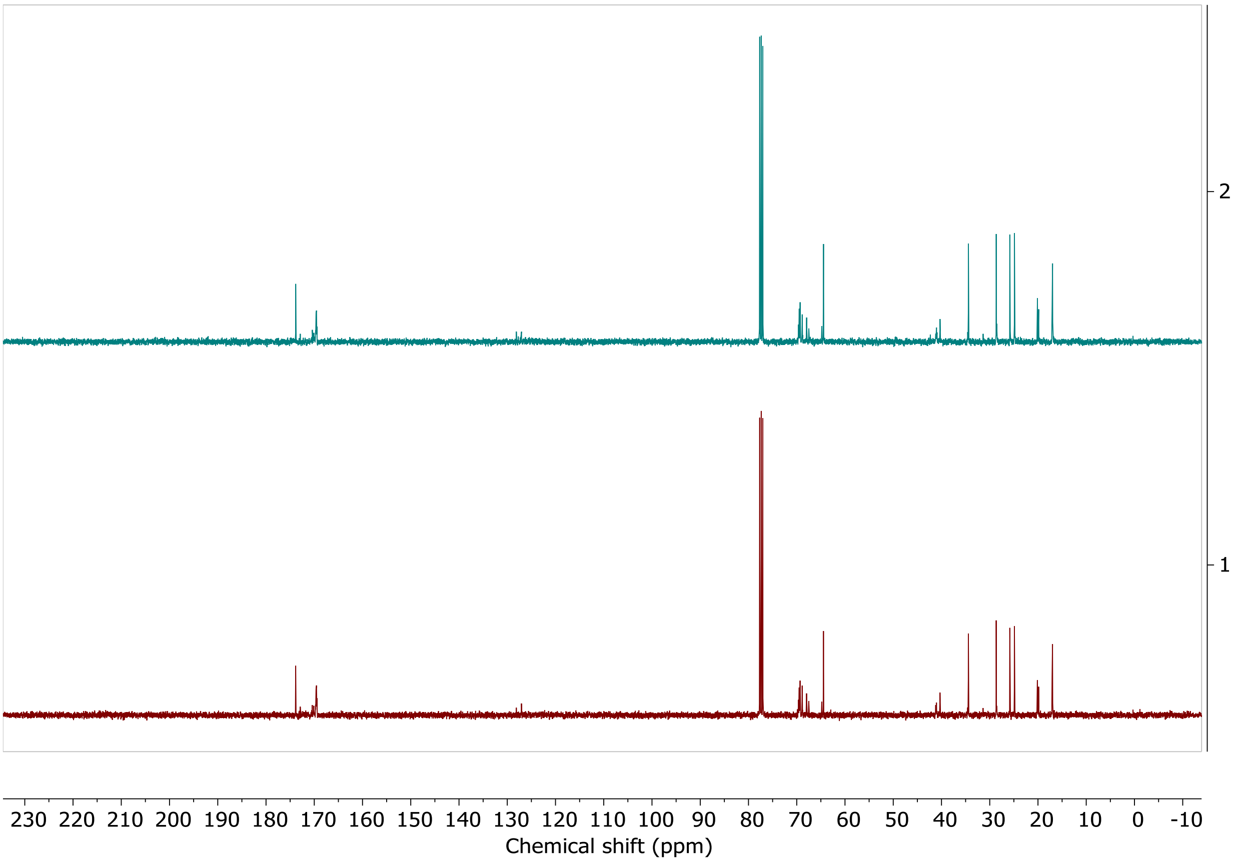

Figure S57: ^{13}C NMR spectra of aliquots removed from a copolymerization reaction after 30 min (red trace, bottom), and 2 h (green trace, top).

The bottom spectrum (LA = 97% conversion, rac-β-BL = 37% conversion, ε-CL = 0% conversion) shows the absence of BB sequences in the copolymer (no signal at 67.6 ppm) and the presence of BL/LB/LL sequences.

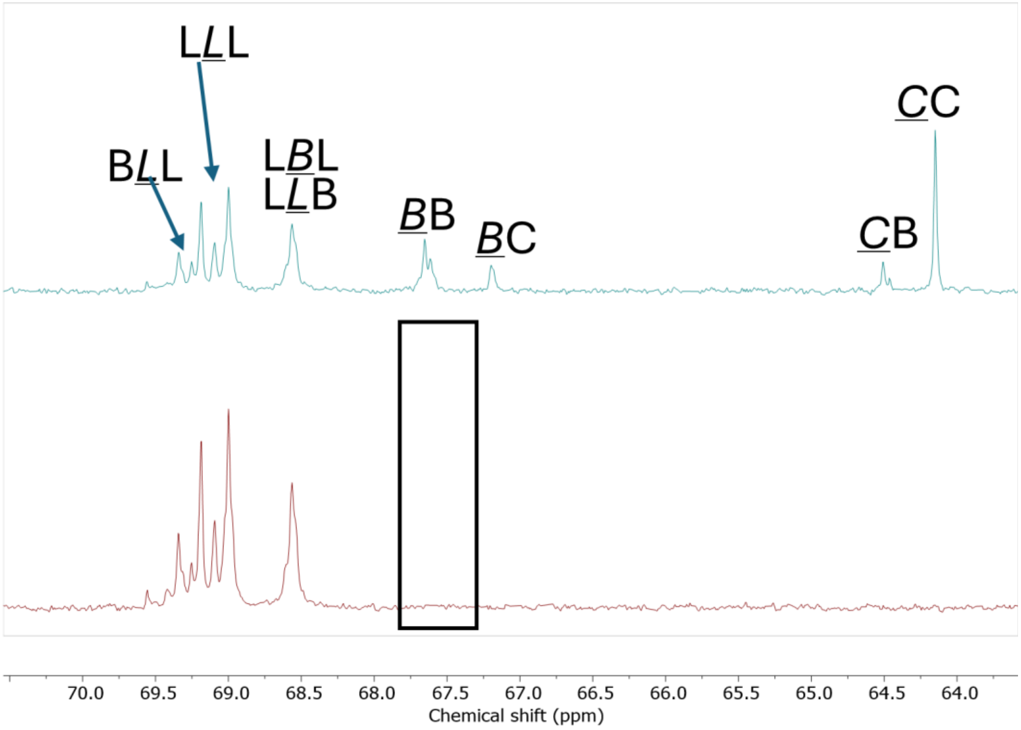

Figure S58: ^{1}H NMR Spectrum obtained after mixing **1** with 1 eq. (*S*)-Ethyl lactate in C_6D_6

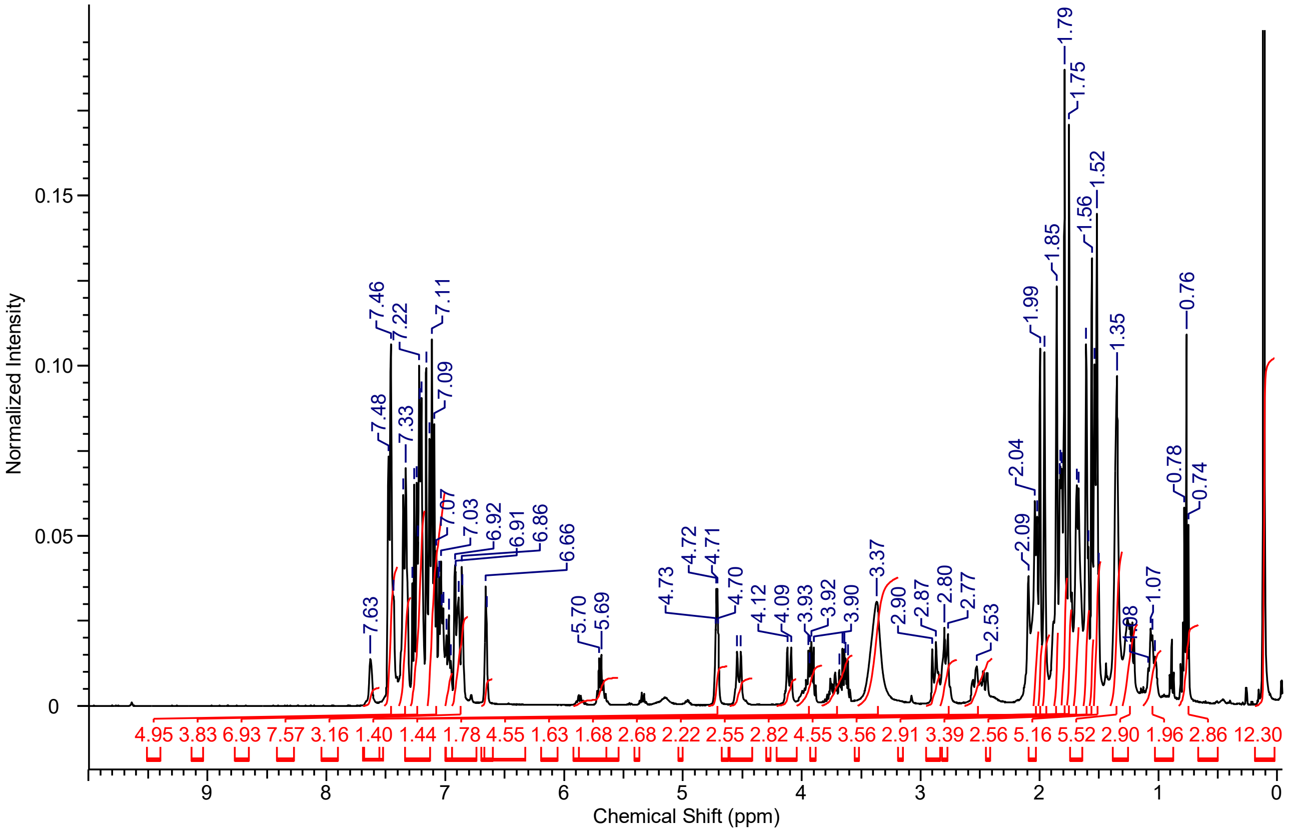

Figure S59: ^{13}C NMR Spectrum obtained after mixing **1** with 1 eq. (*S*)-Ethyl lactate in C_6D_6

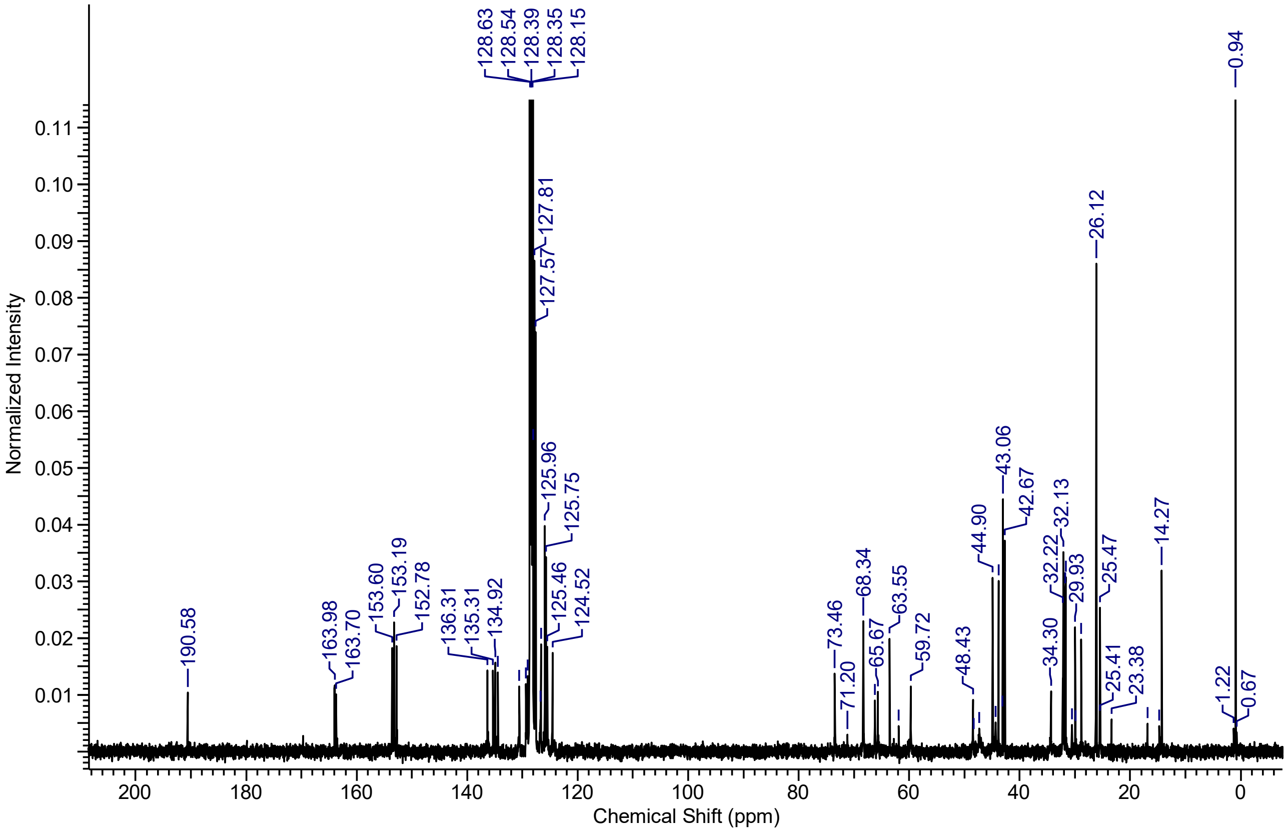

Figure S60: ^{1}H NMR Spectrum obtained after mixing **1** with 1 eq. (*R*)-methyl-3-hydroxybutyrate in C_6D_6

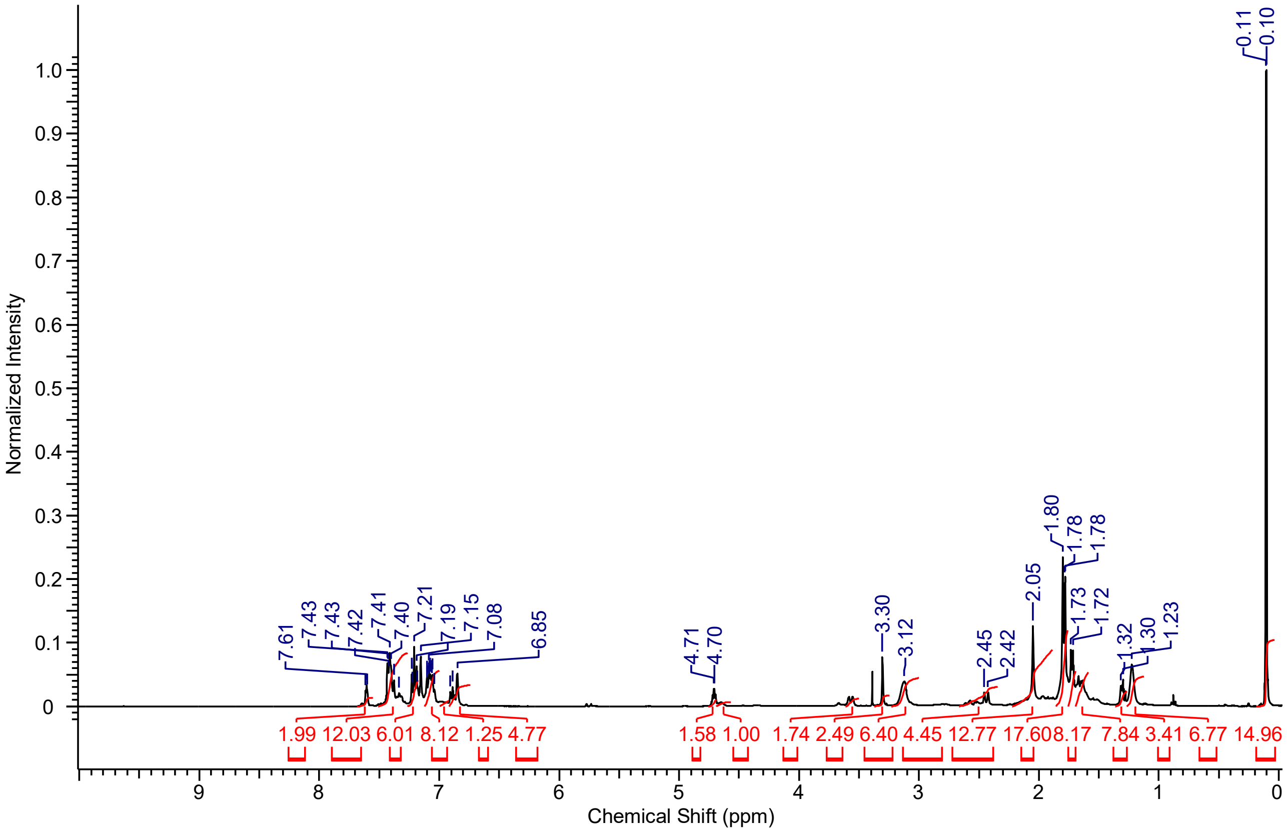

Figure S61: ^{13}C NMR Spectrum obtained after mixing **1** with 1 eq. (*R*)-methyl-3-hydroxybutyrate in C_6D_6

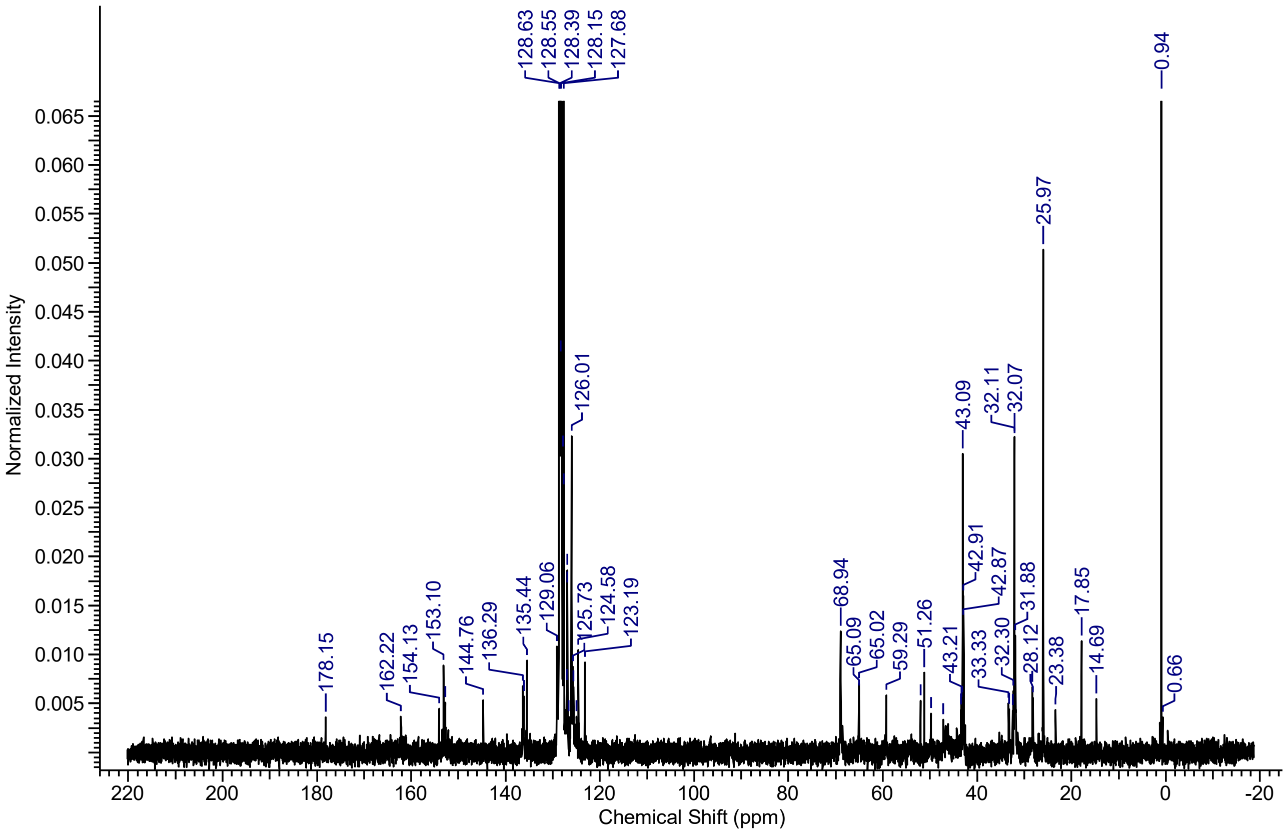

Experiment to compare copolymer composition in aliquots removed from a reaction mixture before and after purification.

Table S2: Comparison of copolymer composition before and after precipitation.

	Time/min	Yield /%	Copolymer composition /mol%		
			LL	B	C
**crude**	20		77	23	0
	30		36	37	27
	35		34	37	29
	60		33	36	31
**purified**	20	64	78	22	0
	30	78	36	36	28
	35	76	35	35	30
	60	81	33	35	31

Copolymer composition was determined from ^{1}H NMR spectra of crude samples after removal from the reaction mixture and copolymer purified by precipitation.

The overall reaction profile is shown below:

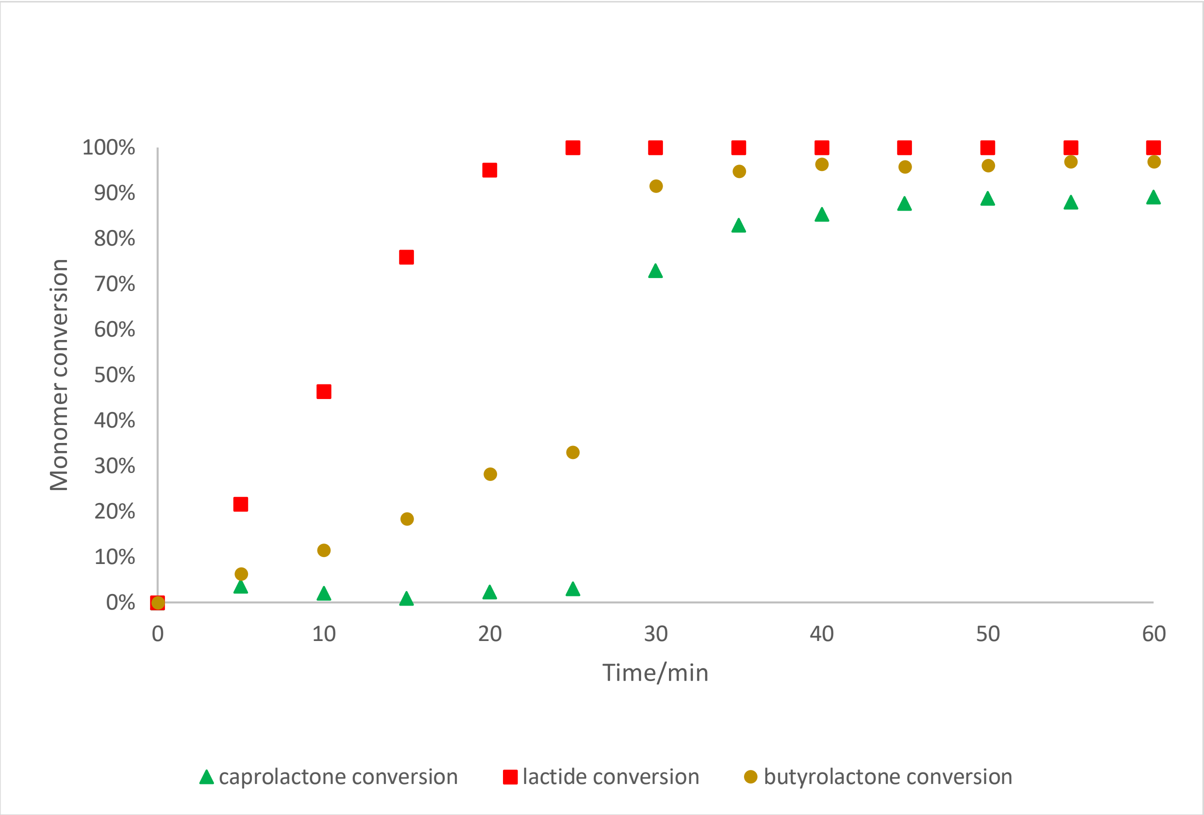

[a]Reaction conditions: [*rac*-LA]$_0$=[*rac*-β-BL]$_0$=[ε-CL]$_0$) = 0.25 M in 8 mL toluene at ambient temperature, [Monomer]$_0$/[**Y**]= 200, reaction time 1 h.

Figure S62: ^{1}H DOSY NMR Spectrum obtained after mixing a copolymer with 20 wt% PLA homopolymer

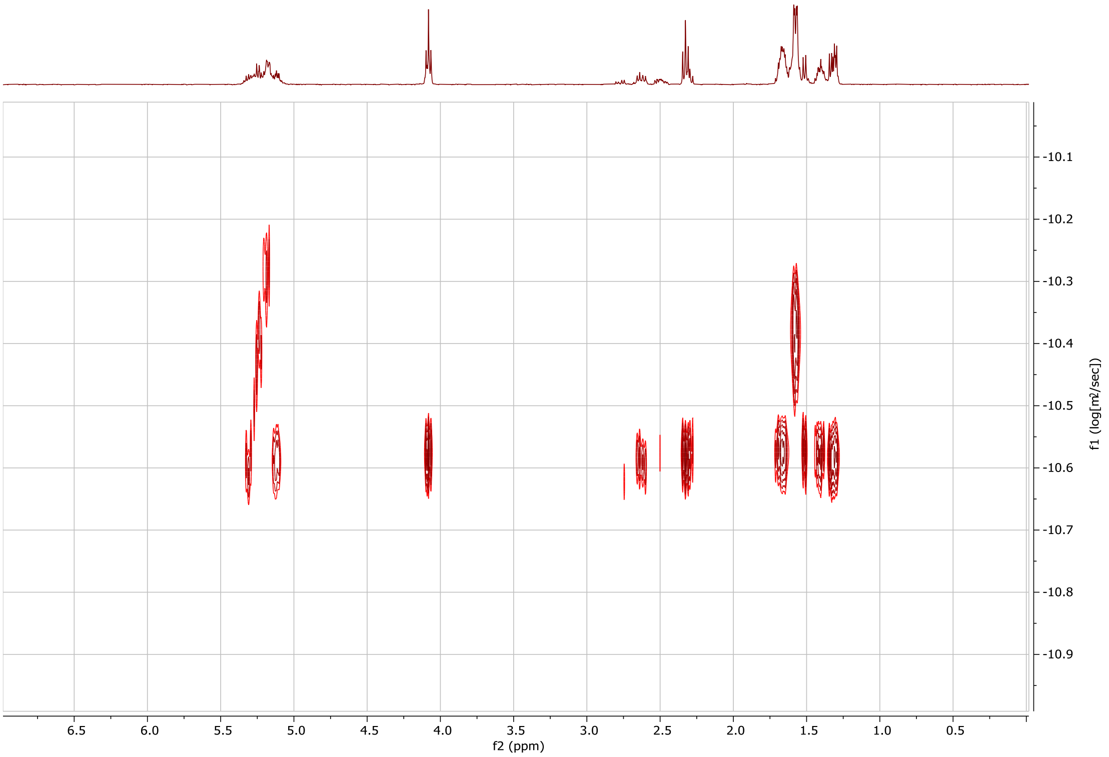

Figure S63: ^{1}H DOSY NMR Spectrum obtained after mixing a copolymer with 40 wt% PLA homopolymer

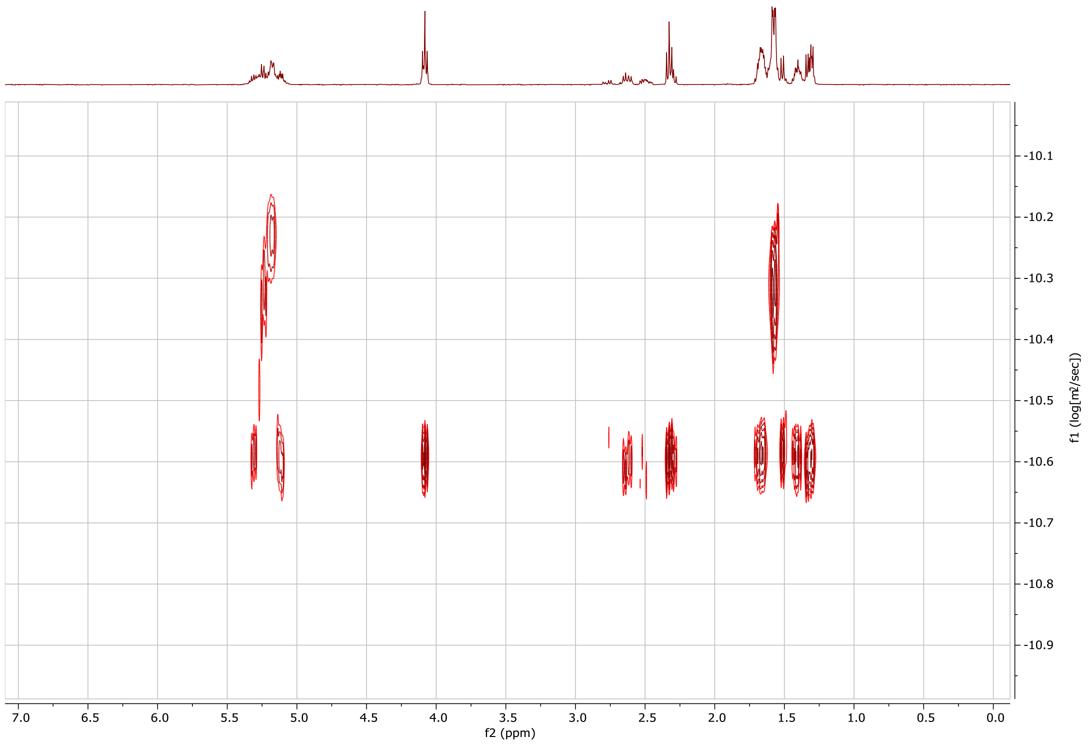

Figure S64: ^{1}H DOSY NMR Spectrum obtained after mixing a copolymer with 60 wt% PLA homopolymer

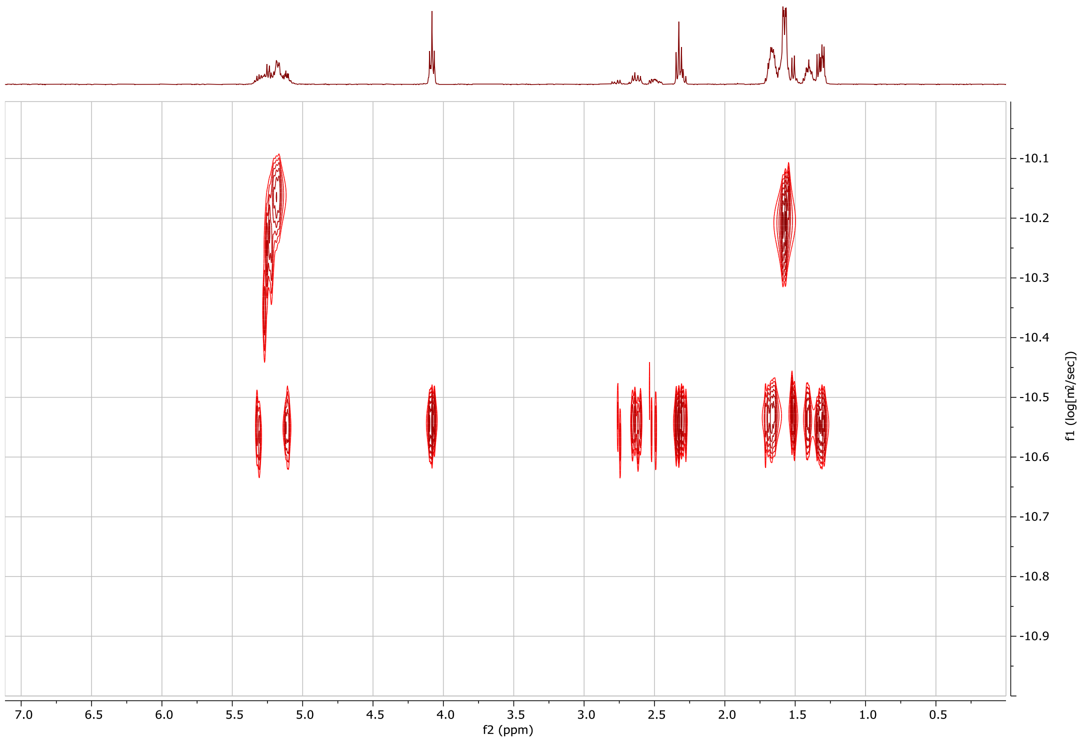

Figure S65: ^{1}H DOSY NMR Spectrum obtained after mixing a copolymer with 100 wt% PLA homopolymer

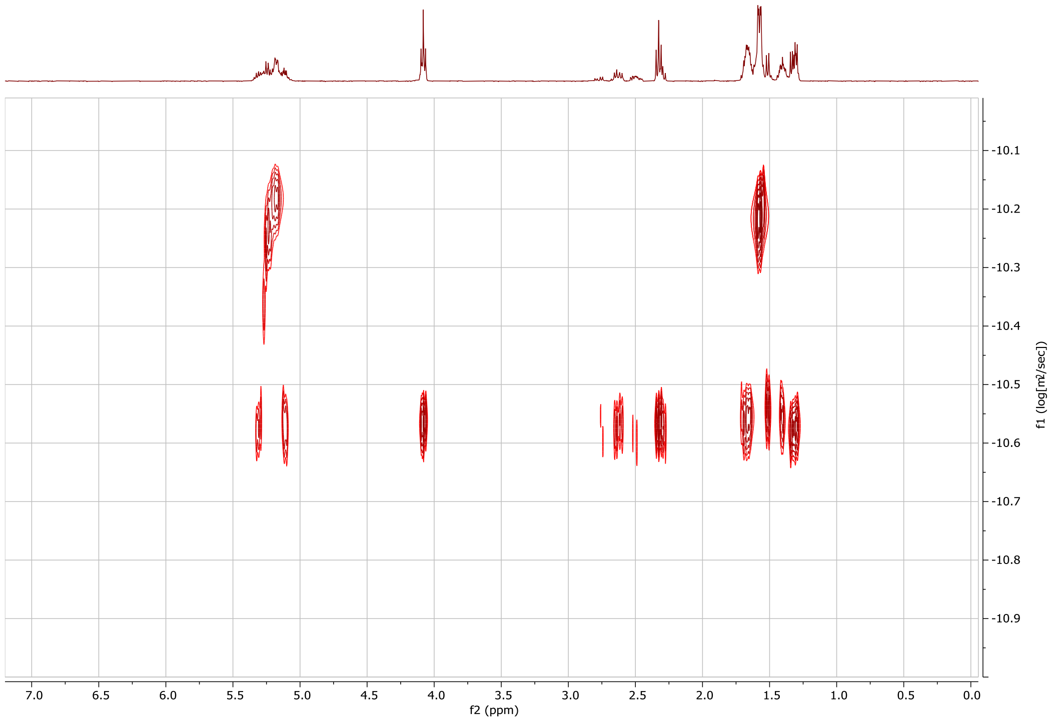

Figure S66: ^{1}H DOSY NMR Spectrum of PLA homopolymer

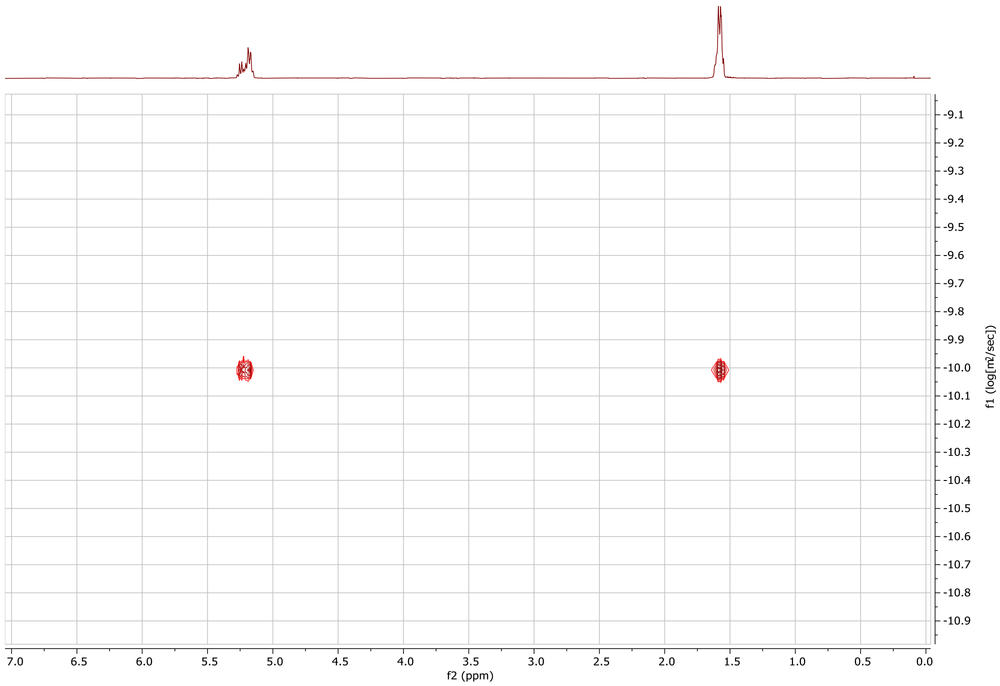

Table S3: Comparison of diffusion co-efficients obtained from the spectra in Figs S62 – S66

Amount of homopolymer added (wt%)	Estimated diffusion co-efficient of PLA homopolymer, D /log(m^2/s)	Estimated diffusion co-efficient of copolymer, D /log(m^2/s)

0	-	-10.5
20	-10.4	-10.6
40	-10.3	-10.6
60	-10.2	-10.5
100	-10.2	-10.5
Homopolymer alone	-10.0	-

Figure S67: ^{1}H DOSY NMR Spectrum obtained after mixing a copolymer with 20 wt% PCL homopolymer

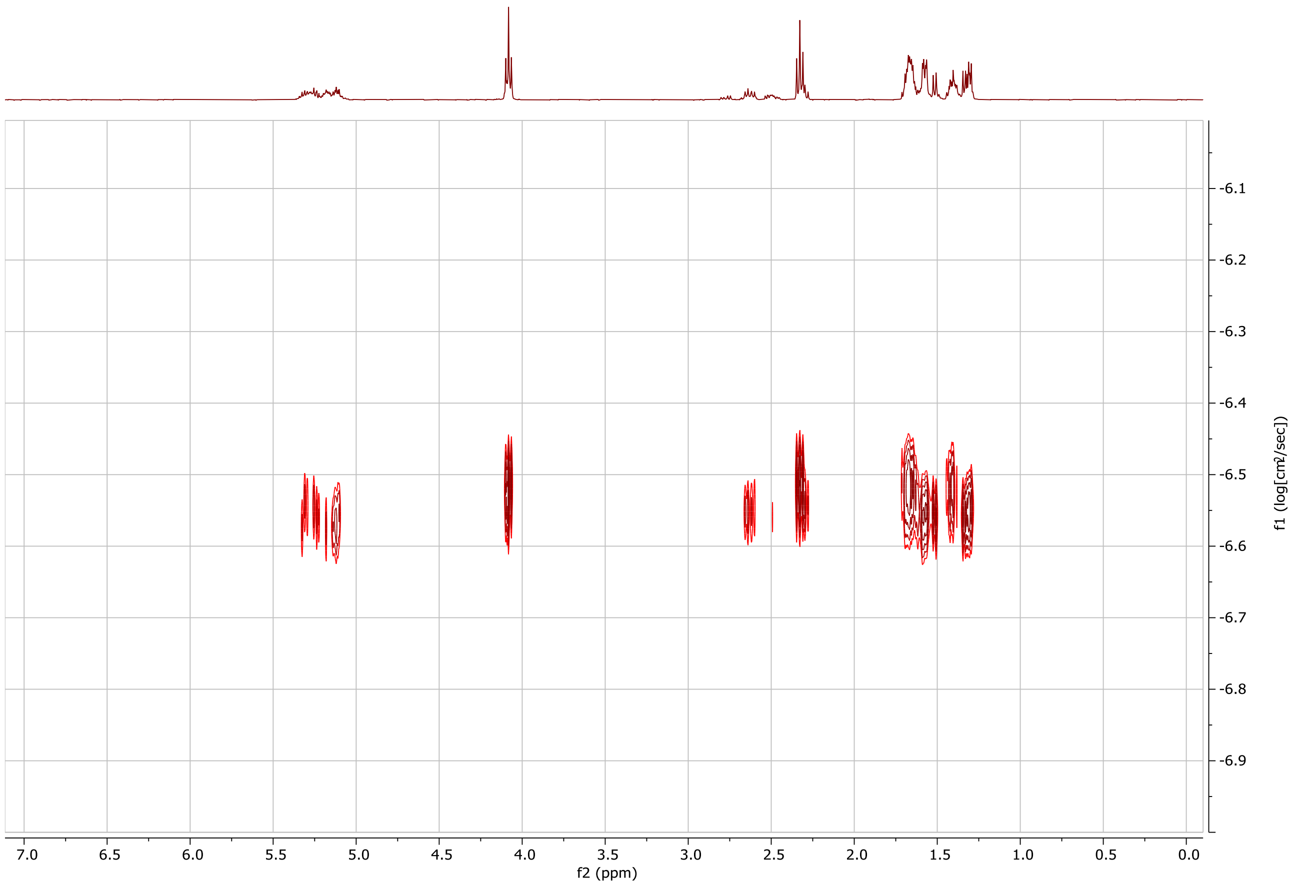

Figure S68: ^1H DOSY NMR Spectrum obtained after mixing a copolymer with 40 wt% PCL homopolymer

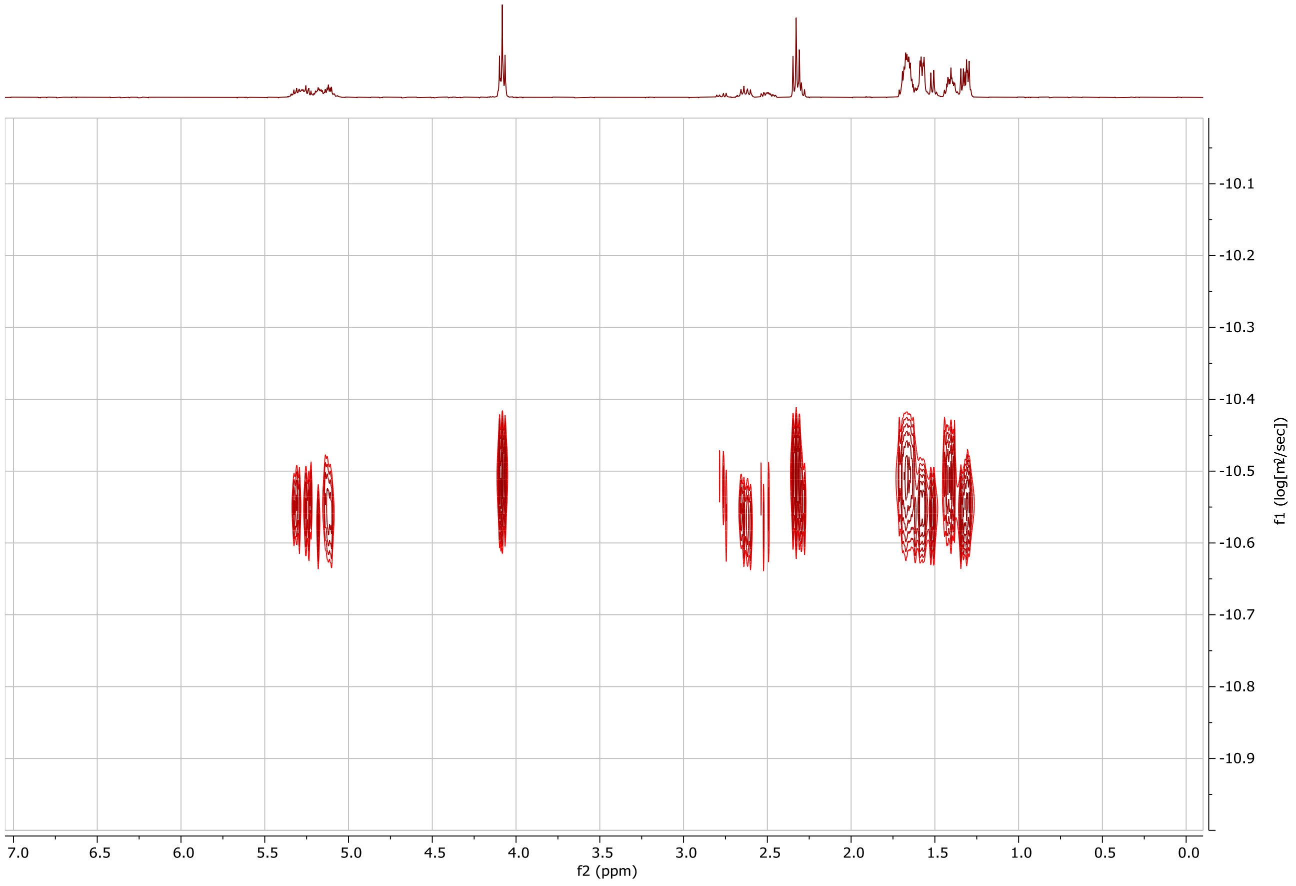

Figure S69: ^{1}H DOSY NMR Spectrum obtained after mixing a copolymer with 60 wt% PCL homopolymer

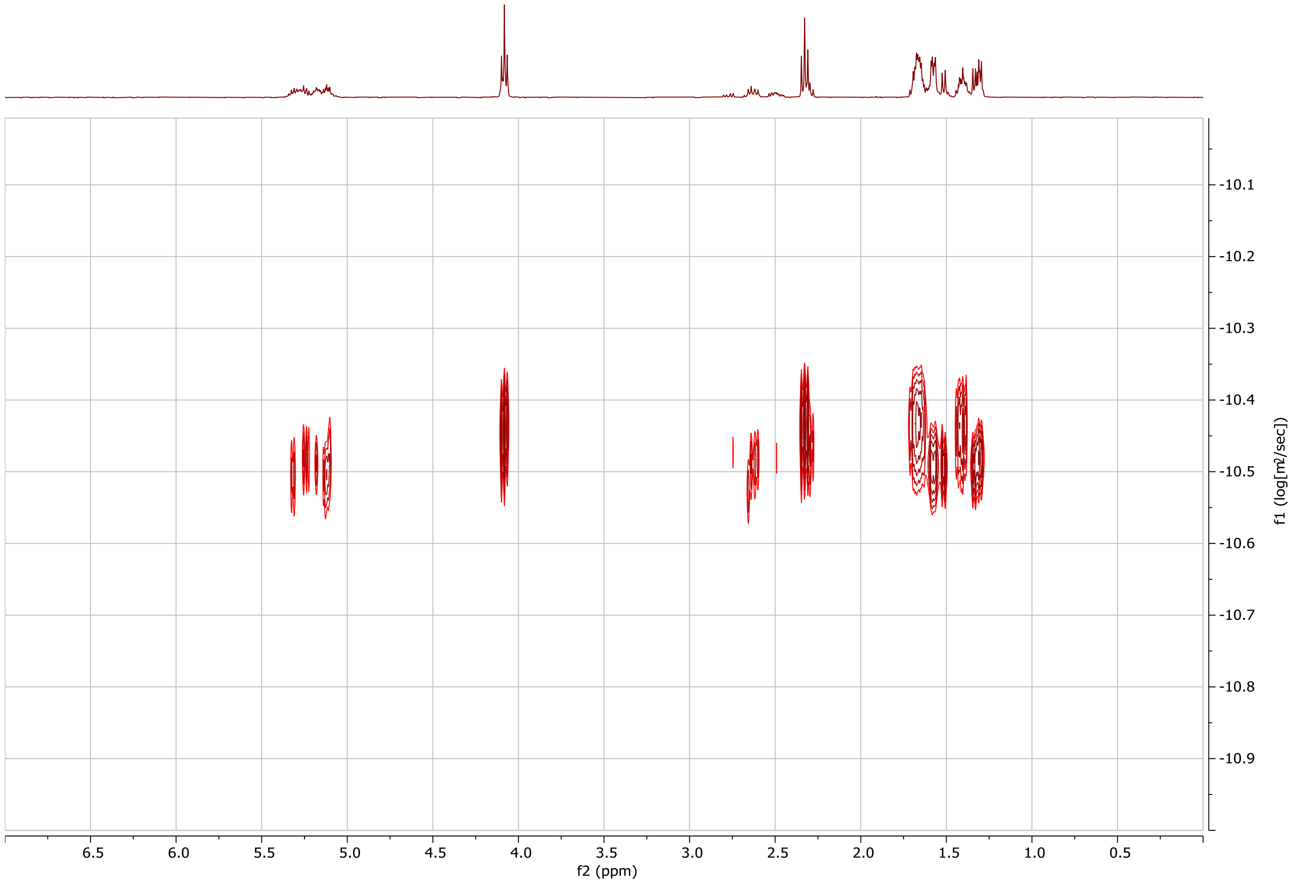

Figure S70: ^{1}H DOSY NMR Spectrum obtained after mixing a copolymer with 100 wt% PCL homopolymer

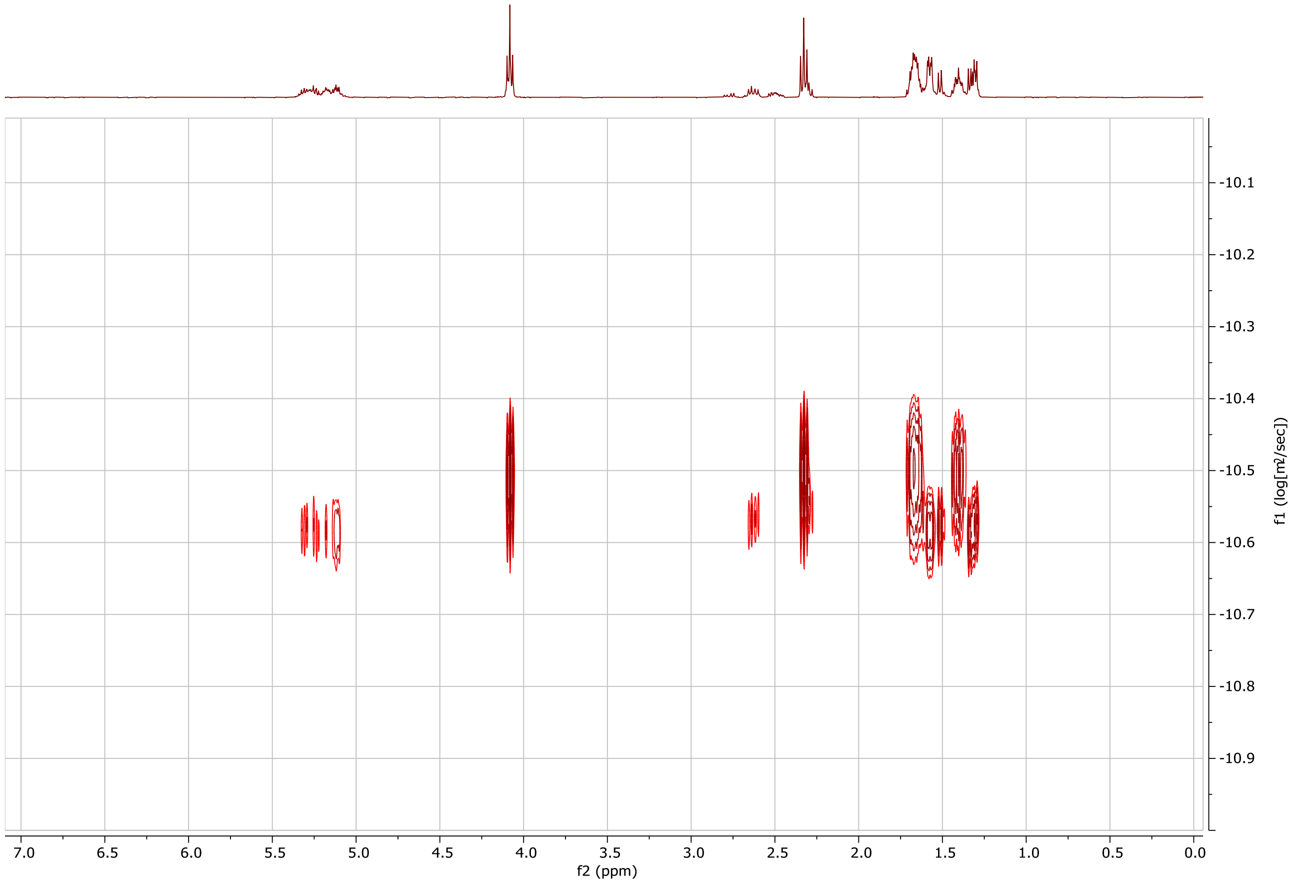

Figure S71: ^{1}H DOSY NMR Spectrum of PCL homopolymer

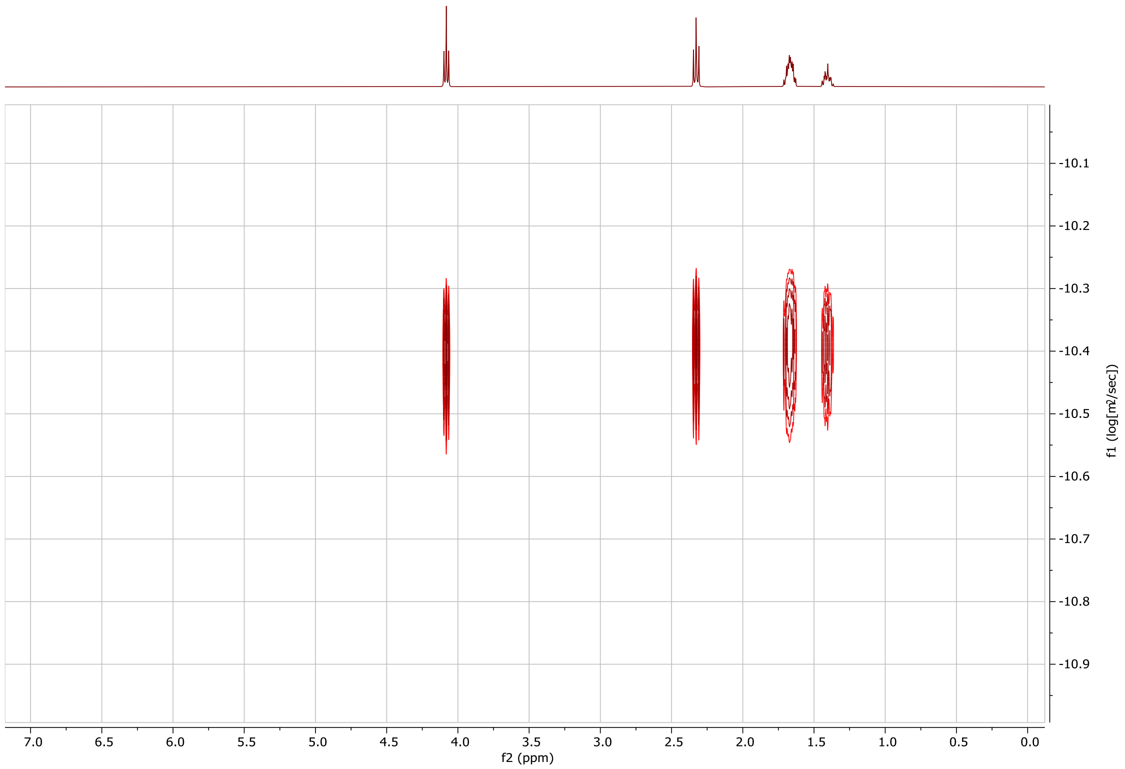

Table S4: Comparison of diffusion co-efficients obtained from the spectra in Figs S67 – S71

Amount of homopolymer added (wt%)	Estimated diffusion co-efficient of PCL homopolymer, D /log(m^2/s)	Estimated diffusion co-efficient of copolymer, D /log(m^2/s)
0	-	-10.5
20	10.5	-10.6
40	10.5	-10.6
60	10.5	-10.6
100	10.5	-10.6
Homopolymer alone	10.4	-

Figure S72: ^{1}H DOSY NMR Spectrum obtained after mixing a copolymer with 20 wt% P3HB homopolymer

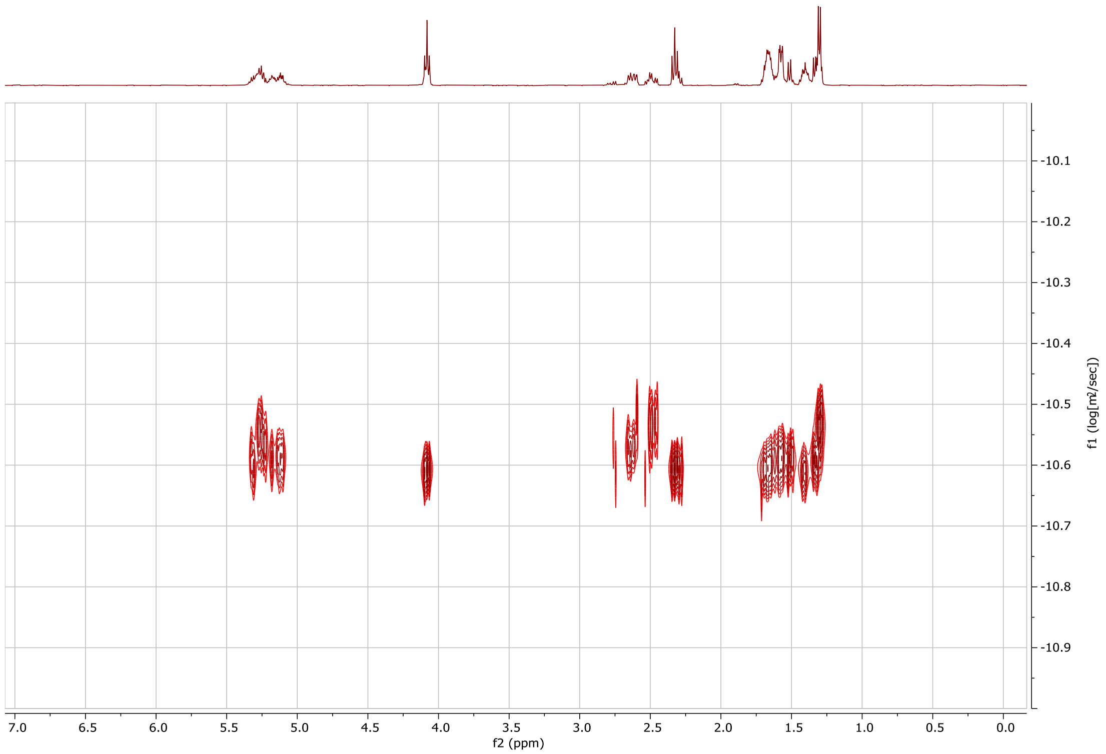

Figure S73: ^{1}H DOSY NMR Spectrum obtained after mixing a copolymer with 60 wt% P3HB homopolymer

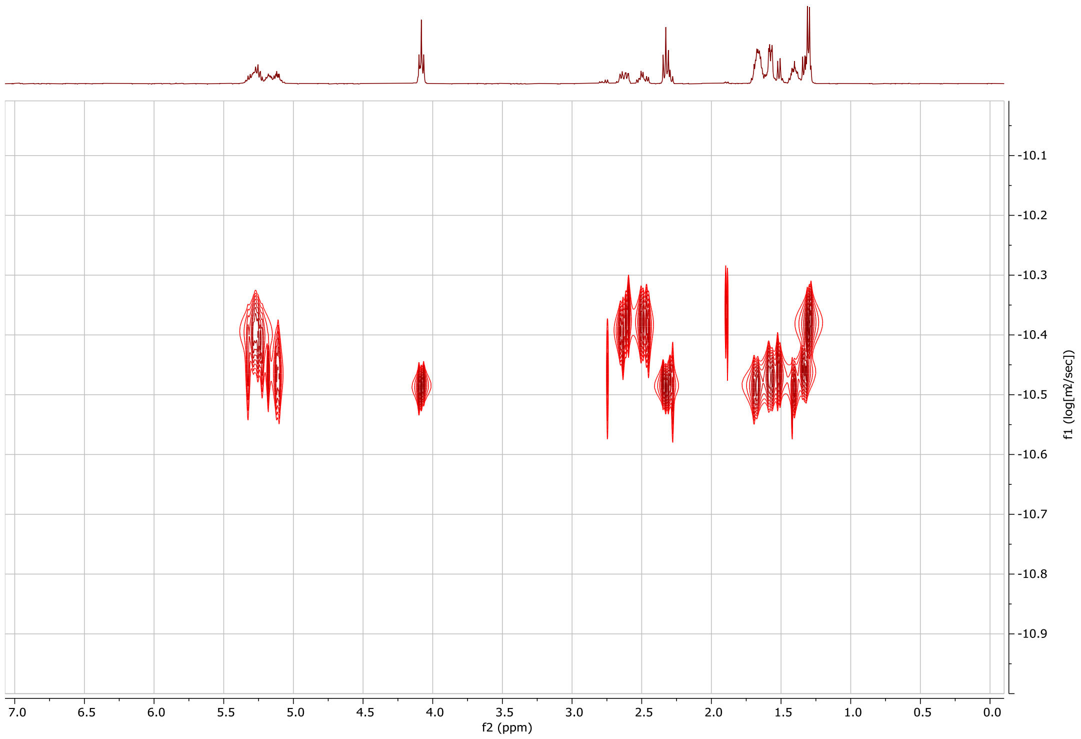

Figure S74: ^{1}H DOSY NMR Spectrum obtained after mixing a copolymer with 100 wt% P3HB homopolymer

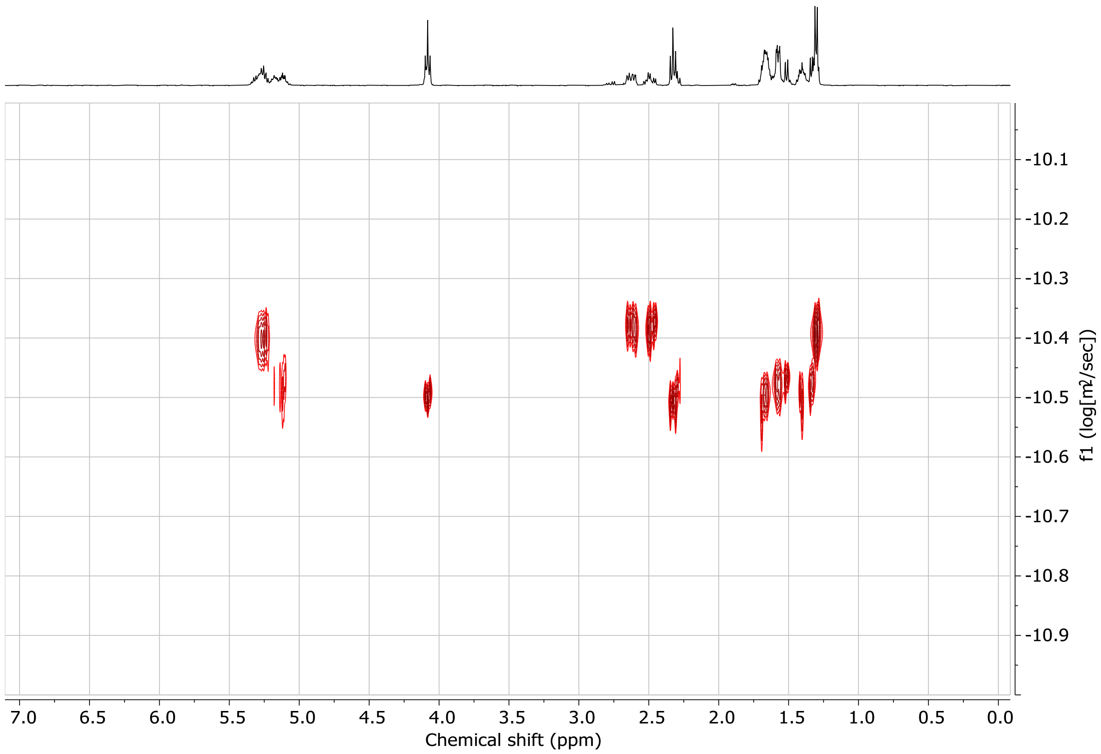

Figure S75: ^{1}H DOSY NMR Spectrum of P3HB homopolymer

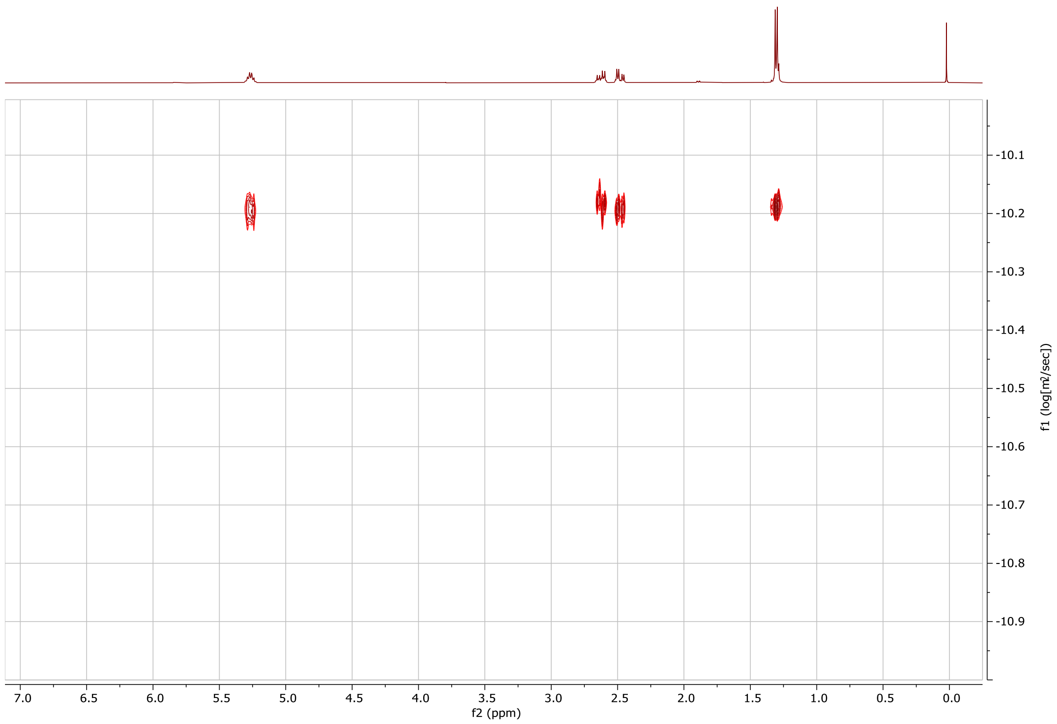

Table S3: Comparison of diffusion co-efficients obtained from the spectra in Figs S72 – S75

Amount of homopolymer added (wt%)	Estimated diffusion co-efficient of P3HB homopolymer, D /log(m^2/s)	Estimated diffusion co-efficient of copolymer, D /log(m^2/s)
0	-	-10.5
20	-10.5	-10.6
60	-10.4	-10.5
100	-10.4	-10.5
Homopolymer alone	-10.2	-

1. M. Bero, J. Kasperczyk, G. Adamus, *Die Makromol. Chemie* **1993**, *194*, 907–912.
2. J. Fagerland, A. Finne-Wistrand, D. Pappalardo, *New J. Chem.* **2016**, *40*, 7671–7679.
3. R. H. Platel, A. R. Hurst, *Macromolecules* **2020**, *53*, 10773–10784.
4. H. Abe, Y. Doi, H. Aoki, T. Akehata, Y. Hori, A. Yamaguchi, *Macromolecules* **1995**, *28*, 7630–7637.
